# Goondicones A–H: Spiro-Isoindolinone Heartworm Anthelmintics from an Australian Pasture-Soil-Derived *Streptomyces* sp.

**DOI:** 10.3390/antibiotics13121222

**Published:** 2024-12-17

**Authors:** Jianying Han, David F. Bruhn, Cynthia T. Childs, Yovany Moreno, Angela A. Salim, Taizong Wu, Robert J. Capon

**Affiliations:** 1 Institute for Molecular Bioscience, The University of Queensland, St. Lucia, QLD 4072, Australia; jianying.han@uq.edu.au (J.H.); a.salim@uq.edu.au (A.A.S.); 2 Boehringer Ingelheim Animal Health, USA Inc., 1730 Olympic Drive, Athens, GA 30601, USA; david.bruhn@boehringer-ingelheim.com (D.F.B.); cynthia.childs@boehringer-ingelheim.com (C.T.C.); yovany.moreno@boehringer-ingelheim.com (Y.M.); 3 Key Laboratory of Marine Genetic Resources, Third Institute of Oceanography, Ministry of Natural Resources, 184 Daxue Road, Xiamen 361005, China; wutaizong@tio.org.cn

**Keywords:** anthelmintic, citizen science, *Dirofilaria immitis*, microbial natural product, spiro-isoindolinone, Soils for Science, *Streptomyces*

## Abstract

Background/Objectives: There is an urgent need for new and improved anthelmintics that are not constrained by existing resistance pathways and that can safeguard the health and welfare of animals. Methods: An integrated platform of chemical, bioassay, and cultivation profiling applied to a library of microbes isolated from Australian livestock pasture soil was used to detect and guide the production, isolation, characterization, identification, and evaluation of new natural products with anthelmintic properties. Results: A global natural products social (GNPS) molecular network analysis of 110 Australian pasture-soil-derived microbial extracts prioritized for antiparasitic activity identified unique molecular families in the extract of *Streptomyces* sp. S4S-00185A06, a strain selectively active against *Dirofilaria immitis* microfilariae. UPLC-DAD analysis identified metabolites with unique UV-vis chromophores and unprecedented molecular formulas. A chemical investigation of *Streptomyces* sp. S4S-00185A06 yielded goondicones A–H (**1**–**8**) as new examples of a rare class of spiro-isoindolinones, with structures assigned on the basis of detailed spectroscopic analysis, ECD calculations, and biosynthetic considerations. Conclusions: While goondicones **1**–**8** exhibit little to no in vitro inhibitory activity against Gram-positive, Gram-negative, and/or fungal pathogens, human carcinoma cells, or the livestock gastrointestinal parasite *Haemonchus contortus* L1–L3 larvae, **5** and **6** (and, to a lesser extent, **1**) inhibit the motility of heartworm *Dirofilaria immitis* microfilaria (IC_50_ 10–11 μM). A structure activity relationship analysis based on the co-metabolites **1**–**8** suggests that (i) an 8-OH is preferable to 8–oxo moiety, (ii) 20-NMe and 3-OH moieties are essential, and (iii) C-9 epimerization exerts no discernible impact on in vitro potency.

## 1. Introduction

Historically, soil microbes with their rich repertoire of secondary metabolites have proven to be a valuable source of bioactive lead molecules, inspiring the discovery and development of some of the world’s most successful anti-infective products (i.e., antibiotics, antiparasitics) and safeguarding human and animal health and welfare, as well as improving productivity and sustainability across many areas of agriculture [1]. Notable success stories include the discovery and commercial development of soil *Streptomyces*-derived macrolactone anthelmintics (i.e., avermectins), which have been the gold standard for parasite control for nearly a half century [2].

Notwithstanding past success, the effectiveness of existing agricultural and veterinary anthelmintic products is increasingly challenged by the widespread emergence of drug resistance, spanning all major anthelmintic classes (i.e., benzimidazoles, macrocyclic lactones, and imidazothiazoles) [3,4,5]. As a result, there is an urgent and compelling need for new and improved classes of anthelmintics. In designing a strategy to achieve this goal, we speculated that prospects for success could be enhanced by focusing on (i) the largely untapped molecular potential of the Australian soil microbiome and (ii) microbes isolated from livestock pasture soils infected by pathogenic nematodes, where the latter could drive the evolution of microbial chemical defenses against predatory early life stage nematode larvae.

To implement this strategy, we assembled a library of Australian livestock-pasture-derived bacteria and fungi. Solvent extracts prepared from solid-phase agar plate cultivations of these microbes were subjected to bioassay and prioritized based on their ability to inhibit the motility of microfilariae (mf) from the companion animal heartworm *Dirofilaria immitis* and/or L1–L3 larvae from the livestock gastrointestinal nematode *Haemonchus contortus* (>75% at 25 μg/mL). Prioritized extracts were also subjected to chemical profiling (GNPS) [6] to dereplicate new from known and rare from common, as well as to eliminate false positives (i.e., natural product classes that have been determined to have no commercial potential due to unacceptable mechanisms of action, cytotoxicity, poly-pharmacology, etc.). Microbes associated with the most promising extracts were subjected to cultivation profiling (MATRIX) [7] to select for media and growth conditions that optimize the production of target chemistry. Subsequent scaled-up cultivation, extraction and fractionation, followed by spectroscopic (and chemical) characterization and structure elucidation permitted the identification of new anthelmintic natural products and related co-metabolites.

**Figure 1:**
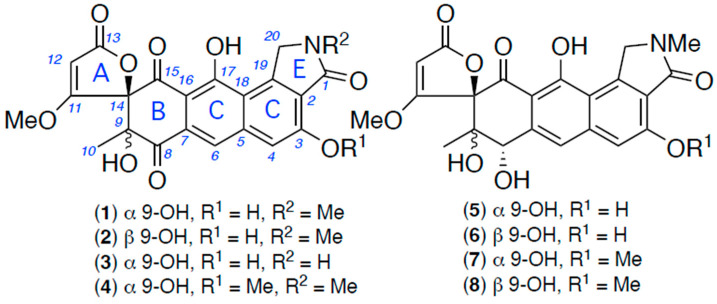
*Streptomyces* sp. S4S-00185A06 metabolites, goondicones A–H (**1**–**8**).

In recent years, our implementation of this strategy has benefited from access to an extensive library of microbes isolated under the auspices of the Australian citizen science initiative, Soils for Science (S4S) [8], a nationwide program that to date has engaged >5000 members of the public to provide >15,000 backyard soils rich in microbial diversity across urban, rural, and native ecosystems. Although still in the very early discovery phase, S4S soil-derived microbes have already delivered multiple new classes of natural products exhibiting anthelmintic properties. For example, a pilot study of 30 soil samples collected from Goondicum Station, a cattle property situated in an extinct volcanic crater near the headwaters of the Burnett River, Queensland, Australia, returned 417 chemically distinct microbes/extracts (i.e., no replicates), of which 110 were prioritized as inhibiting motility *D. immitis* mf and/or *H. contortus* L1–L3 larvae. To date, we have reported on the chemical analysis of three of these microbes, describing the *D. immitis* inhibitory carbocyclic *ansa*-polyketide goondomycins A–H from *Streptomyces* sp. S4S-00052A05 [9] and terpenyl-quinolin-4(1H)-one goondolinones A and B from *Actinomadura* sp. S4S-00245B09 [10], as well as the *D. immitis* and *H. contortus* inhibitory polyketide goondapyrones A–J from *Streptomyces* sp. S4S-00196A10 [11]. This current report describes a chemical investigation of *Streptomyces* sp. S4S-00185A06 that yielded the new *D. immitis* inhibitory spiro-isoindolinone goondicones A–H (**1**–**8**) (Figure 1). What follows is an account of the detection, optimized production, isolation, characterization, and structure elucidation of **1**–**8**, together with a commentary on plausible biosynthesis and anthelmintic potential.

## 2. Results

A global natural products social (GNPS) [6] molecular network analysis of 110 microbial extracts prioritized as inhibiting the motility of *D. immitis* mf and/or the development of *H. contortus* L1–L3 larvae detected two molecular families unique to the IPS2 extract of *Streptomyces* sp. S4S-00185A06 (Figure S4B), a strain that exhibited selective activity against *D. immitis* mf. A UPLC-DAD analysis of this extract detected a suite of natural products with unusual UV-vis chromophores (Figure S4C) and molecular formulas unprecedented in the microbial natural products scientific literature (*m*/*z* 440, C_22_H_17_NO_9_; *m*/*z* 442, C_22_H_19_NO_9_; *m*/*z* 454, C_23_H_19_NO_9_; *m*/*z* 456, C_23_H_21_NO_9_). Application of a 24-well plate miniaturized cultivation profiling approach (MATRIX) [7] to S4S-00185A06, employing 11 different media compositions under solid agar, as well as static and shaken broth conditions, revealed that production of the target natural products was highly media-dependent and optimal under ISP2 solid agar conditions (Figures S5 and S6). Subsequent scaled-up ISP2 solid (100 plate) cultivation was subjected to solvent extraction and trituration, followed by reversed phase HPLC to yield goondicones A–H (**1**–**8**), with structures assigned through detailed spectroscopic analysis, as outlined below.

**Figure 2:**
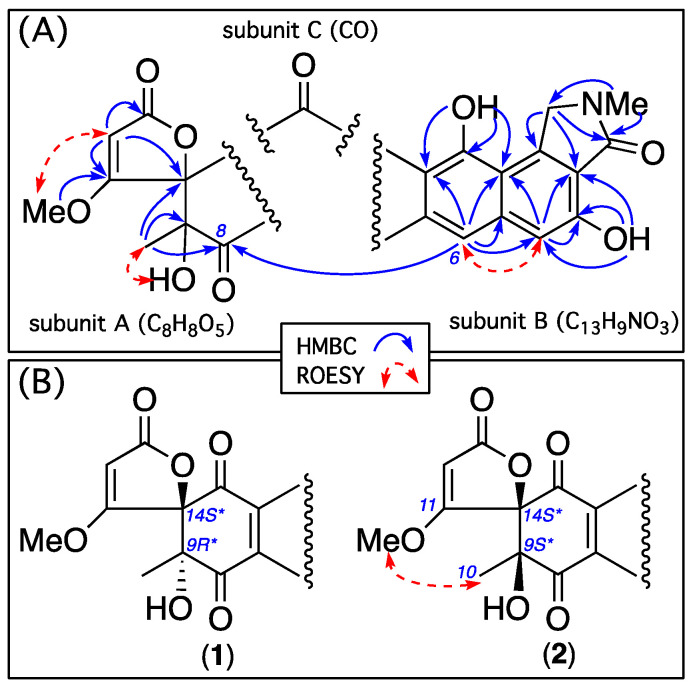
Selected 2D NMR (DMSO-*d*_6_) correlations for **1**–**2** designating (**A**) common subunits A–C and connectivity and (**B**) a diagnostic ROESY correlation for **2** that defines relative configurations for **1** and **2**.

**Figure 3:**
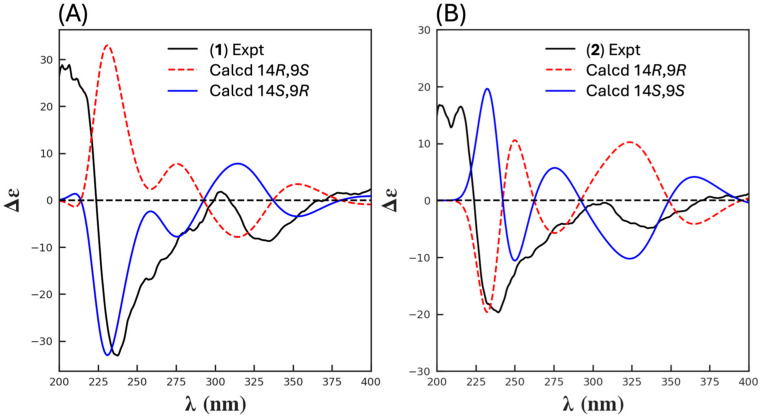
(**A**) ECD spectra for (**1**), experimental (black), calculated 14*R*,9*S* (red dashed), and calculated 14*S*,9*R* (blue). (**B**) ECD spectra for (**2**), experimental (black), calculated 14*R*,9*R* (red dashed), and calculated 14*S*,9*S* (blue).

HRESIMS measurements established the isomeric molecular formula for **1** (C_22_H_17_NO_9_, *m*/*z* M+Na Δmmu − 0.1) and **2** (C_22_H_17_NO_9_, *m*/*z* M+Na Δmmu +1.6) requiring 15 double bond equivalents (DBE). Analysis of the NMR (DMSO-*d*_6_) data for **1** and **2** (Table 1, Table 2, Tables S2 and S3 and Figure 2 and Figures S7–S17) revealed resonances attributed to two chelated phenols (**1**, δ_H_ 10.84/13.38; **2**, δ_H_ 10.77/13.23), a tertiary OH (**1**, δ_H_ 6.49; **2**, δ_H_ 6.99), an OMe (**1**, δ_H_ 3.69, δ_C_ 61.1; **2**, δ_H_ 3.76, δ_C_ 61.4), an NMe (**1**, δ_H_ 3.14, δ_C_ 28.8; **2**, δ_H_ 3.14, δ_C_ 28.7), and two sp^2^ lactone/lactam (**1**, δ_C_ 166.0/170.9; **2**, δ_C_ 166.1/170.8) and two sp^2^ ketone carbonyls (**1**, δ_C_ 194.8/192.5; **2**, δ_C_ 191.4/192.8), as well as a further three sp^2^ methines and nine sp^2^ quaternary carbons, accounting for all heteroatoms and ten DBE and requiring that **1** and **2** be pentacyclic. Notwithstanding that the ^1^H NMR data for **1** and **2** lacked any ^1^H-^1^H coupling, the 2D NMR HMBC and ROESY data permitted the assembly of subunits A–C, which accounted for the molecular formula, with an HMBC from H-6 to C-8 permitting connectivity to arrive at identical planar structures for **1** and **2** (Figure 2A), necessitating that they be stereoisomers. A ROESY correlation between H_3_-10 and the 11-OMe in **2** (but not **1**) required that these moieties be cis disposed to each other and thereby defined a 14S*,9R* configuration for goondicone A (**1**) and 14S*,9S* for goondicone B (**2**) (Figure 1 and Figure 2B), which was further supported by energy-minimized conformations of **1** and **2** (Figure S19). Comparison of experimental and calculated ECD spectra (Figure 3, Tables S10 and S11) permitted assignment of the absolute configurations for goondicones A (**1**) and B (**2**), as shown.

**Figure 4:**
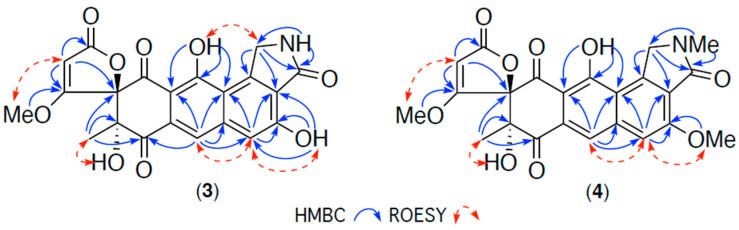
Selected 2D NMR (DMSO-*d*_6_) correlations for **3**–**4**.

HRESIMS measurements established molecular formulas for **3** (C_21_H_15_NO_9_, *m*/*z* M+Na Δmmu − 0.6) and **4** (C_23_H_19_NO_9_, *m*/*z* M+Na Δmmu +0.5) consistent with lower (–CH_2_) and higher (+CH_2_) homologues of **1**. Comparison of the NMR (DMSO-*d*_6_) data for **3** (Table 1, Table 2 and Table S4 and Figure 4 and Figures S20–S24) with **1** revealed the principle difference to be the replacement of the 20-NMe in **1** with a 20-NH in **3** (δ_H_ 8.95), further confirmed by HMBC correlations from the 20-NH to C-1 and C-20, and a significant deshielding of C-20 in **3** (δ_C_ 47.4) relative to B (δ_C_ 53.6). Significantly, a selection of deshielded NMR chemical shifts proved diagnostic in differentiating between the 14*S*,9*R* configuration in **1** (δ_C_ 194.8, C-8; 22.4, C-10) versus the 14*S*,9*S* configuration in **2** (δ_C_ 191.4, C-8; 16.2, C-10). When applied to goondicone C (**3**) (δ_C_ 194.9, C-8; 22.4, C-10), the chemical shift trend confirmed a 14S*,9R* relative configuration, as shown (Figure 1). Comparison of the NMR (DMSO-*d*_6_) data for **4** (Table 1, Table 2 and Table S5 and Figure 4 and Figures S26–S30) with **1** revealed the principle difference as the inclusion of a 3-OMe moiety (δ_H_ 4.03; δ_C_ 56.2), with key NMR chemical shifts for goondicone D (**4**) (δ_C_ 194.8, C-8; 22.4, C-10) indicative of a 14S*,9R* relative configuration, in common with **1** and **3**. Absolute configurations were assigned to goondicones C (**3**) and D (**4**), as shown, based on comparable ECD spectra with the co-metabolite **1** (Figure S58) and biosynthetic considerations.

**Figure 5:**
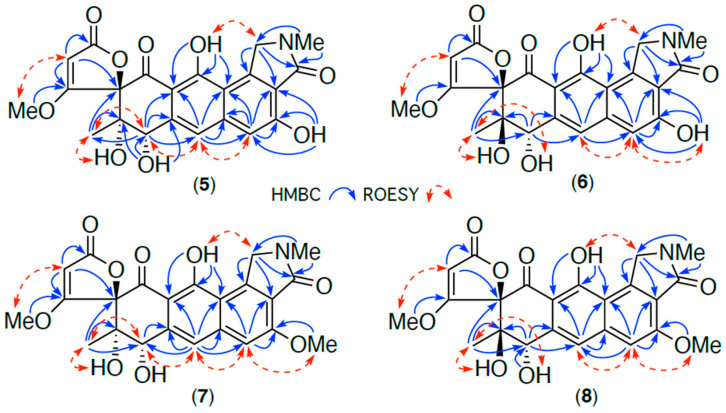
Selected 2D NMR (DMSO-*d*_6_) correlations for **5**–**8**.

**Figure 6:**
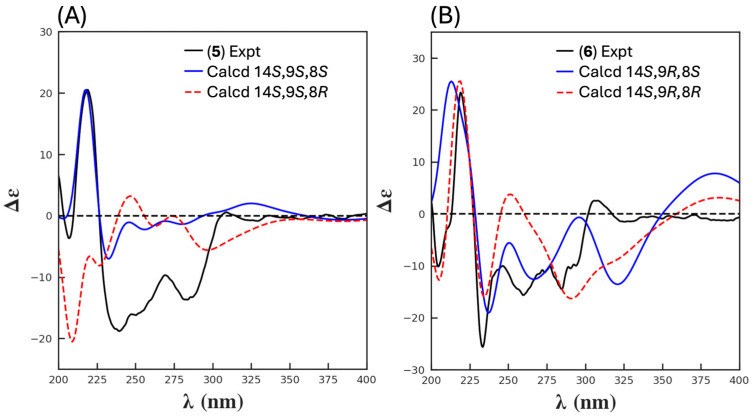
(**A**) ECD spectra for (**5**), experimental (black), calculated 14*S*,9*S*,8*R* (red dashed), and calculated 14*S*,9*S*,8*S* (blue). (**B**) ECD spectra for (**6**), experimental (black), calculated 14*S*,9*R*,8*R* (red dashed), and calculated 14*S*,9*R*,8*S* (blue).

HRESIMS measurements established the isomeric molecular formula for **5** (C_22_H_19_NO_9_, *m*/*z* M+Na Δmmu +1.1) and **6** (C_22_H_19_NO_9_, *m*/*z* M+Na Δmmu − 0.6) consistent with reduced (+H_2_) analogues of **1** and **2**. Comparison of the 1D and 2D NMR (DMSO-*d*_6_) data for **5** and **6** (Table 3, Table 4, Tables S6 and S7 and Figure 5 and Figures S32–S42) with **1** and **2** revealed a high level of concordance, with the principle differences attributed to the reduction of the C-8 carbonyl to an 8-OH in **5** (δ_H_ 5.72, d, *J* 9.3 Hz, 8-OH; 5.03, d, *J* 9.3 Hz, H-8; δ_C_ 69.7, C-8) and **6** (δ_H_ 6.13, d, *J* 6.2 Hz, 8-OH; 5.30, d, *J* 6.2 Hz, H-8; δ_C_ 69.5, C-8). Based on biosynthetic grounds, it seemed plausible that the isomerism between **5** and **6** was associated with C-9 epimers, as noted above for **1** and **2**. In an effort to test this hypothesis, and in the absence of useful ROESY correlations (i.e., between H_3_-10 and 11-OMe), we turned our attention to the NMR chemical shift differences noted above for **1** and **2**. More specifically, where chemical shift deshielding was observed in **2** relative to **1** for both C-10 (δ_C_ −6.2) and H_3_-10 (δ_H_ − 0.06), this same deshielding trend was also observed in **6** relative to **5** for both C-10 (δ_C_ − 1.7) and H_3_-10 (δ_H_ − 0.16). These observations, together with biosynthetic considerations (see below), supported a 14S*,9S* relative configuration for goondicone E (**5**) and 14S*,9R* for goondicone F (**6**), as shown (Figure 1 and Figure 4). Comparison of experimental and calculated ECD spectra (Figure 6 and Tables S12–S15) permitted the assignment of the absolute configurations for goondicones E (**5**) and F (**6**), as shown.

HRESIMS measurements established isomeric molecular formulas for **7** (C_23_H_21_NO_9_, *m*/*z* M+Na Δmmu +0.4) and **8** (C_23_H_21_NO_9_, *m*/*z* M+Na Δmmu +1.0) consistent with methylated (+CH_2_) analogues of **5** and **6**. Comparison of the 1D and 2D NMR (DMSO-*d*_6_) data for **7** and **8** (Table 3, Table 4, Tables S8 and S9 and Figure 5 and Figures S44–S54) with **5** and **6** revealed a high level of concordance, with the principle difference attributed to conversion of the 3-OH to a 3-OMe moiety in both **7** (δ_H_ 3.99; δ_C_ 56.0) and **8** (δ_H_ 3.99; δ_C_ 56.0). Likewise, deshielding of the NMR chemical shifts in **8** relative to **7** for C-10 (δ_C_ − 1.7) and H_3_-10 (δ_H_ − 0.16), together with biosynthetic considerations (see below), supported a 14S*,9S* relative configuration for goondicone G (**7**) and 14S*,9R* for goondicone H (**8**), as shown (Figure 1 and Figure 5). Absolute configurations were assigned to goondicones G (**7**) and H (**8**), as shown, based on comparable ECD spectra with the co-metabolites **5** and **6** (Figure S59) and biosynthetic considerations.

**Figure 7:**
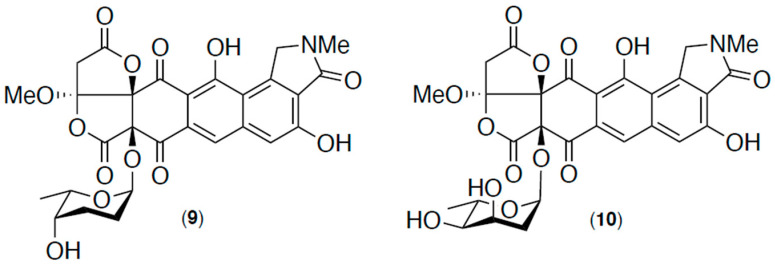
Known spiro-isoindolinone natural products **9** and **10**.

The goondicones A–H (**1**–**8**) belong to a rare class of spiro-isoindolinones for which there are only two previously known examples: lactonamycin (**9**), reported in 1996 [12] from the Japanese soil-derived *Streptomyces rishirensis* MJ7773-88K4, and for which an X-ray analysis was reported in 1999 [13,14], and lactonamycin Z (**10**), reported in 2003 [15] from the UK soil-derived *Streptomyces sanglieri* Strain AK 623 (Figure 7). While subsequent reports in 2008 [16] and 2013 [17] described the biosynthesis and synthesis of **9**–**10**, to date, there are no further accounts of additional members of this rare class of natural product.

**Figure 8:**
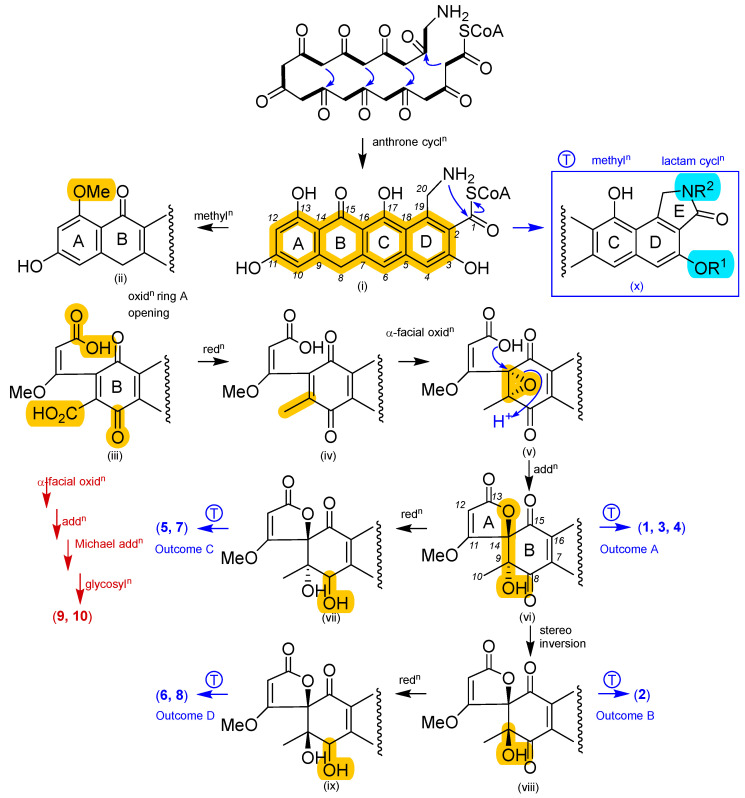
Plausible biosynthesis of spiro-isoindolinones **1**–**10**.

The goondicones A–H (**1**–**8**) are likely the product of an NRPS-PKS with a glycine starter unit annotated by nine acetate units (Figure 8) undergoing a sequence of transformations inclusive of a cascade anthrone cyclization to (i), selective 13-OH methylation to (ii), oxidative ring A cleavage at the C-10/C-11 bond to (iii), selective reduction of C-10 to (iv), α-facial oxidation of Δ^9,14^ to the epoxide (v), and intramolecular addition to the spiro system (vi). At this point, (vi) can be transformed to deliver four outcomes (A–D). Outcome A involves methylation and lactam-mediated termination from the NRPS-PKS to yield **1**, **3**, and **4**. Outcome B involves selective reduction of (vi) to (vii) followed by termination to **5** and **7**. Outcome C involves C-9 stereo-inversion of (vi) to (viii) followed by termination to **2**. Outcome D involves selective reduction of (viii) to (ix) followed by termination to **6** and **8**. This proposed goondicone biosynthetic pathway also provides a logical branch where omission of the reductive conversion of (iii) to (iv) retains the C-10 carboxylic acid, allowing for a subsequent α-facial oxidation of Δ^9,14^, addition to the epoxide, intra-molecular Michael addition to the fused five-membered lactone system, and glycosylation to yield **9** and **10** (Figure 8, brown highlight). As epimerization, oxidation, and methylation are established pathways for the transformation of natural products to artifacts [18], the natural product status of **1**–**8** was affirmed by both their detection in the crude extract prior to fractionation and the failure to observe any interconversions during handling and storage, including deliberate exposure of **5** to harsh conditions (Figures S56 and S57).

## 3. Discussion

The goondicones A–H (**1**–**8**) did not exhibit growth inhibitory activity against either the Gram-negative bacteria *Escherichia coli* ATCC 11775 or the fungus *Candida albicans* ATCC 10231, nor did they exhibit cytotoxicity towards human colorectal (SW620) or lung (NCI-H460) carcinoma cells or inhibit the motility of *H. contortus* L1–L3 larvae (Table 5, Figures S60 and S61). They were also largely inactive against the Gram-positive bacteria *Staphylococcus aureus* ATCC 25923 and *Enterococcus faecalis* ACM 5184, with the exception of **1** (and, to a lesser degree, **2** and **5**), which exhibited modest levels of antibacterial activity (Table 5). Notwithstanding their overall lack of cellular toxicity, selected goondicones accounted for the activity exhibited by *Streptomyces* sp. S4S-00185A06 against *D. immitis* mf, with the preliminary structure activity relationship analysis revealing that (a) both the 20-NMe and 3-OH functionalities were essential, (b) analogues bearing an 8-OH were ~2-fold more active than those featuring an 8-oxo, and (c) inversion of the C-9 configuration had little impact on the anthelmintic activity. Although exhibiting only modest levels of potency, an understanding the goondicones mechanism of action could inform future efforts at developing anthelmintics uncompromised by existing resistance pathways.

## 4. Materials and Methods

For general experimental details, see the Supplementary Materials.

### 4.1. Collection of Soils and Isolation of Microbes

Soil samples (n = 30) collected under the auspices of the Australian Soils for Science (S4S) citizen science initiative [8] from Goondicum Station, located in an extinct volcanic crater near the Burnett River headwaters in Queensland, Australia, were used to inoculate ISP2 and M1 agar mother plates. After 14 days of incubation at 27 °C, 494 isolates were manually picked and cultivated on fresh ISP2 or M1 agar plates, corresponding to their original medium. The isolates were photographed, with the images uploaded to the S4S Gallery [19]. They were cryopreserved at −80 °C and extracted with EtOAc, and the resulting dried extracts were resuspended in DMSO and archived at −20 °C.

### 4.2. Chemical Profiling (UPLC-DAD and UPLC-QTOF)

EtOAc extracts from ISP2 or M1 agar cultivations of S4S soil-derived microbes underwent UPLC-DAD and UPLC-QTOF chemical profiling. For UPLC-DAD chemical profiling, 2 μL of each extract (~1 mg/mL in MeOH) was injected into an Agilent 1290 Infinity UPLC system (Santa Clara, CA, USA) equipped with a Zorbax SB-C8 RRHD column (1.8 μm, 50 × 2.1 mm). The gradient elution (0.417 mL/min over 2.5 min) transitioned from 90% H_2_O/MeCN to 100% MeCN, with 0.01% TFA as a modifier, and analytes were detected using a diode array detector. For UPLC-QTOF chemical profiling, 1 μL of each extract (~100 μg/mL in MeOH) was analyzed using an Agilent 1290 Infinity II UPLC system (Santa Clara, CA, USA) with the same column and gradient conditions but with 0.1% formic acid/MeCN as the modifier. Detection was performed using an Agilent 6545 Q-TOF mass spectrometer (Santa Clara, CA, USA).

### 4.3. Chemical Profiling (GNPS Molecular Networking)

MS/MS data acquisition was carried out in positive ion mode at a collision energy of 35 eV. Raw Agilent MassHunter data files (.d format) were converted to mzXML format using MSConvert software and uploaded to the GNPS server [20]. Molecular networking was performed using the GNPS data analysis workflow [6] with a spectral clustering algorithm, applying a cosine similarity score threshold of 0.7 and a minimum of six matched peaks. The resulting molecular network was imported into Cytoscape version 3.8.0 [21] using a ball–stick layout, where nodes represented parent masses, edge thickness indicated cosine scores, and node pie charts displayed group abundances, reflecting the intensity of MS signals. MS/MS fragmentation analysis targeted ions with intensities above 200 counts in the full scan range using 5 scans/second, ~4 *m*/*z* isolation width, fixed collision energy, and a maximum of three precursors per cycle. General instrument parameters included a gas temperature of 325 °C, drying gas flow at 10 L/min, nebulizer pressure at 20 psi, a sheath gas temperature of 400 °C, fragmentation voltage of 180 V, and a skimmer voltage of 45 V.

### 4.4. Taxonomy of S4S-00185A06

Genomic DNA was extracted from an ISP2 agar plate cultivation of S4S-00185A06 using the DNeasy Blood & Tissue Kit (Qiagen, Hilden, Germany) following the manufacturer’s protocol. The 16S rRNA gene was amplified through PCR using the universal primers 27F (5′-AGAGTTTGATCCTGGCTCAG-3′) and 1492R (5′-TACGGCTACCTTCTTACGACTT-3′) obtained from Sigma-Aldrich (St. Louis, MO, USA). The PCR mixture (50 μL) consisted of 2 μL of genomic DNA (20–40 ng), 25 μL of EmeraldAmp GT PCR Master Mix (Kusatsu, Shiga, Japan) (2X Premix), 0.2 μM of each primer, and H_2_O to a final volume of 50 μL. PCR conditions included an initial denaturation at 95 °C for 2 min, followed by 40 cycles of 95 °C for 20 s (denaturation), 56 °C for 20 s (annealing), and 72 °C for 30 s (extension), with a final extension at 72 °C for 5 min. The resulting PCR products were purified with a PCR purification kit (Qiagen) and sequenced.

### 4.5. Phylogenetic Analysis of S4S-00185A06

A phylogenetic tree was generated using PhyML Maximum Likelihood analysis was based on the top twenty-five most similar 16S rRNA sequences retrieved from a BLAST search of the RefSeq RNA NCBI database, with S4S-00185A06 16S rRNA as the query. The JC69 model was applied for phylogenetic inference [22]. Sequence alignments were performed with the MUSCLE program [23]. A phylogenetic tree was constructed using the UGENE program using the aforementioned models and visualized using Ugene’s tree view [24]. BLAST analysis (NCBI database) of the amplified 16S rRNA sequence revealed 98.3% identity with *Streptomyces caeruleus* OS3-4 (accession number: OR976176) (Figures S1–S3).

### 4.6. Cultivation Profiling (MATRIX)

S4S-00185A06 was cultured in a 24-well plate (MATRIX) [7] system using 11 different media compositions (Table S1). Cultivations were conducted in solid agar (1.5 g) and liquid broth formats (1.5 mL) under static and shaken conditions (190 rpm) and incubated at 27 °C for 10 days. An additional set of control incubations was conducted with the same 11 media compositions but without inoculation. Individual MATRIX wells (Figure S5) were extracted in situ with 2 mL of EtOAc. The organic phases were dried at 40 °C under a nitrogen stream and then re-suspended in 100 mL of MeOH. One portion of the extract was analyzed using the GNPS chemical profiling method (as described above), while another portion was spiked with an internal calibrant (1-decyloxy-2,4-dinitrobenzene, 50 μg/mL) and subjected to UPLC-DAD chemical profiling (as described above) (Figure S6).

### 4.7. Scale-Up Cultivation and Purification of S4S-00185A06

A seed culture of S4S-00185A06 was prepared by inoculating 40 mL of ISP2 broth medium and incubating at 190 rpm and 27 °C for 5 days. Aliquots (100 μL) of the seed culture were streaked onto individual ISP2 agar plates (×100), and after incubation at 27 °C for 20 days, the recovered agar was diced and extracted with EtOAc (3 × 500 mL) and the combined organic phase was concentrated in vacuo to yield an extract (1169 mg). The MeOH soluble portion of this extract (746 mg) was subjected to preparative reversed-phase HPLC (Zorbax RX-C_8_ 7 μm, 21.2 × 250 mm column, 20 mL/min gradient elution over 20 min from 90% H_2_O/MeCN to 100% MeCN, with a constant 0.01% TFA/MeCN modifier) to give **1** (11.64 mg, 1.6%) and a further 40 fractions. Fraction 15 (8.06 mg) was subjected to semi-preparative HPLC (Zorbax RX-C_8_ 5 μm, 250 × 9.4 mm column, 3 mL/min gradient elution over 20 min from 75% H_2_O/MeCN to 65% H_2_O/MeCN, with a constant 0.01% TFA/MeCN modifier) to yield **4** (0.92 mg, 0.12%). Fraction 17 (4.06 mg) was purified on semi-preparative HPLC (Zorbax RX-C_8_ 5 μm, 250 × 9.4 mm column, 3 mL/min gradient elution over 20 min from 80% H_2_O/MeCN to 40% H_2_O/MeCN, with a constant 0.01% TFA/MeCN modifier) to afford **2** (2.33 mg, 0.3%). Combined fractions 13 and 14 (34.47 mg) were subjected to semi-preparative HPLC (Zorbax RX-C_8_ 5 μm, 250 × 9.4 mm column, 3 mL/min gradient elution over 20 min from 80% H_2_O/MeCN to 75% H_2_O/MeCN, with a constant 0.01% TFA/MeCN modifier) to yield **5** (1.64 mg, 0.22%), **7** (1.13 mg, 0.15%), **3** (1.79 mg, 0.24%), and a mixed subfraction. The latter was further purified on semi-preparative HPLC (Phenomenex Luna-C_8_ 5 μm, 250 × 9.4 mm column, 3 mL/min gradient elution over 20 min from 80% H_2_O/MeCN to 70% H_2_O/MeCN, with a constant 0.01% TFA/MeCN modifier) to give **6** (0.66 mg, 0.09%) and **8** (0.59 mg, 0.08%). (Note: all % yields are weight to weight estimates based on the unfractionated EtOAc extract).

Goondicone A (**1**). Yellowish powder; [α]^22^_D_ −60 (*c* 0.02, MeOH); 1D and 2D NMR (DMSO-*d*_6_) (Table 1, Table 2, and Table S2; Figure 2 and Figures S7–S11); HRESIMS *m*/*z* 462.0795 [M + Na]^+^ (calculated for C_22_H_17_NNaO_9_, 462.0796).

Goondicone B (**2**). Yellowish powder; [α]^22^_D_ –114 (*c* 0.02, MeOH); 1D and 2D NMR (DMSO-*d*_6_) (Table 1, Table 2, and Table S3; Figure 2 and Figures S13–S17); HRESIMS *m*/*z* 462.0812 [M + Na]^+^ (calculated for C_22_H_17_NNaO_9_, 462.0796).

Goondicone C (**3**). Yellowish powder; [α]^22^_D_ −64 (*c* 0.02, MeOH); 1D and 2D NMR (DMSO-*d*_6_) (Table 1, Table 2, and Table S4; Figure 3 and Figures S20–S24); HRESIMS *m*/*z* 448.0633 [M + Na]^+^ (calculated for C_21_H_15_NNaO_9_, 448.0639).

Goondicone D (**4**). Yellowish powder; [α]^22^_D_ –93 (*c* 0.02, MeOH); 1D and 2D NMR (DMSO-*d*_6_) (Table 1, Table 2, and Table S5; Figure 3 and Figures S26–S30); HRESIMS *m*/*z* 476.0957 [M + Na]^+^ (calculated for C_23_H_19_NNaO_9_, 476.0952).

Goondicone E (**5**). Yellowish powder; [α]^22^_D_ −168 (*c* 0.02, MeOH); 1D and 2D NMR (DMSO-*d*_6_) (Table 3, Table 4, and Table S6; Figure 4 and Figures S32–S36); HRESIMS *m*/*z* 464.0963 [M + Na]^+^ (calculated for C_22_H_19_NNaO_9_, 464.0952).

Goondicone F (**6**). Yellowish powder; [α]^22^_D_ −96 (*c* 0.02, MeOH); 1D and 2D NMR (DMSO-*d*_6_) (Table 3, Table 4, and Table S7; Figure 4 and Figures S38–S42); HRESIMS *m*/*z* 464.0946 [M + Na]^+^ (calculated for C_22_H_19_NNaO_9_, 464.0952).

Goondicone G (**7**). Yellowish powder; [α]^22^_D_ −133 (*c* 0.02, MeOH); 1D and 2D NMR (DMSO-*d*_6_) (Table 3, Table 4, and Table S8; Figure 4 and Figures S44–S48); HRESIMS *m*/*z* 478.1113 [M + Na]^+^ (calculated for C_23_H_21_NNaO_9_, 478.1109).

Goondicone H (**8**). Yellowish powder; [α]^22^_D_ +16.2 (*c* 0.03, MeOH); 1D and 2D NMR (DMSO-*d*_6_) (Table 3, Table 4, and Table S9; Figure 4 and Figures S50–S54); HRESIMS *m*/*z* 478.1119 [M + Na]^+^ (calculated for C_23_H_21_NNaO_9_, 478.1109).

### 4.8. ECD Calculations

Conformational analysis was initially performed using random searching in Stochastic, employing the MMFF94 force field with an energy cut-off of 7.0 kcal/mol and an RMSD threshold of 0.2 Å. All conformers were then optimized consecutively at the PM6 and HF/6-31G(d) levels. The dominant conformers were further optimized at the B3LYP/6-31G(d) level in the gas phase. The optimized conformers exhibited no imaginary frequencies, confirming them as true local minima. ECD calculations were carried out at the B3LYP/6-311G(d,p) level in MeOH with the IEFPCM model using Time-Dependent Density Functional Theory (TD-DFT). The ECD spectrum was simulated by overlapping Gaussian functions for each transition.

### 4.9. Antibacterial, Antifungal, and Cytotoxicity Assays

The assays were performed on **1**–**8** using the methodology detailed in the Supporting Information (Figures S60 and S61).

### 4.10. Antiparasitic Assays

Inhibition of motility of *D. immitis* microfilariae. Approximately 250 *D. immitis* microfilariae were suspended in 100 µL of RPMI 1640 medium (Hyclone) and added to wells of a microtiter plate containing test compounds formulated in 100% DMSO. For dose–response experiments, compounds were delivered to generate 10–3.2-fold serially diluted doses, covering the range of 25 to 0.0007 µg/mL. The final DMSO concentration in the assay was 0.5%. Plates were incubated for ~72 h at 37 °C and 5% CO_2_. Parasite motility was assessed using a camera-based system, and quantitative descriptors were calculated for each well. Compound efficacy at a given dose was expressed as the percentage of motility inhibition after normalization with the average motility of positive (1.0 µM of gramicidin) and negative (DMSO) controls on each plate. EC_50_ values were calculated using Boehringer Ingelheim’s MEGALAB application using a four-parameter logistic model.

*H. contortus* L1-L3 larvae development assay (LDA). Approximately 20 *H. contortus* L1-stage larvae were added to wells of a microtiter plate containing nutrient medium and test compounds previously dissolved in 100% DMSO, resulting in a final assay volume of 100 µL. Each compound was delivered to generate 10–3.2-fold serially diluted doses to cover the range of 25–0.0007 µg/mL. The DMSO assay concentration was 0.5%. Plates were incubated at 27 °C and 85% relative humidity for 4 days. The resulting L3 larvae were imaged using a camera-based system to assess motility, as described above. Compound efficacy was carried out through normalization with the average motility of wells containing positive (1.0 µM of ivermectin) and negative controls (DMSO) on each plate. EC_50_ values were calculated with Boehringer Ingelheim’s MEGALAB application using a four-parametric logistic model.

## 5. Conclusions

This study demonstrates the value of exploring Australian pasture-soil-derived microbes as a source of new natural products with novel molecular structures and anthelmintic potential. More specifically, chemical analysis of the cattle station pasture-soil-derived *Streptomyces* sp. S4S-00185A06 returned goondicones A–H (**1**–**8**) as new examples of a rare class of spiro-isoindolinones, the only known close analogues being lactonamycin (**9**) and lactonamycin Z (**10**). A structure activity relationship assessment revealed that while **1**–**8** lacked antibiotic properties and were not cytotoxic to human carcinoma cells or the livestock gastrointestinal nematode *H. contortus*, **5** and **6** (and, to a lesser extent, **1**) did inhibit heartworm *D. immitis* mf motility. Notwithstanding that the anti-heartworm potency of the latter was relatively low, the SAR analysis did open the prospect of a selective and potentially new mechanism of action, knowledge of which might inform the development of future anthelmintics.

## Figures and Tables

**Table 1:** ^1^H NMR (DMSO-*d*_6_) data for goondicones A–D (**1**–**4**).

Position	1 δ_H_, Mult. (*J* Hz)	2 δ_H_, Mult. (*J* Hz)	3 δ_H_, Mult. (*J* Hz)	4 δ_H_, Mult. (*J* Hz)
4	7.52, s	7.50, s	7.52, s	7.82, s
6	8.07, s	8.05, s	8.08, s	8.19, s
10	1.40, s	1.34, s	1.40, s	1.41, s
12	5.58, s	5.68, s	5.58, s	5.59, s
20	4.97, s	*a.* 4.99, AB_q_ (20.2)	4.89, s	4.95, s
		*b.* 4.95, AB_q_ (20.2)		
3-OH	10.84, s	10.77, s	10.73, s	-
3-OMe	-	-	-	4.03, s
9-OH	6.49, s	6.99, s	6.49, br s	6.52, s
11-OMe	3.69, s	3.76, s	3.69, s	3.69, s
17-OH	13.38, s	13.23, s	13.35, s	13.31, s
20-NH	-	-	8.95, s	-
20-NMe	3.14, s	3.14, s	-	3.12, s

**Table 2:** ^13^C NMR (DMSO-*d*_6_) data for goondicones A–D (**1**–**4**).

Position	1 *δ*_C_, Type	2 *δ*_C_, Type	3 *δ*_C_, Type	4 *δ*_C_, Type
1	166.0, C	166.1, C	169.0, C	164.7, C
2	122.0, C	121.9, C	121.8, C	123.1, C
3	157.5, C	157.2, C	157.9, C	158.7, C
4	112.9, CH	112.7, CH	112.7, CH	109.4, CH
5	140.7, C	140.7, C	141.1, C	140.8, C
6	119.2, CH	119.4, CH	119.2, CH	119.4, CH
7	129.5, C	130.5, C	129.6, C	129.8, C
8	194.8, C	191.4, ^a^ C	194.9, C	194.8, C
9	78.3, C	77.6, C	78.3, C	78.3, C
10	22.4, CH_3_	16.2, ^b^ CH_3_	22.4, CH_3_	22.4, CH_3_
11	177.8, C	177.2, C	177.8, C	177.7, C
12	89.9, CH	90.4, CH	90.4, CH	89.9, CH
13	170.9, C	170.8, C	171.0, C	170.9, C
14	90.4, C	90.0, C	90.4, C	90.5, C
15	192.5, C	192.8, C	192.5, C	192.7, C
16	108.0, C	108.1, C	108.0, C	108.6, C
17	163.6, C	162.8, C	163.6, C	163.2, C
18	115.3, C	115.2, C	115.7, C	115.9, C
19	145.0, C	144.8, C	147.3, C	145.2, C
20	53.6, CH_2_	53.6, CH_2_	47.4, CH_2_	53.1, CH_2_
3-OMe	-	-	-	56.2, CH_3_
11-OMe	61.1, CH_3_	61.4, CH_3_	61.1, CH_3_	61.1, CH_3_
20-NMe	28.8, CH_3_	28.7, CH_3_	-	28.9, CH_3_

^a^ Detected through HMBC. ^b^ Detected through HSQC.

**Table 3:** ^1^H NMR (DMSO-*d*_6_) data for goondicones E–H (**5**–**8**).

Position	5 *δ*_H_, Mult. (*J* Hz)	6 *δ*_H_, Mult. (*J* Hz)	7 *δ*_H_, Mult. (*J* Hz)	8 *δ*_H_, Mult. (*J* Hz)
4	7.17, s	7.19, s	7.43, s	7.46, s
6	7.48, s	7.49, s	7.65, s	7.66, s
8	5.03, d (9.3)	5.30, d (6.2)	5.06, br s	5.33, d (6.1)
10	1.28, s	1.12, s	1.29, s	1.13, s
12	5.67, s	5.60, s	5.68, s	5.61, s
20	*a.* 4.95, AB_q_ (20.2)	*a.* 4.93, AB_q_ (20.2)	*a.* 4.92, AB_q_ (20.2)	*a.* 4.91, AB_q_ (20.2)
	*b.* 4.90, AB_q_ (20.2)	*b.* 4.89, AB_q_ (20.2)	*b.* 4.86, AB_q_ (20.2)	*b.* 4.86, AB_q_ (20.2)
3-OH	10.42, s	10.50, s	-	-
3-OMe	-	-	3.99, s	3.99, s
8-OH	5.72, d (9.3)	6.13, d (6.2)	5.77, br s	6.20, d (6.3)
9-OH	5.74, s	5.88, s	5.77, br s	5.90, s
20-NMe	3.12, s	3.11, s	3.09, s	3.09, s
11-OMe	3.82, s	3.80, s	3.83, s	3.80, s
17-OH	13.77, s	13.84, s	13.70, s	13.78, s

**Table 4:** ^13^C NMR (DMSO-*d*_6_) data for goondicones E–H (**5**–**8**).

Position	5 *δ*_C_, type	6 *δ*_C_, type	7 *δ*_C_, type	8 *δ*_C_, type
1	166.6, C	166.5, C	165.2, C	165.1, C
2	119.5, C	119.6, C	120.7, C	120.8, C
3	156.8, C	157.1, C	158.3, C	158.6, C
4	109.9, CH	110.1, CH	106.7, CH	106.9, CH
5	142.0, C	142.0, C	142.1, C	142.1, C
6	115.8, CH	115.5, CH	116.4, CH	116.1, CH
7	141.7, C	140.5, C	142.1, C	141.0, C
8	69.7, CH	69.5, CH	69.7, CH	69.5, CH
9	77.0, C	76.6, C	77.0, C	76.7, C
10	18.7, CH_3_	17.0, CH_3_	18.7, CH_3_	17.0, CH_3_
11	178.6, C	179.7, C	178.6, C	179.6, C
12	90.6, CH	90.4, CH	90.7, CH	90.4, CH
13	171.5, C	171.5, C	171.5, C	171.5, C
14	89.7, C	90.6, C	89.8, C	90.7, C
15	194.9, C	194.5, C	195.1, C	194.8, C
16	108.0, C	107.6, C	108.4, C	108.1, C
17	163.4, C	164.2, C	163.1, C	163.8, C
18	112.4, C	112.4, C	112.7, C	112.7, C
19	144.9, C	145.1, C	145.2, C	145.4, C
20	53.7, CH_2_	53.6, CH_2_	53.1, CH_2_	53.1, CH_2_
3-OMe	-	-	56.0, CH_3_	56.0, CH_3_
11-OMe	61.1, CH_3_	60.7, CH_3_	61.1, CH_3_	60.7, CH_3_
20-NMe	28.6, CH_3_	28.7, CH_3_	28.8, CH_3_	28.8, CH_3_

**Table 5:** Biological activities (IC_50_ µM) of **1**–**8**.

	*D. immitis* mf ^A^	*H. contortus* L1–L3 ^B^	*S. aureus*	*E. faecalis*	*E. coli*	*C. albicans*	SW620	NCI
**1**	21	^–^	9.2	8.5	^–^	^–^	^–^	^–^
**2**	^–^	^–^	22.4	^–^	^–^	^–^	^–^	^–^
**3**	^–^	^–^	^–^	^–^	^–^	^–^	^–^	^–^
**4**	^–^	^–^	^–^	^–^	^–^	^–^	^–^	^–^
**5**	10	^–^	19.1	^–^	^–^	^–^	^–^	^–^
**6**	11	^–^	^–^	^–^	^–^	^–^	^–^	^–^
**7**	^–^	^–^	^–^	^–^	^–^	^–^	^–^	^–^
**8**	^–^	^–^	^–^	^–^	^–^	^–^	^–^	^–^
gramicidin	0.10							
ivermectin		0.0005						
ampicillin			0.09		3.42			
amphotericin						0.17		
SDS							66.6	66.1

^A^ motility inhibition, ^B^ development inhibition, “^_^” inactive with IC_50_ ≥ 30 µM.

## Data Availability

The raw NMR data for goondicones A–H (**1**–**8**) have been deposited into the Natural Products Magnetic Resonance Database (NP-MRD; www.np-mrd.org, accessed on 28 November 2024) and can be found at NP0341988 (goondicone A), NP0341989 (goondicone B), NP0341990 (goondicone C), NP0341991 (goondicone D), NP0341992 (goondicone E), NP0341993 (goondicone F), NP0341994 (goondicone G), and NP0341995 (goondicone H).

## Supplementary Materials

The following supporting information can be downloaded at https://www.mdpi.com/article/10.3390/antibiotics13121222/s1, Bacterial taxonomy and phylogenetic analysis for *Streptomyces* sp. S4S-00185A06, together with NMR, HRESIMS, and biological assay data for **1**–**8**.
