# Supplementary material for: Goondicones A–H: Spiro-Isoindolinone Heartworm Anthelmintics from an Australian Pasture-Soil-Derived Streptomyces sp
Source: Antibiotics (Basel). 2024 Dec 17;13(12):1222. doi: 10.3390/antibiotics13121222 (PMC11727212; doi:10.3390/antibiotics13121222)
Supplement: Supplementary file 1 [file antibiotics-13-01222-s001.zip › antibiotics-3364447-supplementary.pdf]

Supporting information

Goondicones A–H: Spiro-isoindolinone heartworm anthelmintics from an Australian pasture soil-derived *Streptomyces* sp.

Jianying Han,[1] David F. Bruhn,[2] Cynthia T. Childs,[2] Yovany Moreno,[2] Angela A. Salim, [1] Taizong Wu,[3] and Robert J. Capon [1*]

[1] Institute for Molecular Bioscience, The University of Queensland, St Lucia, QLD 4072, Australia

[2] Boehringer Ingelheim Animal Health, USA Inc. 1730 Olympic Drive, Athens, GA 30601, USA

[3] Key Laboratory of Marine Genetic Resources, Third Institute of Oceanography, Ministry of Natural Resources, 184 Daxue Road, Xiamen 361005, China

* Correspondence: r.capon@uq.edu.au

Table of Contents

General experimental details. 5

Figure S1. S4S-00185A06 16S rRNA sequence. 6

Figure S2. BLAST search (closest match) of S4S-00185A06 16S rRNA sequence. 6

Figure S3. (Top) Phylogenetic tree of S4S-00185A06; (Bottom) image of S4S-00185A06 grown on an ISP2 agar plate. 7

Figure S4. (A) GNPS molecular network of ×110 prioritized extracts/isolates that inhibited D. immitis mf motility and/or H. contortus L1–L3 larval development (75% at 25 µg/mL); (B–C) molecular families unique to Streptomyces sp. S4S-00185A06 (green) with a UV-vis and MF combination thatb was unprecedented in the natural products literature. (C) UPLC-DAD (210 nm) chromatogram of the EtOAc extract of an ISP2 cultivation of *Streptomyces* sp. S4S-00185A06, highlighting metabolites associated with the GNPS molecular families (see B). 8

Table S1. Composition of media used for cultivation profiling (MATRIX) 8

Figure S5. S4S-00185A06 MATRIX cultivations. (A) solid, (B) broth static and (C) broth shaking. 10

Figure S6. UPLC-DAD (210 nm) chromatograms of S4S-00185A06 extracts from selected MATRIX media: (A) SDA; (B) GYA; (C) YES; (D) CGA; (E) IMA; (F) YEME; (G) M2; (H) M1; (I) 333; (J) ISP2; (K) D400; and under different conditions: (i) agar; (ii) static broth; (iii) shaken broth; (iv) media blank; * internal standard peak. 10

Spectroscopic characterisation goondicone A (1) 11

Table S2. 1D and 2D NMR (DMSO-d_6) data for goondicone A (**1**) 11

Figure S7. ^{1}H NMR (DMSO-d_6) and UV-vis (inset) spectra of goondicone A (**1**). 12

Figure S8. ^{13}C NMR (DMSO-d_6) spectrum of goondicone A (**1**) 12

Figure S9. HSQC NMR (DMSO-d_6) spectrum of goondicone A (**1**). 13

Figure S10. HMBC NMR (DMSO-d_6) spectrum of goondicone A (**1**) 13

Figure S11. ROESY NMR (DMSO-d_6) spectrum of goondicone A (**1**). 14

Figure S12. HRMS spectrum and measurement for goondicone A (**1**). 15

Spectroscopic characterisation goondicone B (2). 16

Table S3. 1D and 2D NMR (DMSO-d_6) data for goondicone B (**2**) 16

Figure S13. ^{1}H NMR (DMSO-d_6) and UV-vis (inset) spectra of goondicone B (**2**). 17

Figure S14. 13C NMR (DMSO-d6) spectrum of goondicone B (2). 17

Figure S15. HSQC NMR (DMSO-d_6) spectrum of goondicone B (**2**). 18

Figure S16. HMBC NMR (DMSO-d6) spectrum of goondicone B (2). 18

Figure S17. ROESY NMR (DMSO-d_6) spectrum of goondicone B (**2**). 19

Figure S18. HRMS spectrum and measurement for goondicone B (**2**). 20

Figure S19. Energy minimized conformations of 14*S*,9*R*-**1** (top) and 14*S*,9*S*-**2** (bottom) generated by Chem 3D, highlighting distances between H_3-10 to 11-OMe. 21

Spectroscopic characterisation goondicone C (3) 22

Table S4. 1D and 2D NMR (DMSO-d_6) data for goondicone C (**3**) 22
Figure S20. ^{1}H NMR (DMSO-d6) and UV-vis (inset) spectra of goondicone C (**3**). 23
Figure S21. 13C NMR (DMSO-d6) spectrum of goondicone C (**3**). 23
Figure S22. HSQC NMR (DMSO-d_6) spectrum of goondicone C (**3**). 24
Figure S23. HMBC NMR (DMSO-d6) spectrum of goondicone C (**3**). 24
Figure S24. ROESY NMR (DMSO-d_6) spectrum of goondicone C (**3**). 25
Figure S25. HRMS spectrum and measurement for goondicone C (**3**). 26
Spectroscopic characterisation goondicone D (4) 27
Table S5. 1D and 2D NMR (DMSO-d_6) data for goondicone D (**4**) 27
Figure S26. ^{1}H NMR (DMSO-d_6) and UV-vis (inset) spectra of goondicone D (**4**). 28
Figure S27. ^{13}C NMR (DMSO-d_6) spectrum of goondicone D (**4**). 28
Figure S28. HSQC NMR (DMSO-d_6) spectrum of goondicone D (**4**). 29
Figure S29. HMBC NMR (DMSO-d_6) spectrum of goondicone D (**4**). 29
Figure S30. ROESY NMR (DMSO-d_6) spectrum of goondicone D (**4**). 30
Figure S31. HRMS spectrum and measurement for goondicone D (**4**). 31
Spectroscopic characterisation goondicone E (5) 32
Table S6. 1D and 2D NMR (DMSO-d_6) data for goondicone E (**5**) 32
Figure S32. ^{1}H NMR (DMSO-d_6) and UV-vis (inset) spectra of goondicone E (**5**) 33
Figure S33. ^{13}C NMR (DMSO-d_6) spectrum of goondicone E (**5**) 33
Figure S34. HSQC NMR (DMSO-d_6) spectrum of goondicone E (**5**) 34
Figure S35. HMBC NMR (DMSO-d_6) spectrum of goondicone E (**5**) 35
Figure S36. ROESY NMR (DMSO-d_6) spectrum of goondicone E (**5**). 35
Figure S37. HRMS spectrum and measurement for goondicone E (**5**) 36
Spectroscopic characterisation goondicone F (6) 37
Table S7. 1D and 2D NMR (DMSO-d_6) data for goondicone F (**6**). 37
Figure S38. ^{1}H NMR (DMSO-d_6) and UV-vis (inset) spectra of goondicone F (**6**). 38
Figure S39. ^{13}C NMR (DMSO-d_6) spectrum of goondicone F (**6**). 38
Figure S40. HSQC NMR (DMSO-d_6) spectrum of goondicone F (**6**). 39
Figure S41. HMBC NMR (DMSO-d_6) spectrum of goondicone F (**6**). 40
Figure S42. ROESY NMR (DMSO-d_6) spectrum of goondicone F (**6**). 40
Figure S43. HRMS spectrum and measurement for goondicone F (**6**). 41
Spectroscopic characterisation goondicone G (7) 42
Table S8. 1D and 2D NMR (DMSO-d_6) data for goondicone G (**7**) 42
Figure S44. ^{1}H NMR (DMSO-d_6) and UV-vis (inset) spectra of goondicone G (**7**) 43
Figure S45. ^{13}C NMR (DMSO-d_6) spectrum of goondicone G (**7**) 43
Figure S46. HSQC NMR (DMSO-d_6) spectrum of goondicone G (**7**). 44
Figure S47. HMBC NMR (DMSO-d_6) spectrum of goondicone G (**7**). 45

Figure S48. ROESY NMR (DMSO-d_6) spectrum of goondicone G (**7**). 45

Figure S49. HRMS spectrum and measurement for goondicone G (**7**). 46

Spectroscopic characterisation goondicone H (8) 47

Table S9. 1D and 2D NMR (DMSO-d_6) data for goondicone H (**8**) 47

Figure S50. ^{1}H NMR (DMSO-d_6) and UV-vis (inset) spectra of goondicone H (**8**). 48

Figure S51. ^{13}C NMR (DMSO-d_6) spectrum of goondicone H (**8**). 48

Figure S52. HSQC NMR (DMSO-d_6) spectrum of goondicone H (**8**). 49

Figure S53. HMBC NMR (DMSO-d_6) spectrum of goondicone H (**8**). 49

Figure S54. ROESY NMR (DMSO-d_6) spectrum of goondicone H (**8**). 50

Figure S55. HRMS spectrum and measurement for goondicone H (**8**). 51

Figure S56. Single ion extraction of S4S-00185A06 fresh extract to show the presence of **1**–**8**. 52

Figure S57. UPLC-DAD (254 nm) chromatogram of (A) **5** treated with MeCN (0.01% TFA) overnight at 40 °C; and (B) heat in MeOH exposed to air overnight at 40 °C and authentic standard of **5** (C). 52

Figure S58. The experimental ECD spectra for **1**–**4**. 53

Figure S59. The experimental ECD spectra for **5**–**8**. 53

Antibacterial and Antifungal Assays 53

Figure S60. Antimicrobial activity of compounds **1**–**8** 54

Cytotoxicity Assay 54

Figure S61. Cytotoxicity of compounds **1**–**8** on SW620 and NCI-H460. 55

Table S10. Gibbs free energy and equilibrium populations of low-energy conformers of 14*R*,9*S*-**1A** in ECD calculations 55

Table S11. Gibbs free energy and equilibrium populations of low-energy conformers of 14*R*,9*R*-**2A** in ECD calculations 56

Table S12. Gibbs free energy and equilibrium populations of low-energy conformers of 14*S*,9*S*,8*R*-**5A** in ECD calculations 56

Table S13. Gibbs free energy and equilibrium populations of low-energy conformers of 14*S*,9*S*,8*S*-**5B** in ECD calculations 57

Table S14. Gibbs free energy and equilibrium populations of low-energy conformers of 14*S*,9*S*,8*R*-**6A** in ECD calculations 57

Table S15. Gibbs free energy and equilibrium populations of low-energy conformers of 14*S*,9*R*,8*S*-**6** in ECD calculations 58

General experimental details.

Chiroptical measurements ([a]D) were obtained on a JASCO P-1010 polarimeter in a 100 × 2 mm cell at specified temperatures. Nuclear magnetic resonance (NMR) spectra were acquired on a Bruker Avance 600 MHz spectrometer with a 5 mm PASEL ^{1}H/D-^{13}C Z-Gradient probe. In all cases, spectra were acquired at 25 °C in solvents as specified with referencing to residual solvent ^{1}H or ^{13}C NMR resonances (DMSO-d_6: d_H 2.50 and d_C 39.5). High-resolution ESIMS spectra were obtained on a Bruker micrOTOF mass spectrometer by direct injection in MeOH at 3 µL/min using sodium formate clusters as an internal calibrant. High-performance liquid chromatography-diode array-mass spectrometry (HPLC-DAD-MS) data were acquired on an Agilent 1260 series separation module equipped with an Agilent G6125B series LC/MSD mass detector and diode array detector (Poroshell 120 SB-C_8 2.7 mm, 150 × 3.0 mm column, gradient elution at 0.8 mL/min over 6.5 min from 90% H_2O/MeCN to 100% MeCN (with constant 0.05% formic acid/MeCN modifier). Semi-preparative HPLCs were performed using Agilent 1100 series HPLC instruments with corresponding detectors, fraction collectors and software inclusively. UPLC chromatograms were obtained on Agilent 1290 infinity UPLC system equipped with diode array multiple wavelength detector (Zorbax SB-C_8 RRHD 1.8 µm, 50 × 2.1 mm column, gradient elution at 0.417 mL/min over 2.50 min from 90% H_2O/MeCN to 100% MeCN with a constant 0.01% TFA/MeCN modifier). UPLC-QTOF analysis was performed on UPLC-QTOF instrument comprising an Agilent 1290 Infinity II UPLC (Zorbax SB-C_8 RRHD 1.8 µm, 50 × 2.1 mm column, gradient elution at 0.417 mL/min over 2.50 min from 90% H_2O/MeCN to 100% MeCN with a constant 0.1% formic acid/MeCN modifier) coupled to an Agilent 6545 Q-TOF. MS/MS analysis was performed on the same instrument for ions detected in the full scan at an intensity above 1000 counts at 10 scans/s, with an isolation width of 4 ~*m/z* using a fixed collision energy and a maximum of 3 selected precursors per cycle. Chemicals were purchased from Sigma-Aldrich or Merck unless otherwise specified. Analytical-grade solvents were used for solvent extractions. Chromatography solvents were of HPLC grade and supplied by Merck and filtered/degassed through 0.45 µm polytetrafluoroethylene (PTFE) membrane prior to use. Deuterated solvents were purchased from Cambridge Isotopes. Microorganisms were manipulated under sterile conditions using a Laftech class II biological safety cabinet and incubated in either MMM Friocell incubators (Lomb Scientific) or an Innova 42R incubator shaker (John Morris).

ATGCAAGTCGTAACAAGGTAGCCTTCGGGGTGGATTAGTGGCGAACGGGTGAGTAACACGTGG
GCAATCTGCCCTGCACTCTGGGACAAGCCCTGGAAACGGGGTCTAATACCGGATATGACCTTTGA
AGGCATCTTCAAAGGTGGAAAGCTCCGGCGGTGCAGGATGAGCCCGCGGCCTATCAGCTTGTTGG
TGGGGTGATGGCCTACCAAGGCGACGACGGGTAGCCGGCCTGAGAGGGCGACCGGCCACACTGG
GACTGAGACACGGCCCAGACTCCTACGGGAGGCAGCAGTGGGGAATATTGCACAATGGGCGAAA
GCCTGATGCAGCGACGCCGCGTGAGGGATGACGGCCTTCGGGTTGTAAACCTCTTTCAGCAGGGA
AGAAGCGAAAGTGACGGTACCTGCAGAAGAAGCGCCGGCTAACTACGTGCCAGCAGCCGCGGTA
ATACGTAGGGCGCAAGCGTTGTCCGGAATTATTGGGCGTAAAGAGCTCGTAGGCGGCTTGTCACG
TCGGATGTGAAAGCCCGGGGCTTAACCCCGGGTCTGCATTCGATACGGGCAGGCTAGAGTTCGGT
AGGGGAGATCGGAATTCCTGGTGTAGCGGTGAAATGCGCAGATATCAGGAGGAACACCGGTGGC
GAAGGCGGATCTCTGGGCCGATACTGACGCTGAGGAGCGAAAGCGTGGGGAGCGAACAGGATTA
GATACCCTGGTAGTCCACGCCGTAAACGTTGGGAACTAGGTGTGGGCGACATTCCACGTCGTCCGT
GCCGCAGCTAACGCATTAAGTTCCCCGCCTGGGGAGTACGGCCGCAAGGCTAAAACTCAAAGGAA
TTGACGGGGGCCCGCACAAGCAGCGGAGCATGTGGCTTAATTCGACGCAACGCGAAGAACCTTAC
CAAGGCTTGACATACACCGGAAACGGCCAGAGATGGTCGCCCCCTTGTGGTCGGTGTACAGGTGG
TGCATGGCTGTCGTCAGCTCGTGTCGTGAGATGTTGGGTTAAGTCCCGCAACGAGCGCAACCCTTG
TCCTGTGTTGCCGCATGCCCTTCGGGGTGATGGGGACTCACAGGAAACTGCCGGGTCACTCGGAA
GAAGGTGGGGACGACGTCAAGTCTTCGCCCCTTATGCTGGGCTGCAACGTGCTAATGGCCGGTAC
ATGAACTG

Figure S1. S4S-00185A06 16S rRNA sequence.

Description	Scientific Name	Max Score	Total Score	Query Cover	E value	Per. Ident	Acc. Len	Accession
Streptomyces caeruleus partial 16S rRNA gene, strain OS3-4	Actinoalloteichus...	2065	2065	100%	0.0	98.31%	1431	FN178412.1
Streptomyces sp. MK597-CF12 gene for 16S ribosomal RNA, partial sequence	Streptomyces sp...	2021	2021	98%	0.0	98.20%	1403	AB797217.1
Streptomyces cinnamoneus gene for 16S rRNA, partial sequence, strain: NBRC 15928	Streptomyces ci...	2021	2021	98%	0.0	98.19%	1409	AB184718.1
Streptomyces sp. KPS-F003 16S ribosomal RNA gene, partial sequence	Streptomyces sp...	1984	1984	96%	0.0	98.16%	1397	KM288434.1
Streptomyces sp. LLH-Irr-13 16S ribosomal RNA gene, partial sequence	Streptomyces sp...	2021	2021	98%	0.0	98.12%	1400	KM287555.1
Streptomyces roseoverticillatus strain NRRL B-1993 16S ribosomal RNA, partial sequence	Streptomyces ro...	2043	2043	100%	0.0	97.97%	1504	NR_118009.1
Streptomyces roseoverticillatus gene for 16S rRNA, partial sequence, strain: NBRC 3726	Streptomyces ro...	2039	2039	100%	0.0	97.89%	1472	AB184794.1
Streptomyces salmonis strain 261 16S ribosomal RNA gene, partial sequence	Streptomyces sa...	1999	1999	98%	0.0	97.85%	1415	MG574820.1
Streptomyces sp. FXJ1.822 16S ribosomal RNA gene, complete sequence	Streptomyces sp...	1988	1988	97%	0.0	97.84%	1336	KJ152056.1
Streptomyces sp. CMU-NKS-79 16S ribosomal RNA gene, partial sequence	Streptomyces sp...	1986	1986	97%	0.0	97.84%	1328	KF746338.1
Streptomyces roseoverticillatus strain NBRC 12817 16S ribosomal RNA, partial sequence	Streptomyces ro...	2041	2041	100%	0.0	97.81%	1466	NR_112288.1
Streptomyces salmonis strain NBRC 15865 16S ribosomal RNA, partial sequence	Streptomyces sa...	2032	2032	100%	0.0	97.81%	1458	NR_112478.1
Streptomyces roseoverticillatus gene for 16S rRNA, partial sequence, strain: NBRC 14694	Streptomyces ro...	2032	2032	100%	0.0	97.81%	1469	AB184614.1
Streptomyces cinnamoneus gene for 16S rRNA, partial sequence, strain: NBRC 15926	Streptomyces ci...	2032	2032	100%	0.0	97.81%	1472	AB184716.1
Streptomyces cinnamoneus subsp. sparsus strain JCM 10107 16S ribosomal RNA gene, partial sequence	Streptomyces ci...	2032	2032	100%	0.0	97.81%	1423	AY999754.1
Streptomyces sp. LLH-Irr-26 16S ribosomal RNA gene, partial sequence	Streptomyces sp...	2030	2030	99%	0.0	97.81%	1409	KM287554.1
Streptomyces sp. strain ABV 45 16S ribosomal RNA gene, partial sequence	Streptomyces sp.	2028	2028	99%	0.0	97.80%	1231	MT435540.1
Streptomyces sp. LLH-Irr-33 16S ribosomal RNA gene, partial sequence	Streptomyces sp...	2021	2021	99%	0.0	97.80%	1406	KM287553.1
Streptomyces sp. LLH-Irr-32 16S ribosomal RNA gene, partial sequence	Streptomyces sp...	2021	2021	99%	0.0	97.80%	1404	KM287552.1
Streptomyces sp. SDSLU14 16S ribosomal RNA gene, partial sequence	Streptomyces sp...	1993	1993	98%	0.0	97.76%	1418	KT593926.1
Streptomyces parvisporogenes strain S2A-04 16S ribosomal RNA gene, partial sequence	Streptomyces pa...	1986	1986	97%	0.0	97.76%	1339	MG719969.1
Streptomyces sp. TS3RO partial 16S rRNA gene, isolate TS3RO	Streptomyces sp...	2026	2026	100%	0.0	97.72%	1476	FR854235.1
Streptomyces cinnamoneus gene for 16S rRNA, partial sequence, strain: NBRC 15927	Streptomyces ci...	2028	2028	100%	0.0	97.72%	1469	AB184717.1
Streptomyces sp. strain ABV 38 16S ribosomal RNA gene, partial sequence	Streptomyces sp.	1995	1995	98%	0.0	97.69%	1404	MT435538.1
Streptomyces luteoverticillatus strain NBRC 12887 16S ribosomal RNA, partial sequence	Streptomyces lut...	1988	1988	98%	0.0	97.68%	1372	NR_118282.1
Streptomyces ehimensis strain NBRC 12858 16S ribosomal RNA, partial sequence	Streptomyces eh...	1988	1988	98%	0.0	97.68%	1371	NR_118281.1
Streptomyces luteoverticillatus strain CGMCC 4.1974 16S ribosomal RNA gene, partial sequence	Streptomyces lut...	1988	1988	98%	0.0	97.68%	1380	HQ244460.1
Streptomyces ehimensis strain CGMCC 4.1668 16S ribosomal RNA gene, partial sequence	Streptomyces eh...	1988	1988	98%	0.0	97.68%	1373	HQ244451.1
Streptomyces sp. strain KP8 16S ribosomal RNA gene, partial sequence	Streptomyces sp.	2021	2021	100%	0.0	97.64%	1484	OL655273.1
Streptomyces roseoverticillatus strain 63 16S ribosomal RNA gene, partial sequence	Streptomyces ro...	2021	2021	100%	0.0	97.64%	1461	MT832024.1
Streptomyces thioluteus strain JSZZ28 16S ribosomal RNA gene, partial sequence	Streptomyces thi...	2021	2021	100%	0.0	97.64%	1421	HQ853022.1
Streptomyces cinnamoneus subsp. lanosus strain JCM 10106 16S ribosomal RNA gene, partial sequence	Streptomyces ci...	2019	2019	100%	0.0	97.64%	1418	AY999747.1
Streptomyces roseoverticillatus 16S rRNA gene, type strain LMG 20255	Streptomyces ro...	2021	2021	100%	0.0	97.56%	1486	AJ781361.1

Figure S2. BLAST search (closest match) of S4S-00185A06 16S rRNA sequence.

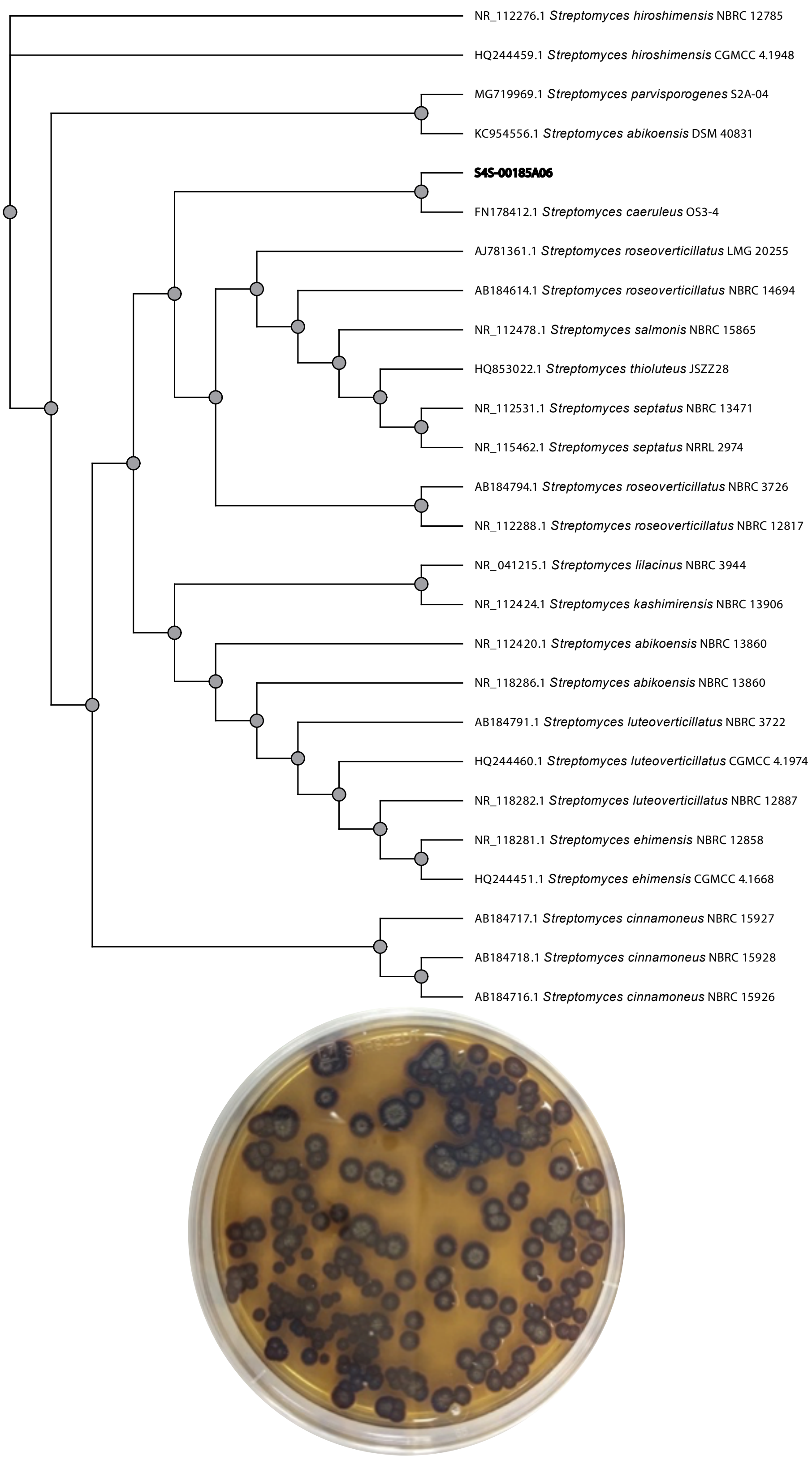

Figure S3. (Top) Phylogenetic tree of S4S-00185A06; (Bottom) image of S4S-00185A06 grown on an ISP2 agar plate.

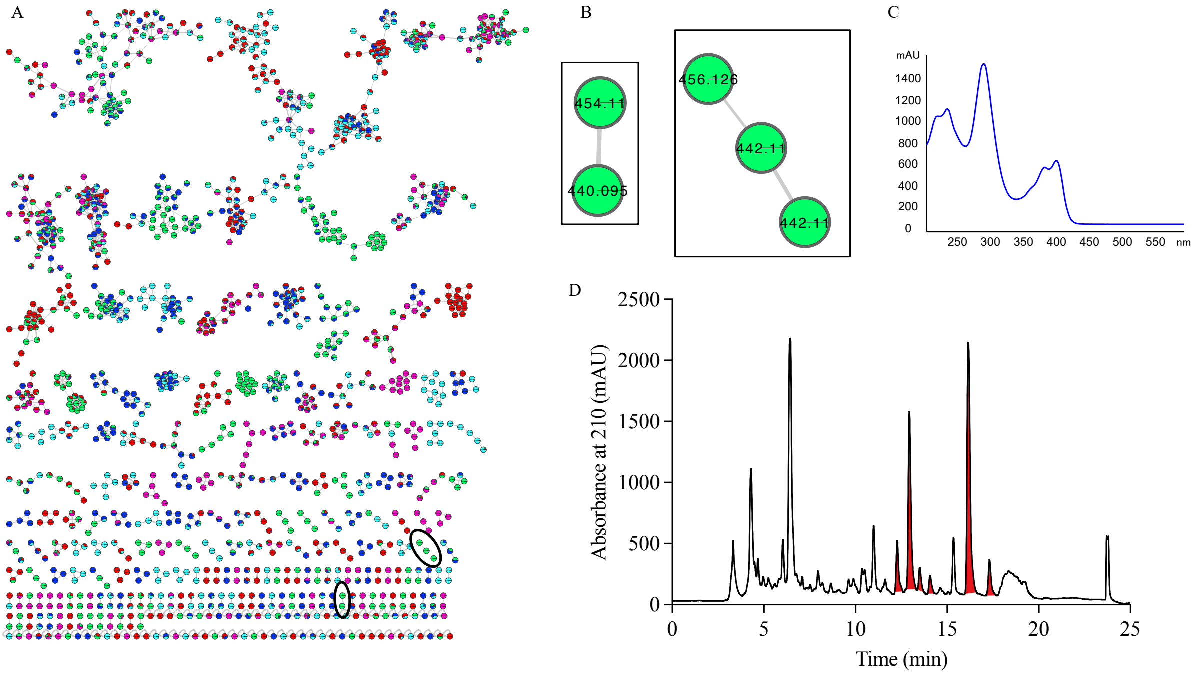

Figure S4. (A) GNPS molecular network of ×110 prioritized extracts/isolates that inhibited D. immitis mf motility and/or H. contortus L1–L3 larval development (75% at 25 µg/mL); (B–C) molecular families unique to Streptomyces sp. S4S-00185A06 (green) with a UV-vis and MF combination thatb was unprecedented in the natural products literature. (C) UPLC-DAD (210 nm) chromatogram of the EtOAc extract of an ISP2 cultivation of *Streptomyces* sp. S4S-00185A06, highlighting metabolites associated with the GNPS molecular families (see B).

Table S1. Composition of media used for cultivation profiling (MATRIX)

Medium	Composition (per litre)

**333**	glucose (5.0 g), peptone (3.0 g), soluble starch (10.0 g), yeast extract (3.0 g), $CaCO_3$ (2.0 g).
**D400**	glucose (10.0 g), malt extract (3.0 g), peptone (3.0 g), soluble starch (20.0 g), yeast extract (5.0 g), $CaCO_3$ (3.0 g).
**M1**	peptone (2.0 g), yeast extract (4.0 g), starch (10.0 g).
**M2**	mannitol (40.0 g), maltose (40.0 g), yeast extract (10.0 g), K_2HPO_4 (2.0 g), $MgSO_4.7H_2O$ (0.5 g), $FeSO_4.7H_2O$ (0.01 g)
**ISP2**	yeast extract (4.0 g), malt extract (10.0 g), glucose (4.0 g).
**Sabouraud Dextrose (SD)**	peptic digest of animal tissue (5.0 g), pancreatic digest of casein (5.0 g), dextrose (40.0 g).
**GY**	yeast extract (Difco) (4.0 g), malt extract (Difco) (10.0 g), glucose (country brewers) (4.0 g), $CaCO_3$ (Univar Ajax) (2.0 g), soluble starch (Difco) (20.0 g).
**CG**	glycerol (Chem-Supply) (30.0 g), Casein peptone (Amyl) (2.0 g), K_2HPO_4 (Chem-Supply) (1.0 g), NaCl (Chem-Supply) (1.0 g), $MgSO_4.7H_2O$ (AnalaR) (0.5 g), trace element solution* (5.0 mL).<br>***trace element solution**<br>$CaCl_2.2H_2O$ (3.0 g), $FeC_6O_7H_5$ (1.0 g), $MnSO_4$ (0.2 g), $ZnCl_2$ (0.1 g), $CuSO_4.5H_2O$ (0.025 g), $Na_2B_4O_7.10H_2O$ (0.02 g), $CoCl_2$ (0.004 g), $Na_2MoO_4.2H_2O$ (0.01 g), distilled H_2O (1000 mL) (Filter sterilize).
**IM**	yeast extract (Difco) (4.0 g), malt extract (Difco) (10.0 g), glucose (country brewers) (4.0 g), mannitol (Amyl) 40.0 g.
**YES**	sucrose (150 g), yeast extract (20 g), $MgSO_4.7H_2O$ (0.5 g), $ZnSO_4.7H_2O$ (0.01 g), $CuSO_4.5H_2O$ (0.005 g).
**Modified YEME**	Bacto peptone (Difco) (5.0 g), yeast extract (Difco) (3.0 g), Oxoin malt extract (3.0 g), glucose (10.0 g), sucrose (170.0 g).

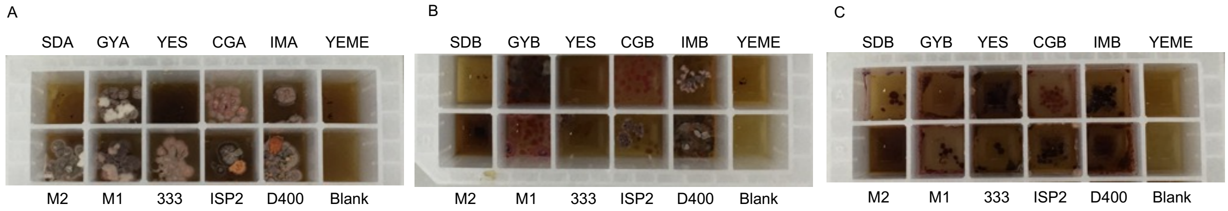

Figure S5. S4S-00185A06 MATRIX cultivations. (A) solid, (B) broth static and (C) broth shaking.

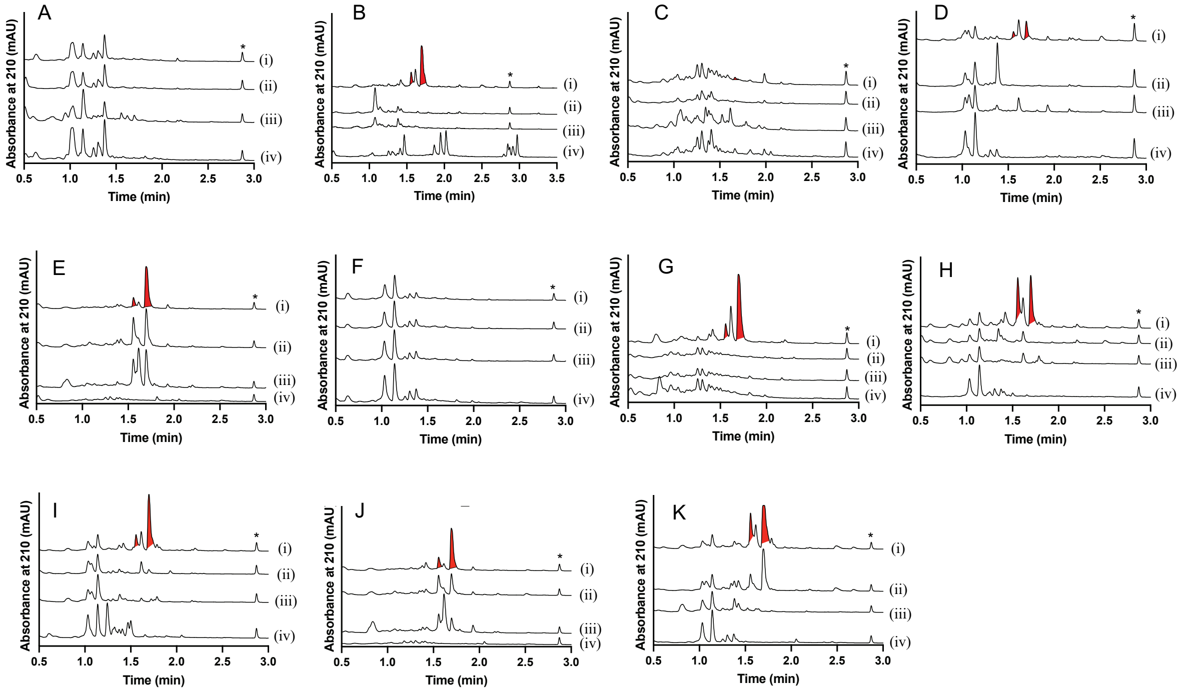

Figure S6. UPLC-DAD (210 nm) chromatograms of S4S-00185A06 extracts from selected MATRIX media: (A) SDA; (B) GYA; (C) YES; (D) CGA; (E) IMA; (F) YEME; (G) M2; (H) M1; (I) 333; (J) ISP2; (K) D400; and under different conditions: (i) agar; (ii) static broth; (iii) shaken broth; (iv) media blank; * internal standard peak.

Spectroscopic characterisation goondicone A (1)

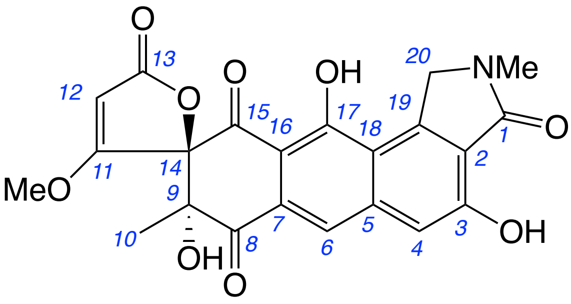

(**1**)

Table S2. 1D and 2D NMR (DMSO-d_6) data for goondicone A (**1**)

position	δ_C, type	δ_H, mult.	HMBC	ROESY
1	166.0, C	-	-	-
2	122.0, C	-	-	-
3	157.5, C	-	-	-
4	112.9, CH	7.52, s	2, 3, 6, 18	6
5	140.7, C	-	-	-
6	119.2, CH	8.07, s	4, 5, 8, 16, 18	4
7	129.5, C	-	-	-
8	194.8, C	-	-	-
9	78.3, C	-	-	-
10	22.4, CH_3	1.40, s	8, 9, 14	9-OH
11	177.8, C	-	-	-
12	89.9, CH	5.58, s	11, 13, 14	11-OMe
13	170.9, C	-	-	-
14	90.4, C	-	-	-
15	192.5, C	-	-	-
16	108.0, C	-	-	-
17	163.6, C	-	-	-
18	115.3, C	-	-	-
19	145.0, C	-	-	-
20	53.6, CH_2	4.97, s	1, 2, 18, 19, *N*-Me	*N*-Me
N-Me	28.8, CH_3	3.14, s	1, 20	20
11-OMe	61.1, CH_3	3.69, s	11	12
3-OH	-	10.84, s	2, 3, 4	
9-OH	-	6.49, s	8, 9, 10, 14	10
17-OH	-	13.38, s		

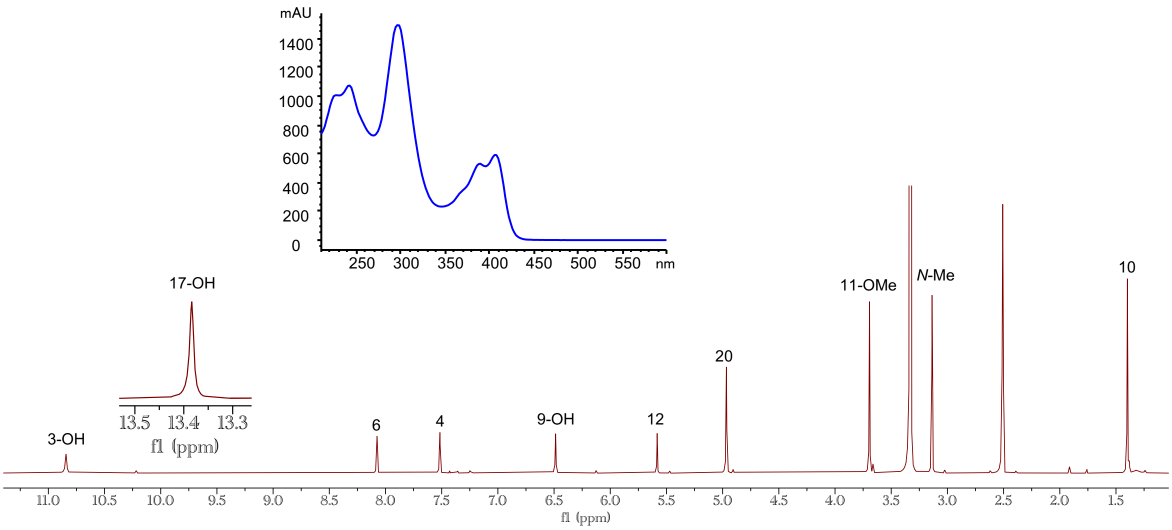

Figure S7. ^{1}H NMR (DMSO-d_6) and UV-vis (inset) spectra of goondicone A (**1**).

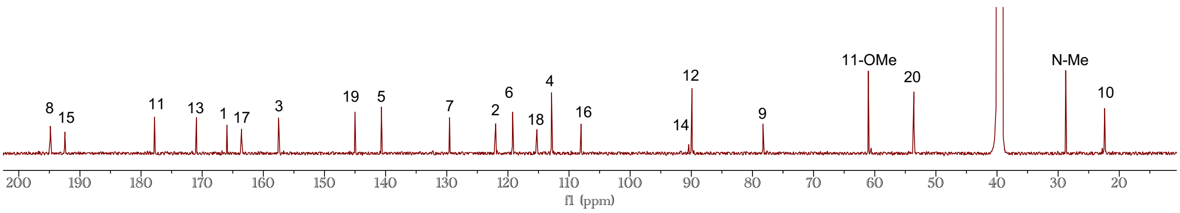

Figure S8. ^{13}C NMR (DMSO-d_6) spectrum of goondicone A (**1**)

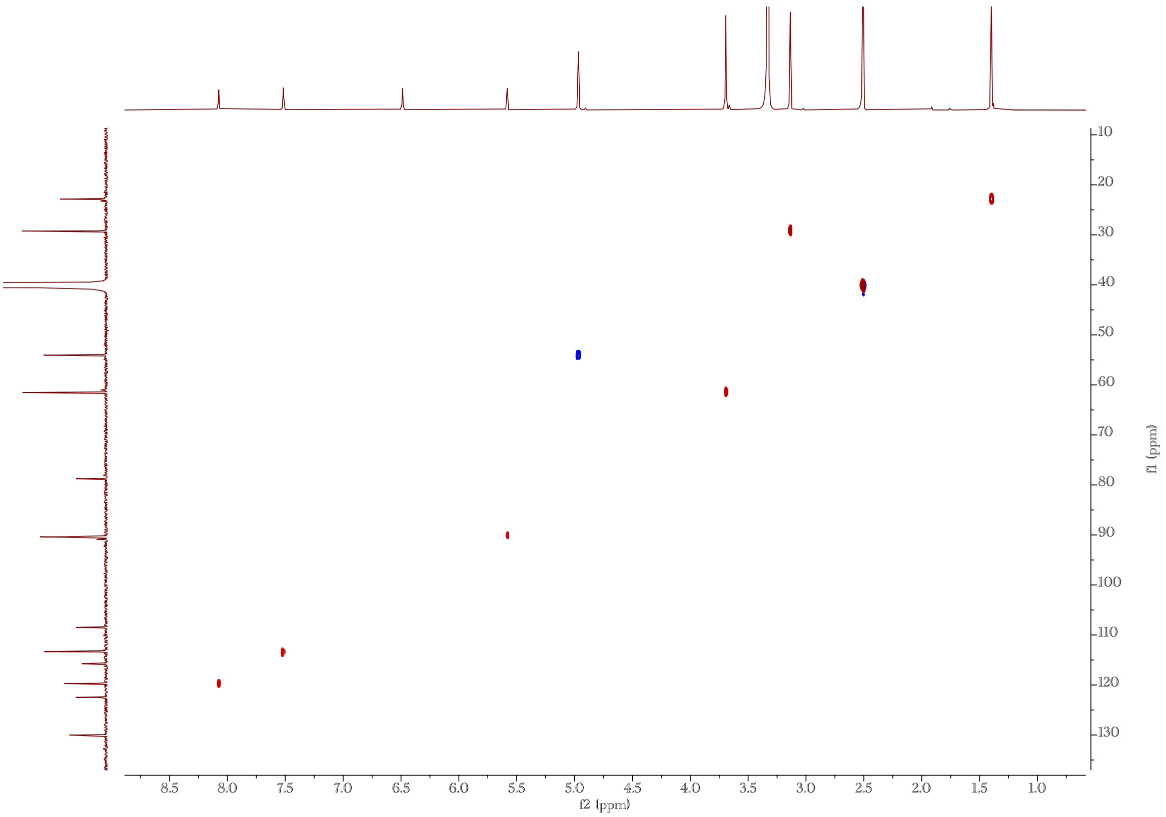

Figure S9. HSQC NMR (DMSO-d_6) spectrum of goondicone A (**1**)

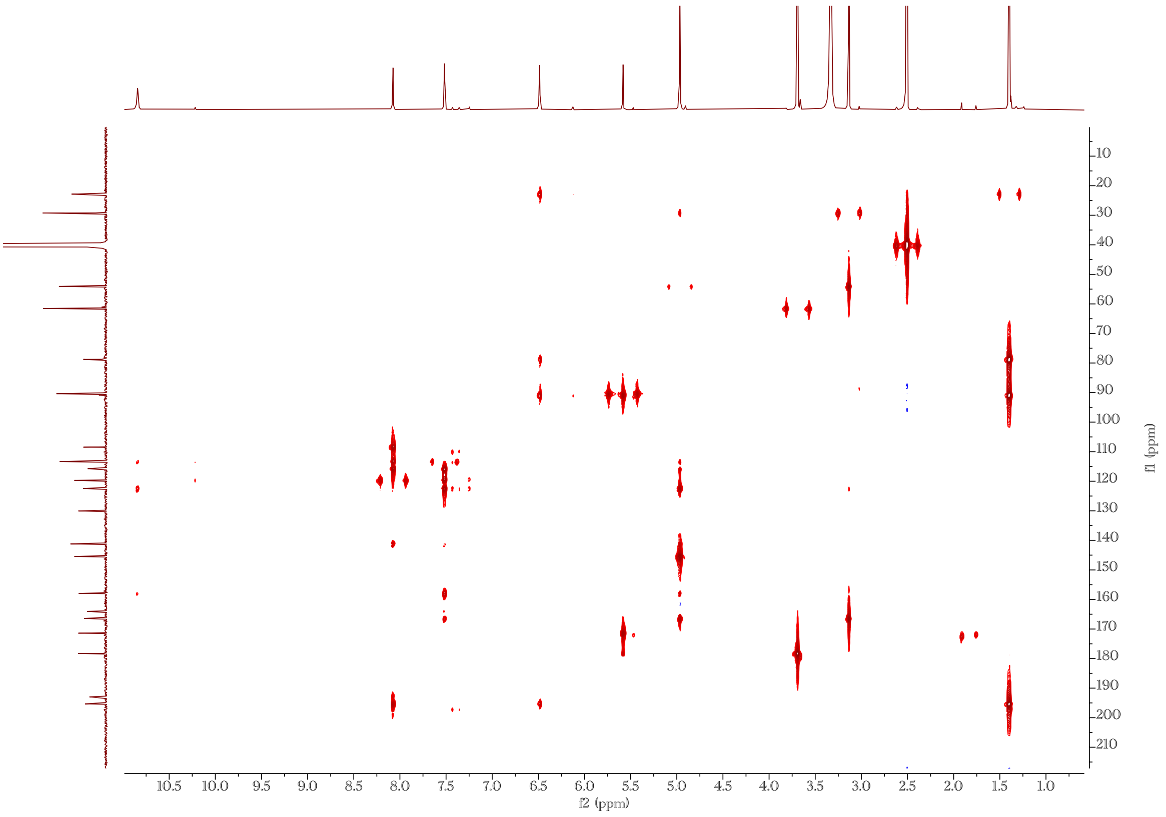

Figure S10. HMBC NMR (DMSO-d_6) spectrum of goondicone A (**1**)

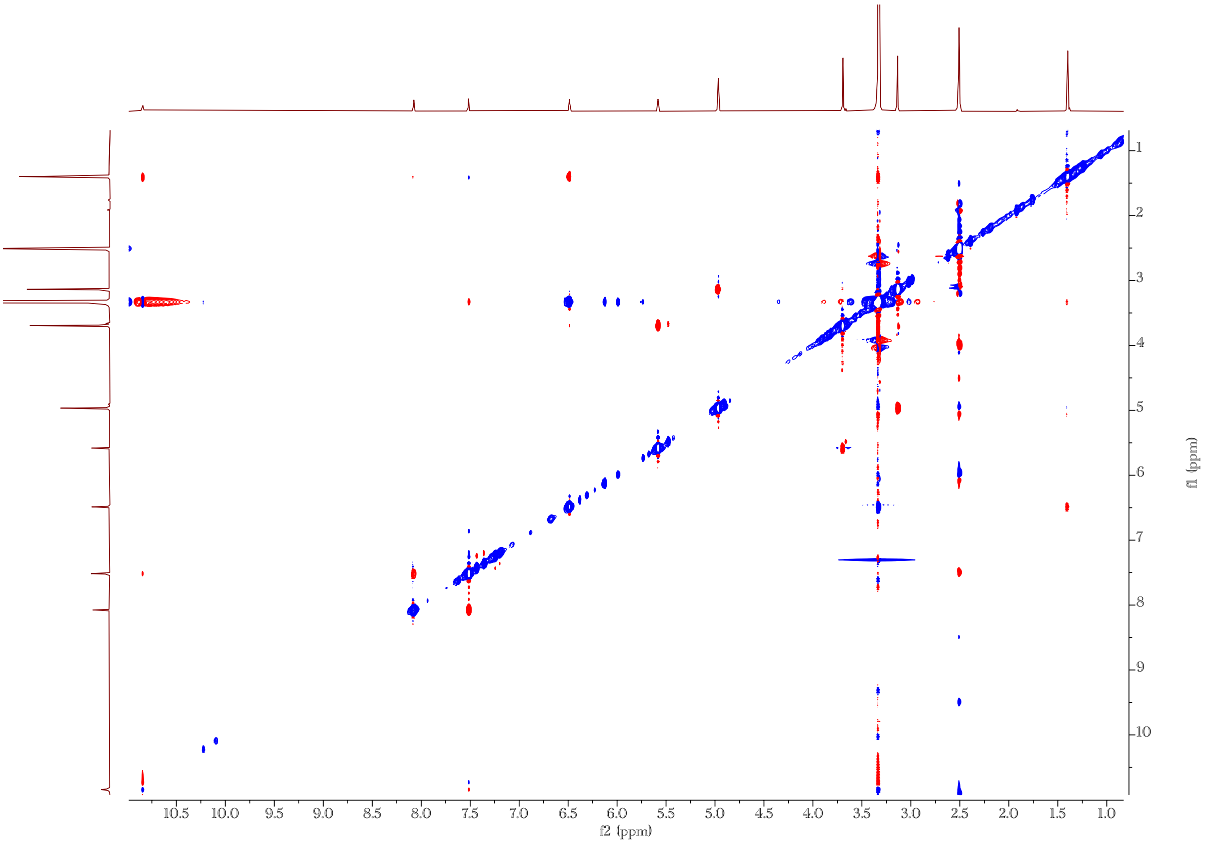

Figure S11. ROESY NMR (DMSO-d_6) spectrum of goondicone A (**1**).

Mass Spectrum Molecular Formula Report

Analysis Info

Analysis Name	D:\Data\Jianying\S4S-00185A06-P2.d	Acquisition Date	12/2/2021 3:37:40 PM	
Method	tune-medhigh_AP.m	Operator	a.salim	
Sample Name		Instrument / Ser#	micrOTOF	213750.00232
Comment				

Acquisition Parameter

Source Type	ESI	Ion Polarity	Positive	Set Nebulizer	0.5 Bar
Focus	Not active			Set Dry Heater	180 °C
Scan Begin	100 m/z	Set Capillary	4500 V	Set Dry Gas	5.0 l/min
Scan End	1500 m/z	Set End Plate Offset	-500 V	Set Divert Valve	Source

Generate Molecular Formula Parameter

Formula, min.		
Formula, max.		
Measured m/z	Tolerance	Charge
Check Valence	Minimum	Maximum
Nirogen Rule	Electron Configuration	
Filter H/C Ratio	Minimum	Maximum
Estimate Carbon		

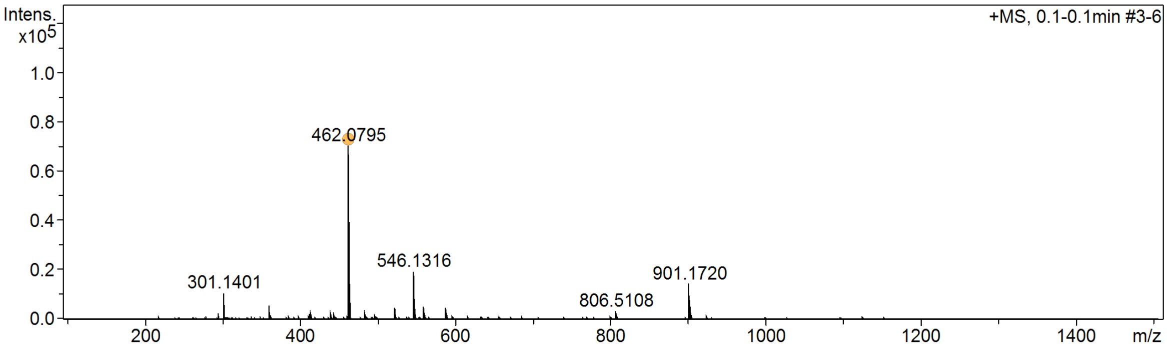

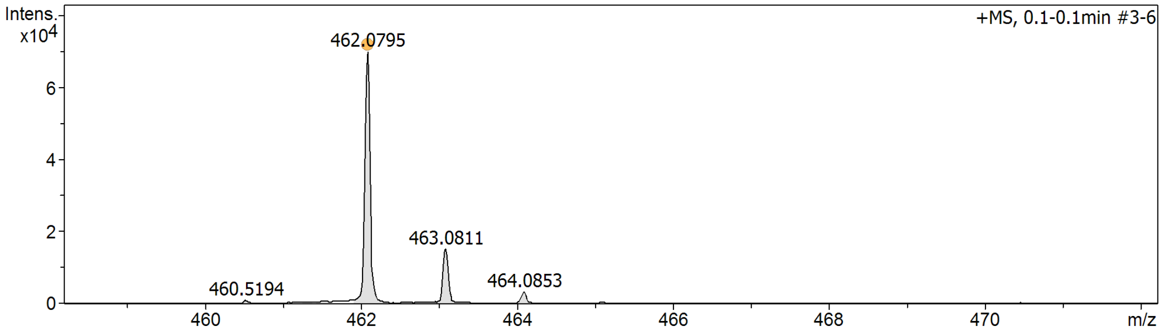

Meas. m/z	#	Ion Formula	m/z	err [ppm]	mSigma	# Sigma	Score	rdb	e⁻ Conf	N-Rule
462.0795	1	C22H17NNaO9	462.0796	0.2	15.4	1	100.00	14.5	even	ok

Figure S12. HRMS spectrum and measurement for goondicone A (**1**).

Spectroscopic characterisation goondicone B (2)

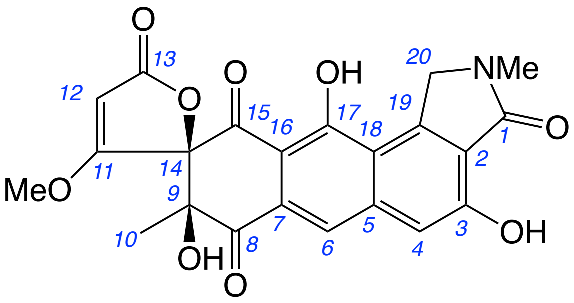

(**2**)

Table S3. 1D and 2D NMR (DMSO-d_6) data for goondicone B (**2**)

position	δ_C, type	δ_H, mult.	HMBC	ROESY
1	166.1, C	-	-	-
2	121.9, C	-	-	-
3	157.2, C	-	-	-
4	112.7, CH	7.50, s	2, 3, 6, 18	6, 3-OH
5	140.7, C	-	-	-
6	119.4, CH	8.05, s	4, 5, 8, 16, 18	4
7	130.5, C	-	-	-
8	191.4,[a] C	-	-	-
9	77.6, C	-	-	-
10	16.2,[b] CH_3	1.34, s	8, 9, 14	12, 9-OH, 11-OMe
11	177.2, C	-	-	-
12	90.4, CH	5.68, s	11, 13, 14	10, 11-OMe
13	170.8, C	-	-	-
14	90.0, C	-	-	-
15	192.8, C	-	-	-
16	108.1, C	-	-	-
17	162.8, C	-	-	-
18	115.2, C	-	-	-
19	144.8, C	-	-	-
20	53.6, CH_2	4.99, ABq (20.2)	1, 2, 19	*N*-Me
		4.95, ABq (20.2)	1, 2, 19	*N*-Me
N-Me	28.7, CH_3	3.14, s	1, 20	20
11-OMe	61.4, CH_3	3.76, s	11	12, 10
3-OH	-	10.77, s	2, 3, 4	4
9-OH	-	6.99, s		10
17-OH	-	13.23, s	16, 17, 18	20

[a] Detected from HMBC; [b] Detected from HSQC

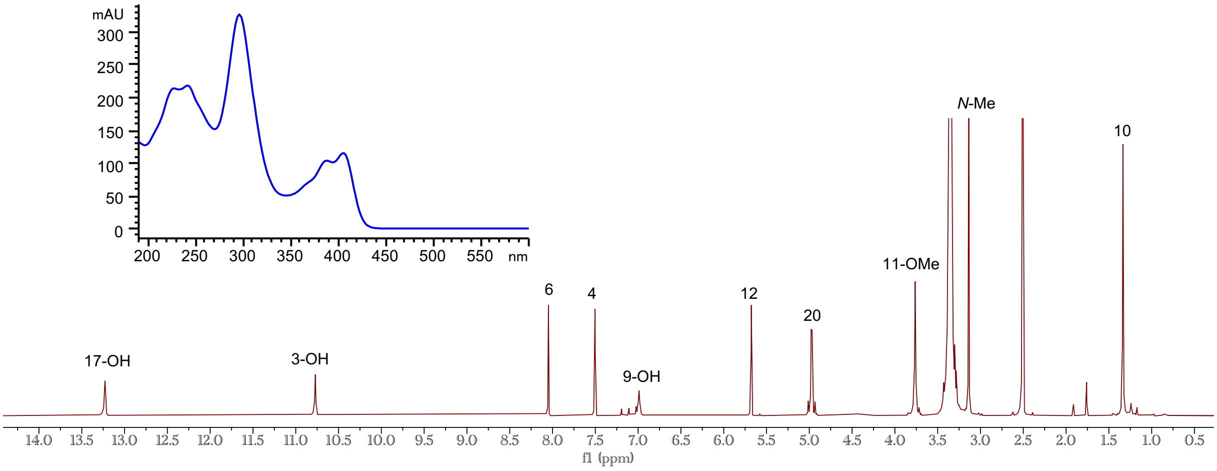

Figure S13. ^{1}H NMR (DMSO-d_6) and UV-vis (inset) spectra of goondicone B (**2**).

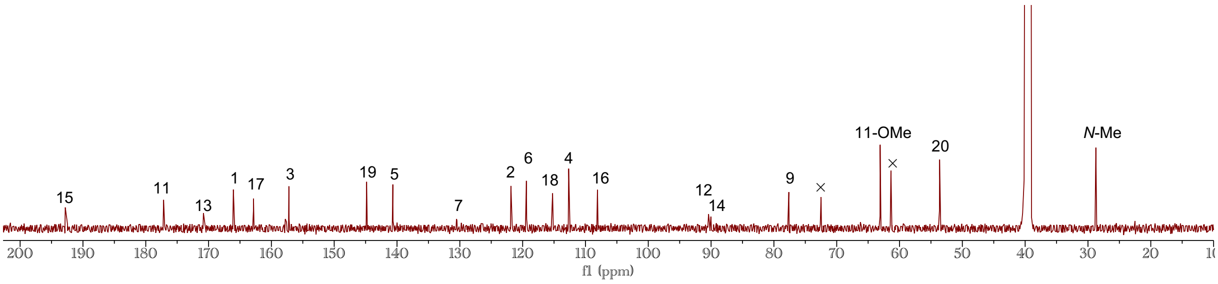

Figure S14. 13C NMR (DMSO-d6) spectrum of goondicone B (2).

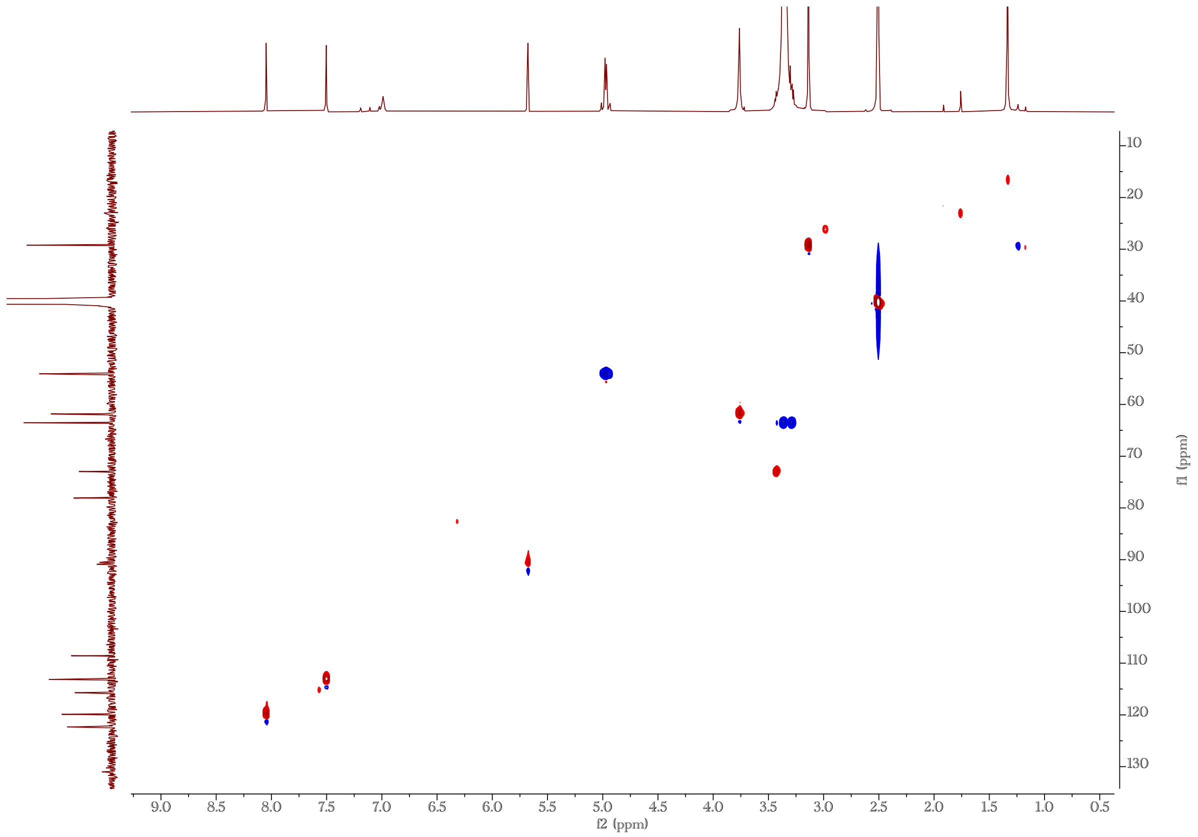

Figure S15. HSQC NMR (DMSO-*d*$_6$) spectrum of goondicone B (**2**).

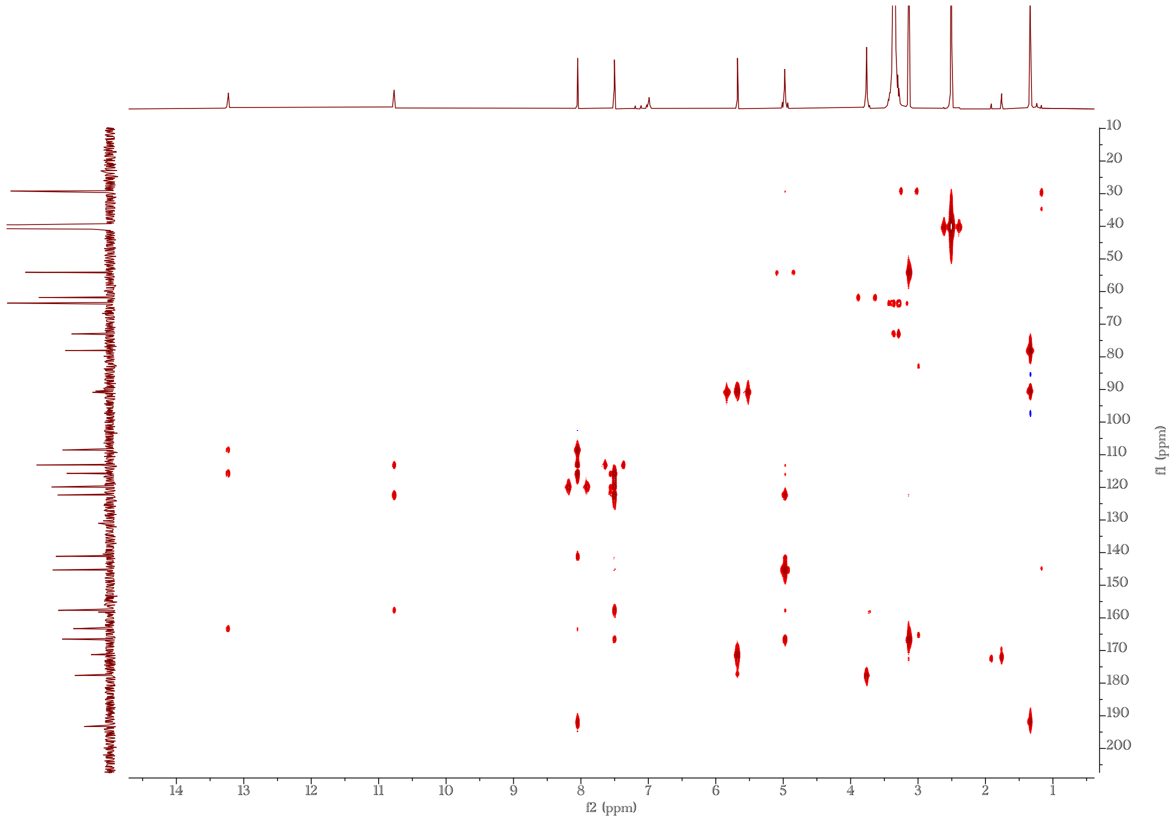

Figure S16. HMBC NMR (DMSO-d6) spectrum of goondicone B (2).

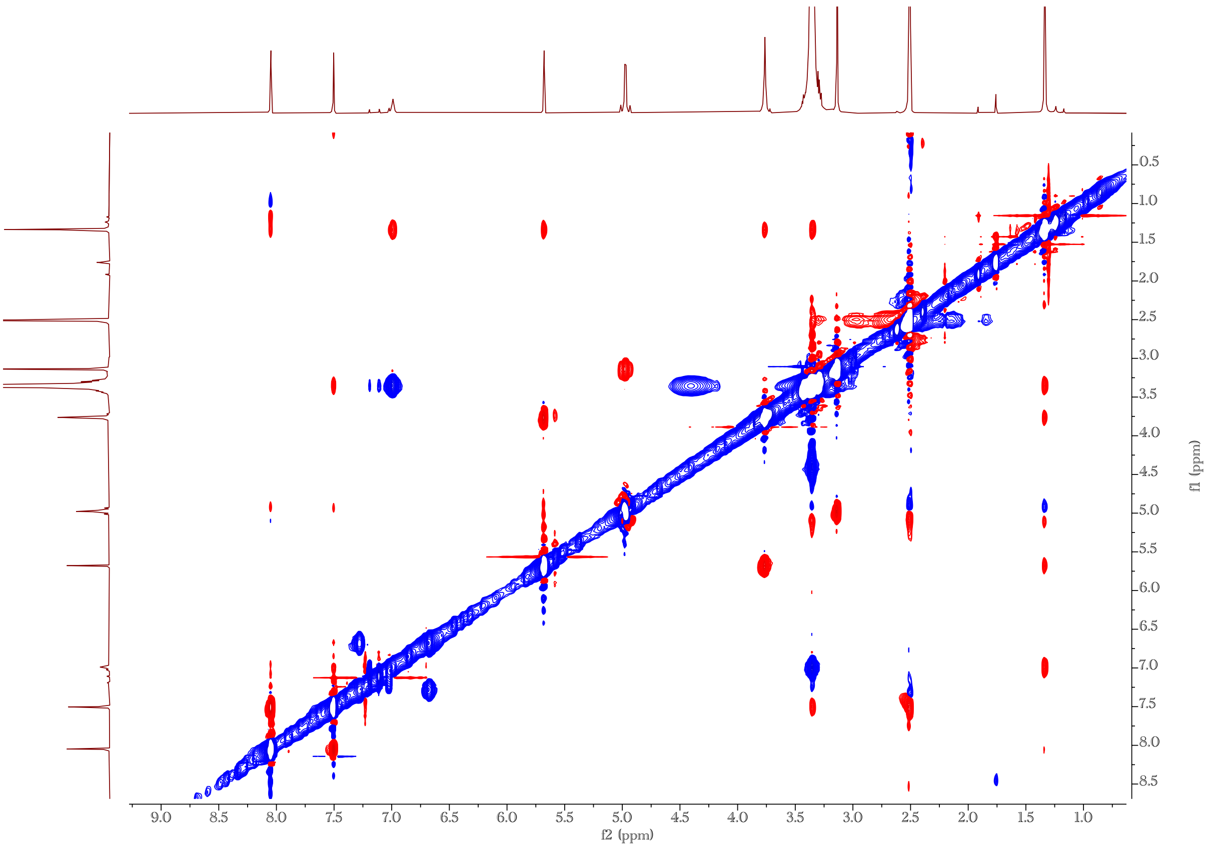

Figure S17. ROESY NMR (DMSO-d_6) spectrum of goondicone B (**2**).

Mass Spectrum Molecular Formula Report

Analysis Info

Analysis Name	D:\Data\Jianying\S4S-00185A06-L-F17-P2.d	Acquisition Date	2/15/2022 1:28:33 PM	
Method	tune-medhigh_AP.m	Operator	a.salim	
Sample Name	S4S-00185A06-L-F17-P2	Instrument / Ser#	micrOTOF	213750.00232
Comment				

Acquisition Parameter

Source Type	ESI	Ion Polarity	Positive	Set Nebulizer	0.5 Bar
Focus	Not active			Set Dry Heater	180 °C
Scan Begin	100 m/z	Set Capillary	4500 V	Set Dry Gas	5.0 l/min
Scan End	1500 m/z	Set End Plate Offset	-500 V	Set Divert Valve	Source

Generate Molecular Formula Parameter

Formula, min.
Formula, max.
Measured m/z — Tolerance — Charge
Check Valence — Minimum — Maximum
Nirogen Rule — Electron Configuration
Filter H/C Ratio — Minimum — Maximum
Estimate Carbon

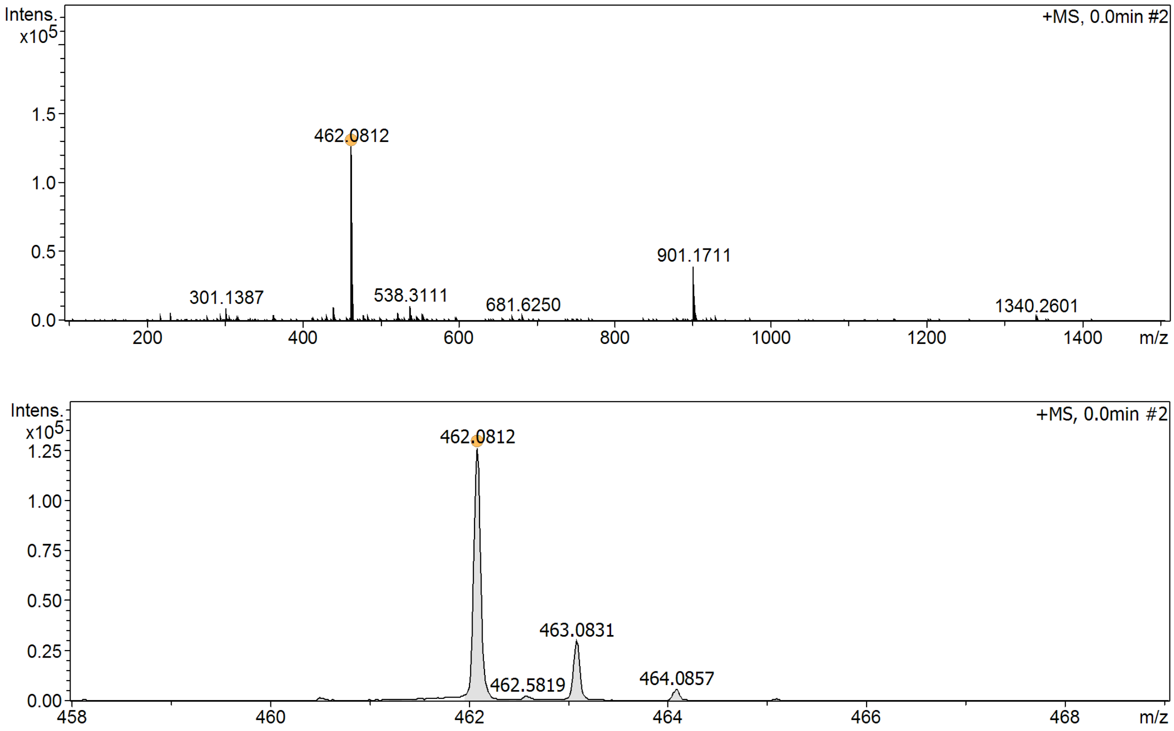

Meas. m/z	#	Ion Formula	m/z	err [ppm]	mSigma	# Sigma	Score	rdb	e⁻ Conf	N-Rule
462.0812	1	C22H17NNaO9	462.0796	3.6	5.3	1	57.94	14.5	even	ok
	2	C23H13N5NaO5	462.0809	-0.7	16.9	2	100.00	19.5	even	ok
	3	C24H9N9NaO	462.0822	-2.2	28.6	3	54.18	24.5	even	ok

Figure S18. HRMS spectrum and measurement for goondicone B (**2**).

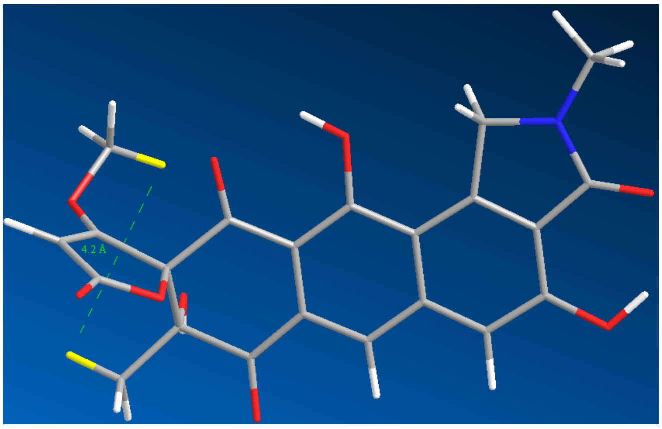

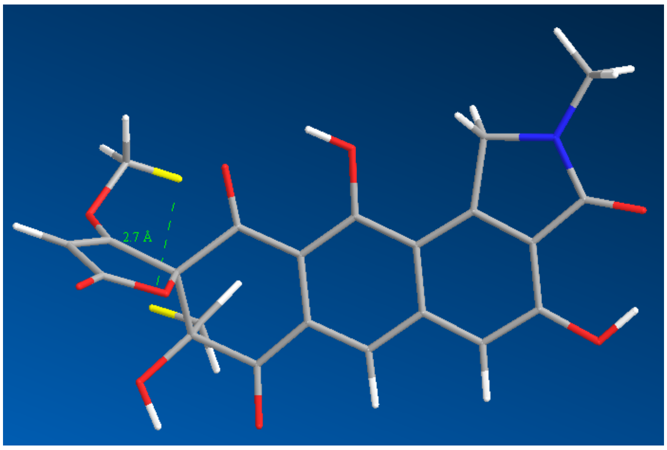

Figure S19. Energy minimized conformations of 14*S*,9*R*-**1** (top) and 14*S*,9*S*-**2** (bottom) generated by Chem 3D, highlighting distances between H_3-10 to 11-OMe.

Spectroscopic characterisation goondicone C (3)

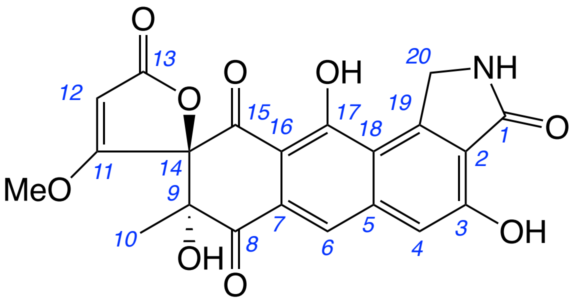

(**3**)

Table S4. 1D and 2D NMR (DMSO-d_6) data for goondicone C (**3**)

position	δ_C, type	δ_H, mult.	HMBC	ROESY
1	169.0, C	-	-	-
2	121.8, C	-	-	-
3	157.9, C	-	-	-
4	112.7, CH	7.52, s	2, 3, 6, 18	6, 3-OH
5	141.1, C	-	-	-
6	119.2, CH	8.08, s	4, 5, 8, 16, 18	4
7	129.6, C	-	-	-
8	194.9, C	-	-	-
9	78.3, C	-	-	-
10	22.4, CH_3	1.40, s	8, 9, 14	9-OH, 6
11	177.8, C	-	-	-
12	90.4, CH	5.58, s	11, 13, 14	11-OMe
13	171.0, C	-	-	-
14	90.4, C	-	-	-
15	192.5, C	-	-	-
16	108.0, C	-	-	-
17	163.6, C	-	-	-
18	115.7, C	-	-	-
19	147.3, C	-	-	-
20	47.4, CH_2	4.89, s	1, 2, 19	NH
11-OMe	61.1, CH_3	3.69, s	11	12
3-OH	-	10.73, s	2, 3, 4	4
9-OH	-	6.49, br s		10
17-OH	-	13.35, s	16, 17, 18	20
NH	-	8.95, s	1, 2, 19, 20	20

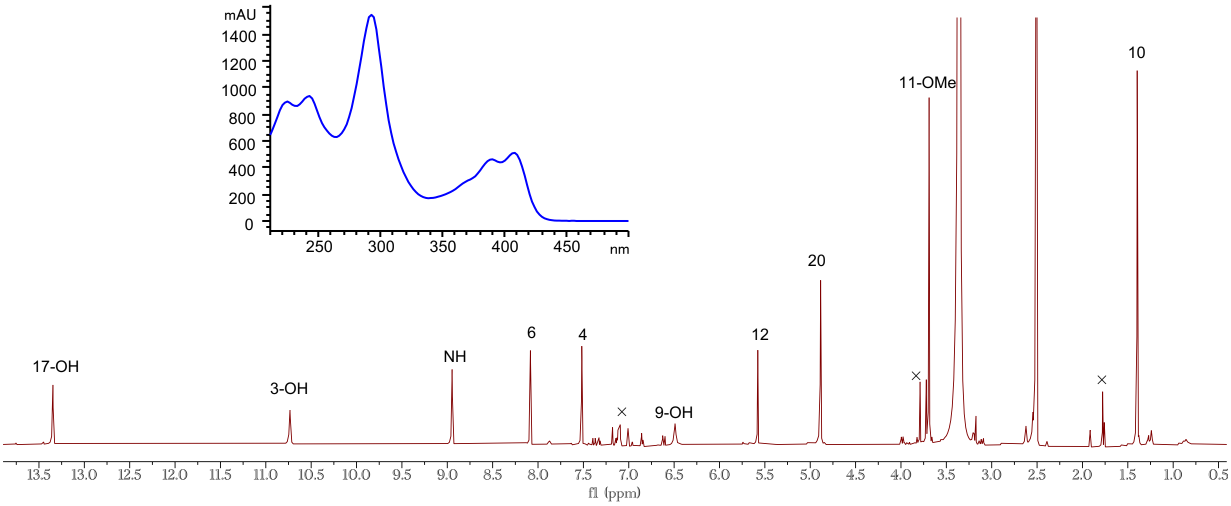

Figure S20. ^{1}H NMR (DMSO-d6) and UV-vis (inset) spectra of goondicone C (**3**).

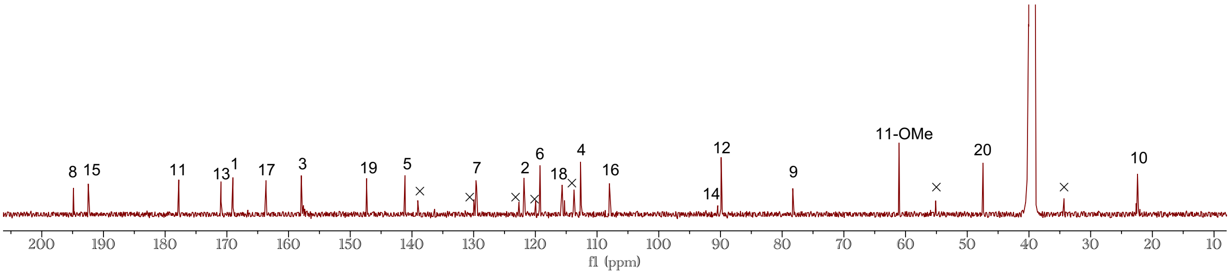

Figure S21. 13C NMR (DMSO-d6) spectrum of goondicone C (**3**).

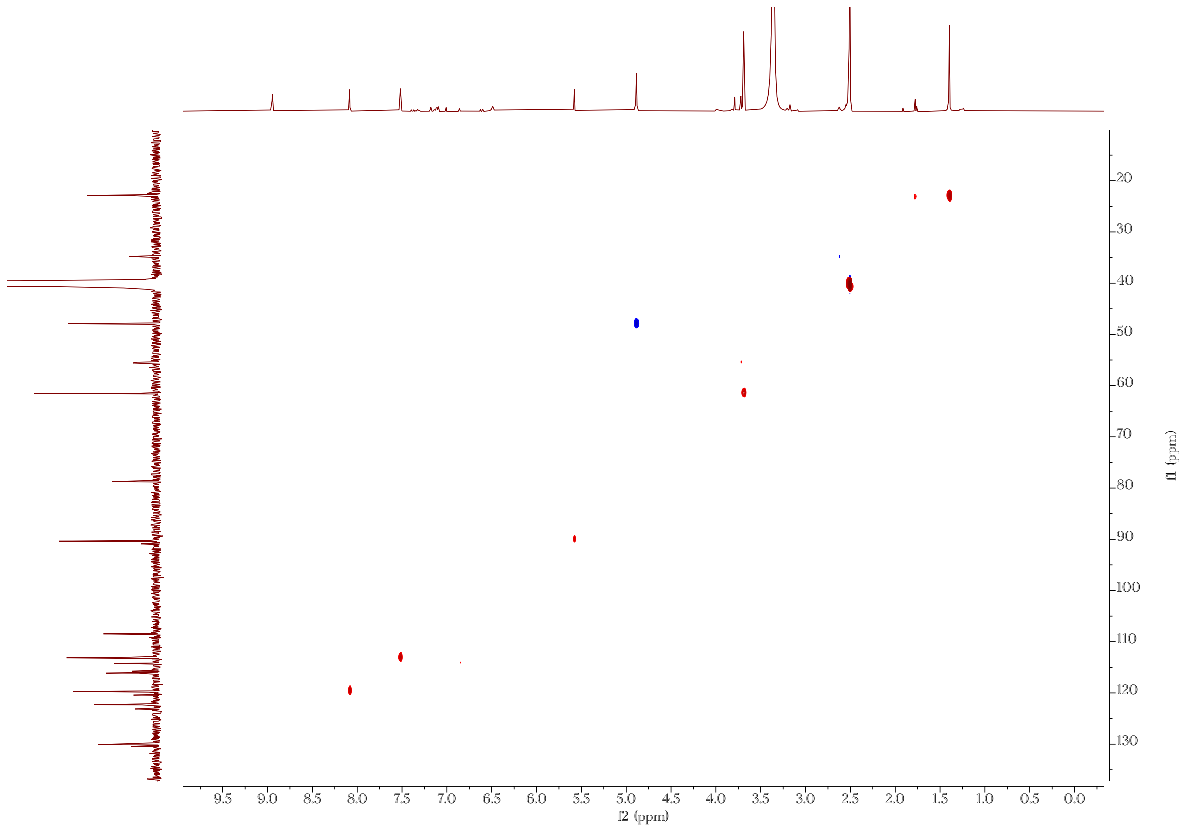

Figure S22. HSQC NMR (DMSO-d_6) spectrum of goondicone C (**3**).

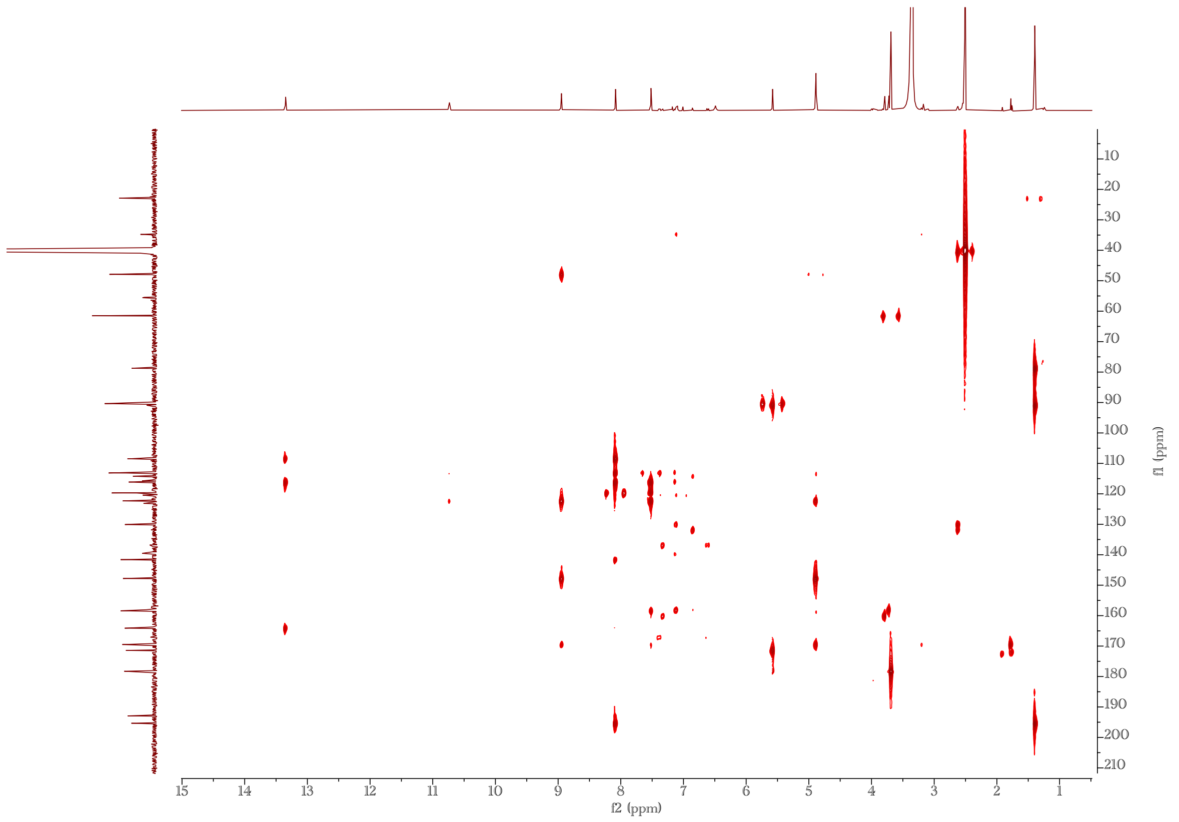

Figure S23. HMBC NMR (DMSO-d6) spectrum of goondicone C (**3**).

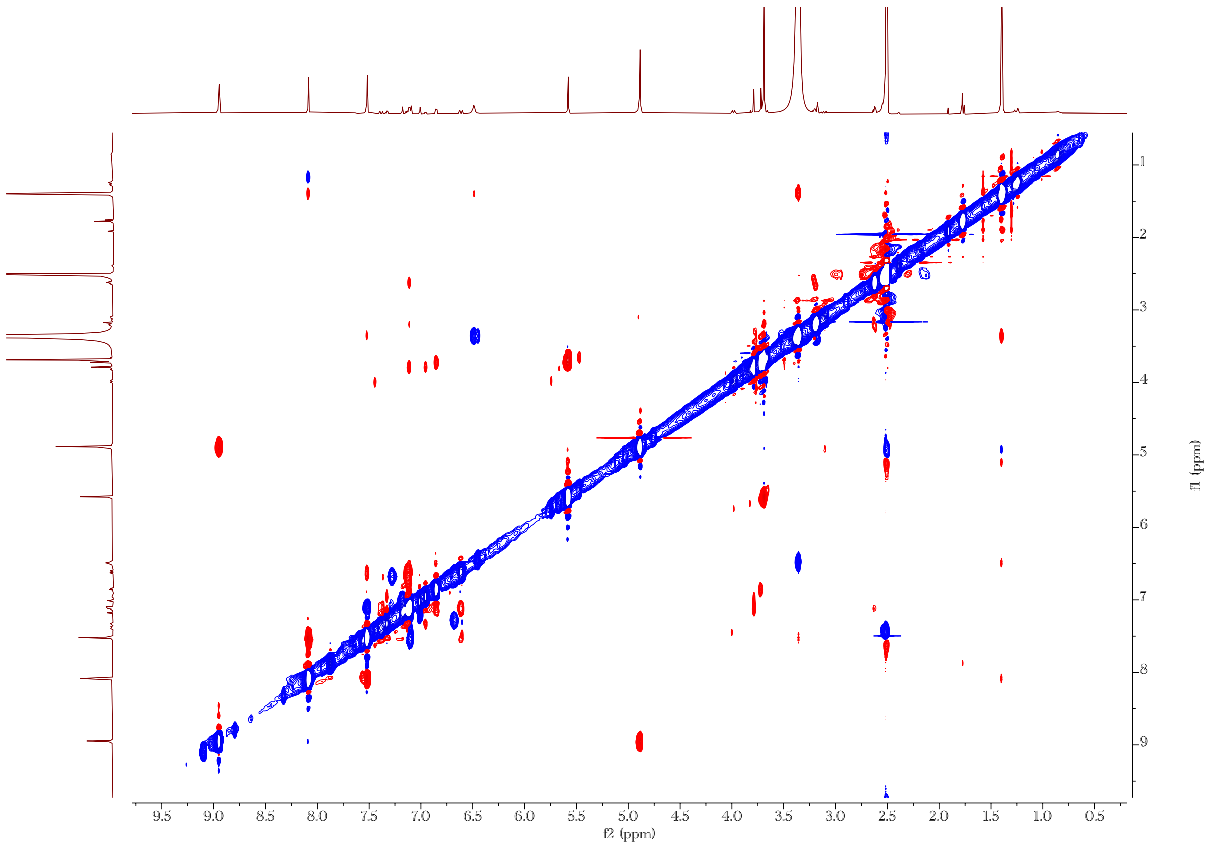

Figure S24. ROESY NMR (DMSO-d_6) spectrum of goondicone C (**3**).

Mass Spectrum Molecular Formula Report

Analysis Info

Analysis Name	D:\Data\Jianying\S4S-00185A06-L-F1314-P5.d	Acquisition Date	2/15/2022 1:24:33 PM	
Method	tune-medhigh_AP.m	Operator	a.salim	
Sample Name	S4S-00185A06-L-F1314-P5	Instrument / Ser#	micrOTOF	213750.00232
Comment				

Acquisition Parameter

Source Type	ESI	Ion Polarity	Positive	Set Nebulizer	0.5 Bar
Focus	Not active			Set Dry Heater	180 °C
Scan Begin	100 m/z	Set Capillary	4500 V	Set Dry Gas	5.0 l/min
Scan End	1500 m/z	Set End Plate Offset	-500 V	Set Divert Valve	Source

Generate Molecular Formula Parameter

Formula, min.		
Formula, max.		
Measured m/z	Tolerance	Charge
Check Valence	Minimum	Maximum
Nirogen Rule	Electron Configuration	
Filter H/C Ratio	Minimum	Maximum
Estimate Carbon		

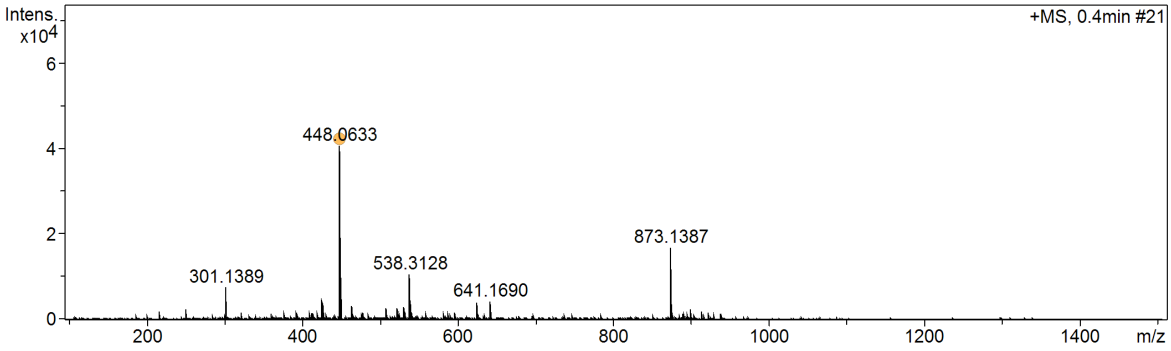

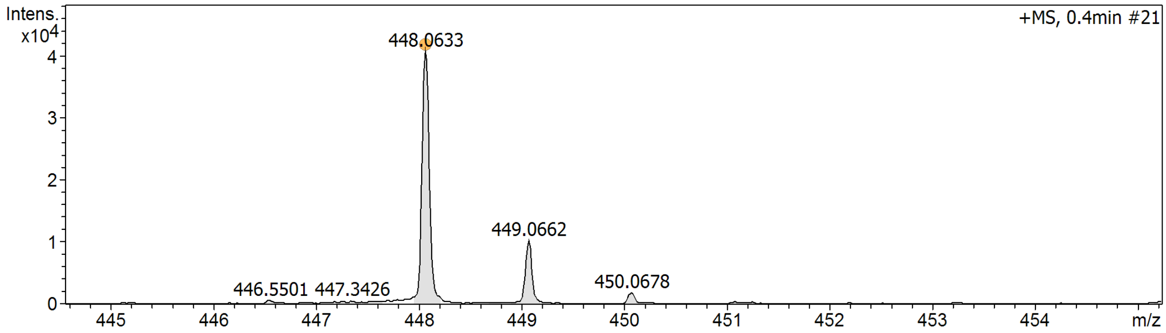

Meas. m/z	#	Ion Formula	m/z	err [ppm]	mSigma	# Sigma	Score	rdb	e⁻ Conf	N-Rule
448.0633	1	C21H15NNaO9	448.0639	1.3	8.1	1	100.00	14.5	even	ok

Figure S25. HRMS spectrum and measurement for goondicone C (**3**).

Spectroscopic characterisation goondicone D (4)

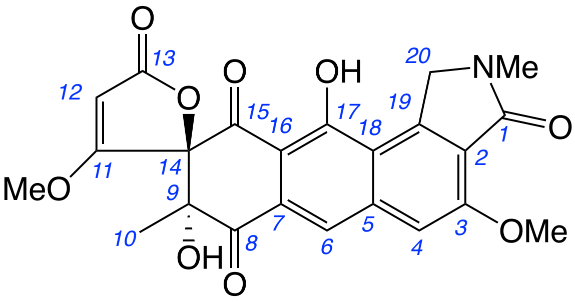

(**4**)

Table S5. 1D and 2D NMR (DMSO-d_6) data for goondicone D (**4**)

position	δ_C, type	δ_H, mult.	HMBC	ROESY
1	164.7, C	-	-	-
2	123.1, C	-	-	-
3	158.7, C	-	-	-
4	109.4, CH	7.82, s	2, 3, 6, 18	6, 3-OMe
5	140.8, C	-	-	-
6	119.4, CH	8.19, s	4, 5, 8, 16, 18	4
7	129.8, C	-	-	-
8	194.8, C	-	-	-
9	78.3, C	-	-	-
10	22.4, CH_3	1.41, s	8, 9, 14	9-OH
11	177.7, C	-	-	-
12	89.9, CH	5.59, s	11, 13, 14	11-OMe
13	170.9, C	-	-	-
14	90.5, C	-	-	-
15	192.7, C	-	-	-
16	108.6, C	-	-	-
17	163.2, C	-	-	-
18	115.9, C	-	-	-
19	145.2, C	-	-	-
20	53.1, CH_2	4.95, s	1, 2, 19	*N*-Me
N-Me	28.9, CH_3	3.12, s	1, 20	20
11-OMe	61.1, CH_3	3.69, s	11	12
3-OMe	56.2, CH_3	4.03, s	3	4
9-OH	-	6.52, s	8, 9, 10, 14	10
17-OH	-	13.31, s	16, 17, 18	

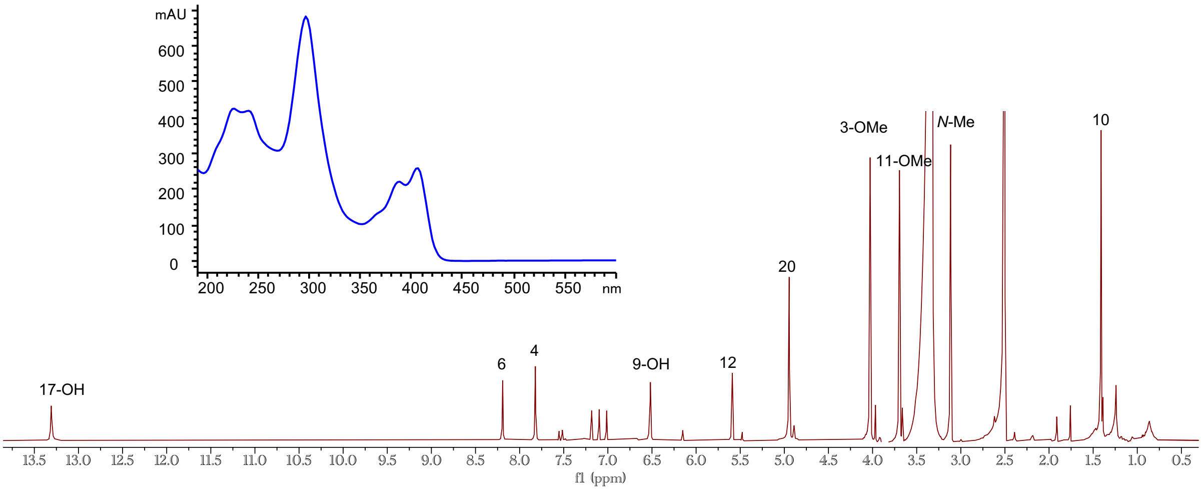

Figure S26. ^{1}H NMR (DMSO-d_6) and UV-vis (inset) spectra of goondicone D (**4**).

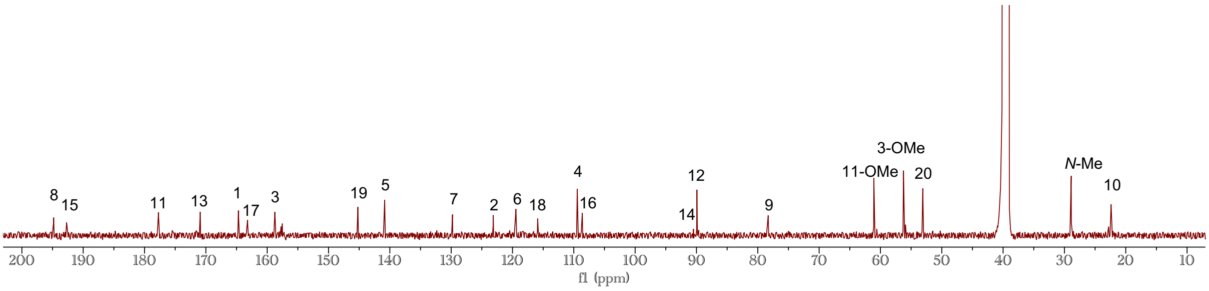

Figure S27. ^{13}C NMR (DMSO-d_6) spectrum of goondicone D (**4**).

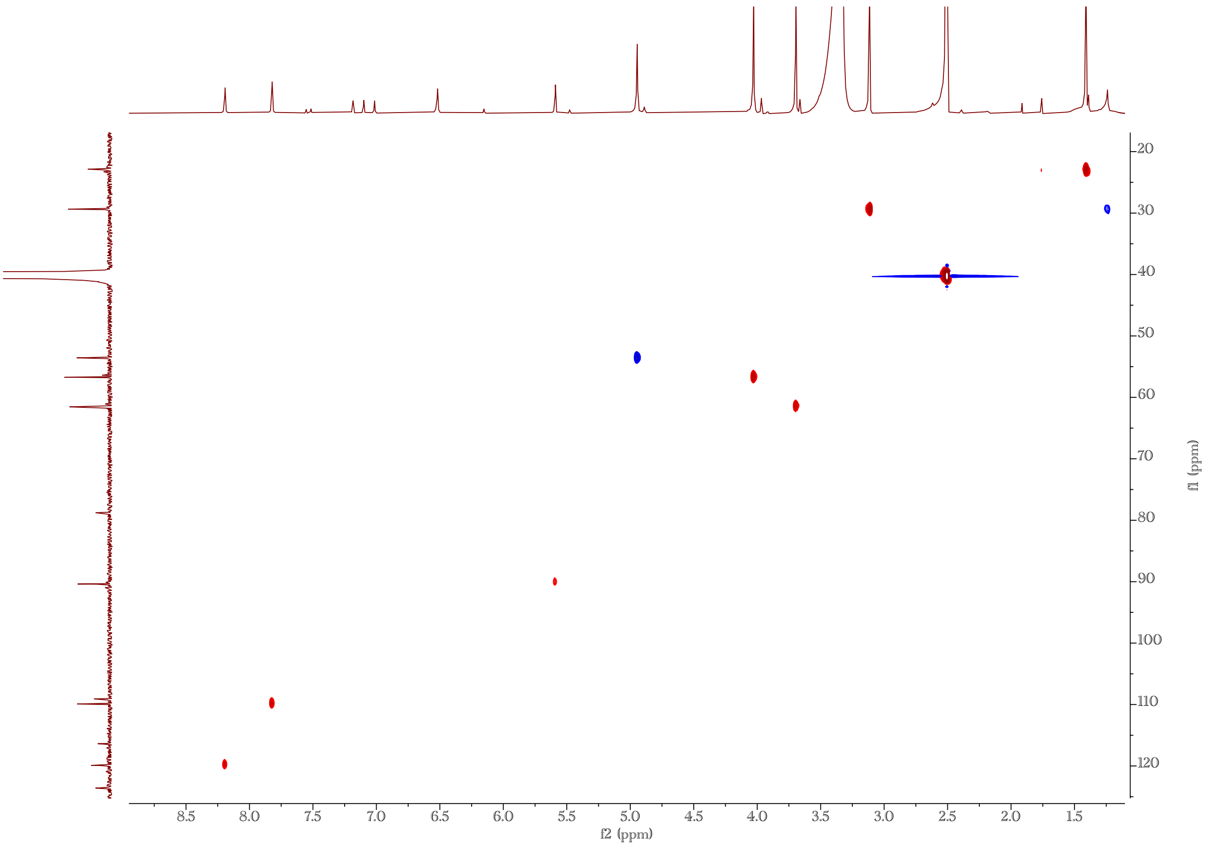

Figure S28. HSQC NMR (DMSO-d_6) spectrum of goondicone D (**4**).

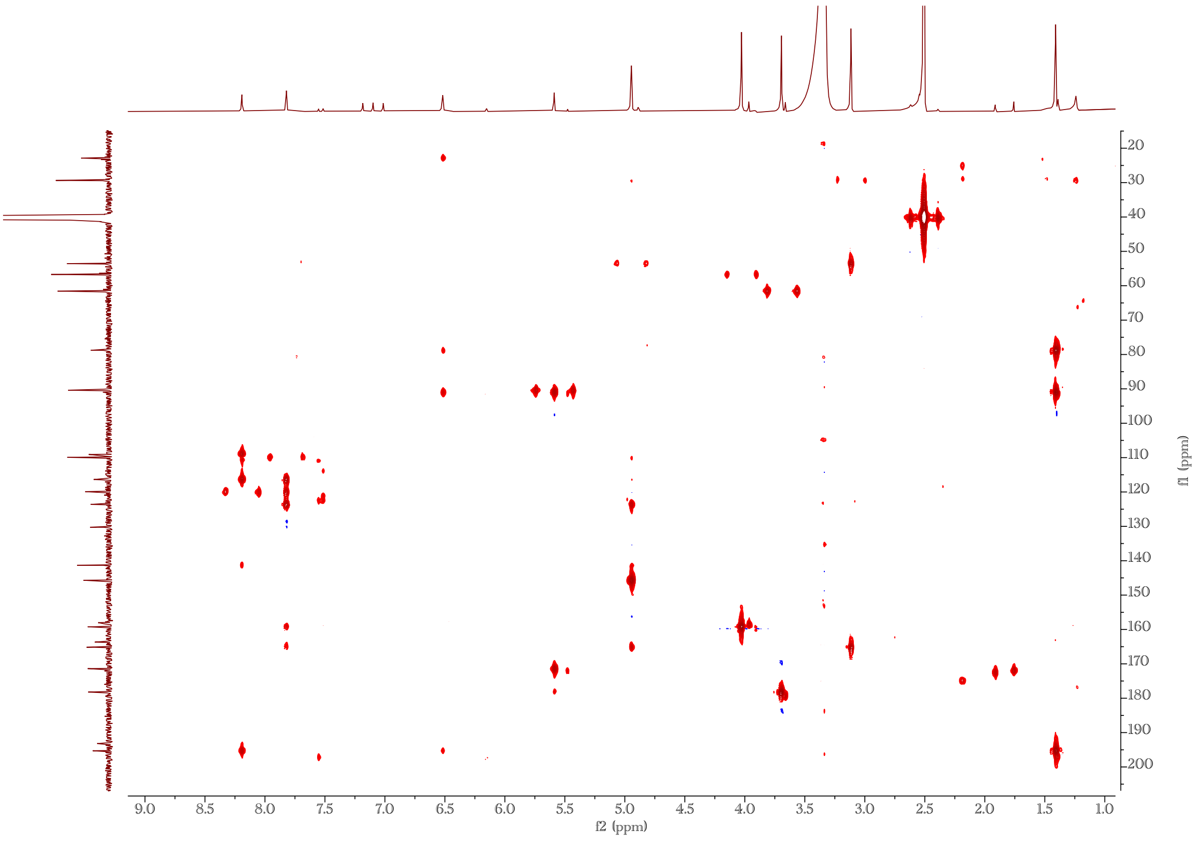

Figure S29. HMBC NMR (DMSO-d_6) spectrum of goondicone D (**4**).

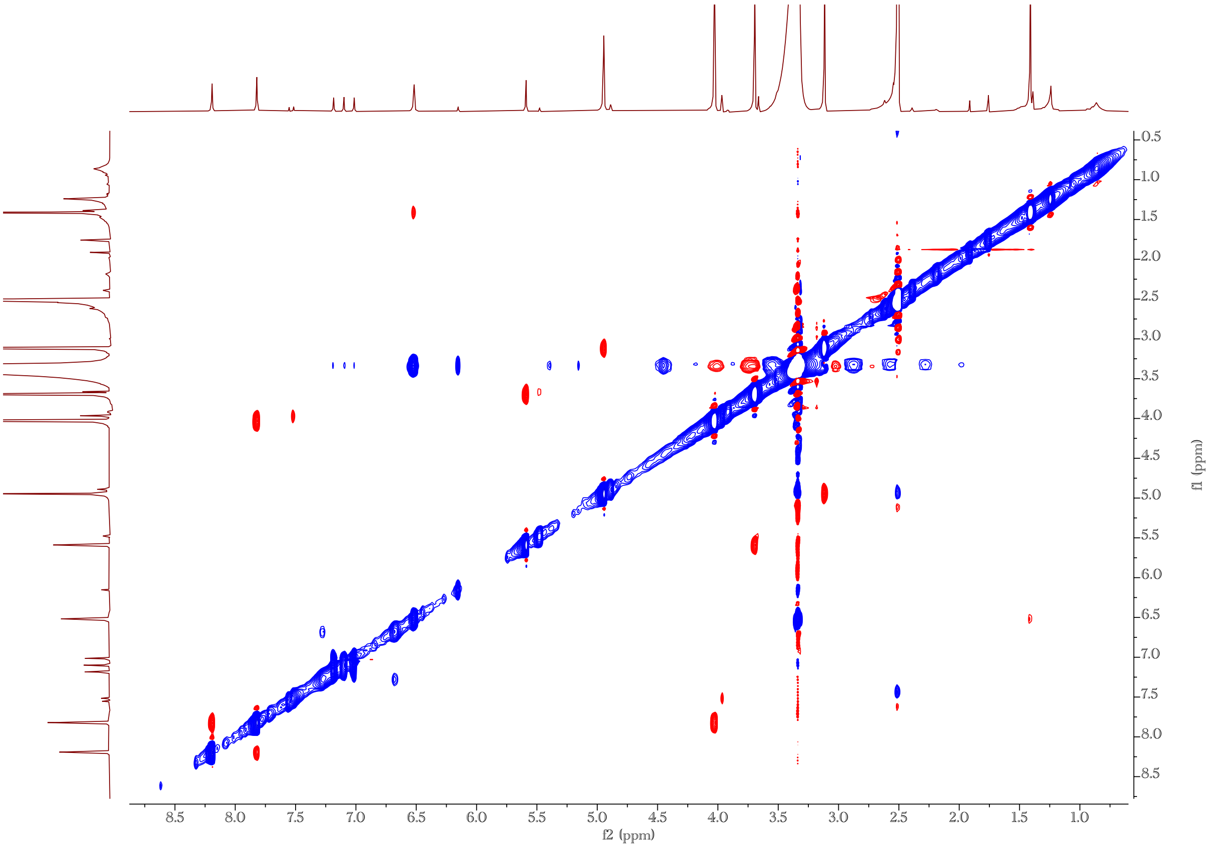

Figure S30. ROESY NMR (DMSO-d_6) spectrum of goondicone D (**4**).

Mass Spectrum Molecular Formula Report

Analysis Info

Analysis Name	D:\Data\Jianying\S4S-00185A06-L-F15-P4000001.d	Acquisition Date	2/24/2022 10:06:27 AM
Method	tune-medhigh_AP.m	Operator	a.salim
Sample Name	S4S-00185A06-L-F15-P4	Instrument / Ser#	micrOTOF 213750.00232
Comment			

Acquisition Parameter

Source Type	ESI	Ion Polarity	Positive	Set Nebulizer	0.5 Bar
Focus	Not active			Set Dry Heater	180 °C
Scan Begin	100 m/z	Set Capillary	4500 V	Set Dry Gas	5.0 l/min
Scan End	1500 m/z	Set End Plate Offset	-500 V	Set Divert Valve	Source

Generate Molecular Formula Parameter

Formula, min.		
Formula, max.		
Measured m/z	Tolerance	Charge
Check Valence	Minimum	Maximum
Nirogen Rule	Electron Configuration	
Filter H/C Ratio	Minimum	Maximum
Estimate Carbon		

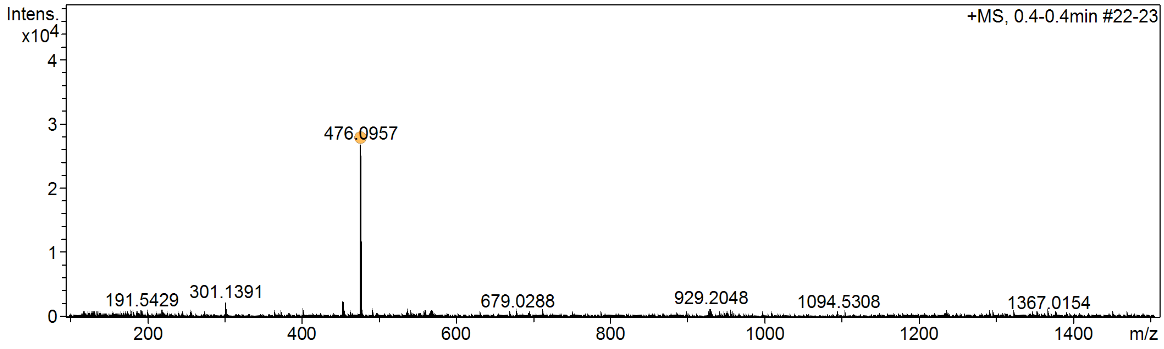

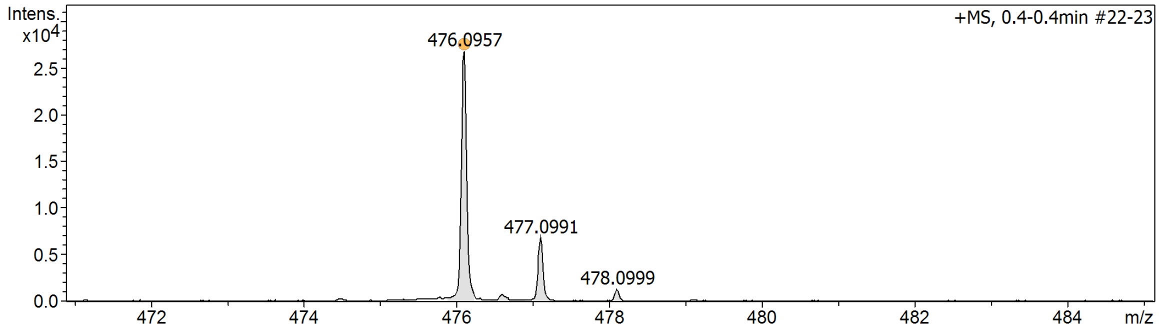

Meas. m/z	#	Ion Formula	m/z	err [ppm]	mSigma	# Sigma	Score	rdb	e⁻ Conf	N-Rule
476.0957	1	C23H19NNaO9	476.0952	1.1	1.9	1	100.00	14.5	even	ok

Figure S31. HRMS spectrum and measurement for goondicone D (**4**).

Spectroscopic characterisation goondicone E (5)

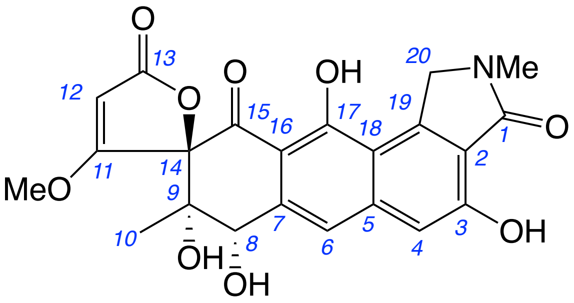

(**5**)

Table S6. 1D and 2D NMR (DMSO-d_6) data for goondicone E (**5**)

position	δ_C, type	δ_H, mult. (J in Hz)	HMBC	ROESY
1	166.6, C	-	-	-
2	119.5, C	-	-	-
3	156.8, C	-	-	-
4	109.9, CH	7.17, s	2, 3, 5, 6, 18	6
5	142.0, C	-	-	-
6	115.8, CH	7.48, s	4, 5, 8, 16, 18	4, 8, 8-OH
7	141.7, C	-	-	-
8	69.7, CH	5.03, d (9.3)	6, 7, 10, 16	6, 10, 11-OMe
9	77.0, C	-	-	-
10	18.7, CH_3	1.28, s	8, 9, 14	8, 9-OH
11	178.6, C	-	-	-
12	90.6, CH	5.67, s	11, 13, 14	11-OMe
13	171.5, C	-	-	-
14	89.7, C	-	-	-
15	194.9, C	-	-	-
16	108.0, C	-	-	-
17	163.4, C	-	-	-
18	112.4, C	-	-	-
19	144.9, C	-	-	-
20	53.7, CH_2	4.95, ABq (20.2)	1, 2, 19	*N*-Me
		4.90, ABq (20.2)	1, 2, 19	*N*-Me
N-Me	28.6, CH_3	3.12, s	1, 20	20
11-OMe	61.1, CH_3	3.82, s	11	8, 10, 12
3-OH	-	10.42, s	2, 3, 4	
8-OH	-	5.72, d (9.3)	7, 8, 9	6
9-OH	-	5.74, s	8, 9, 10, 14	10
17-OH	-	13.77, s	16, 17, 18	20

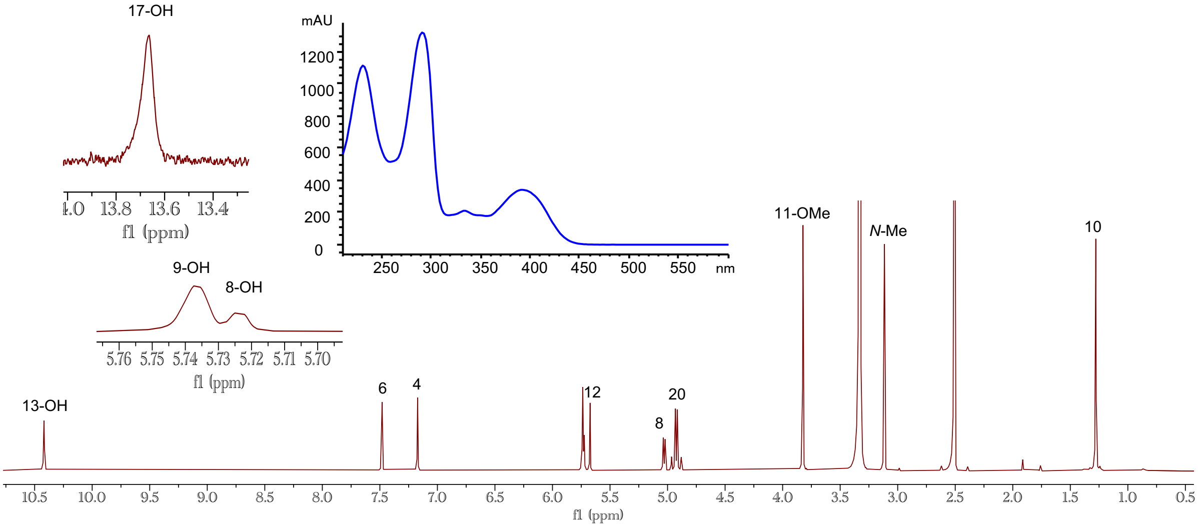

Figure S32. ^{1}H NMR (DMSO-d_6) and UV-vis (inset) spectra of goondicone E (**5**)

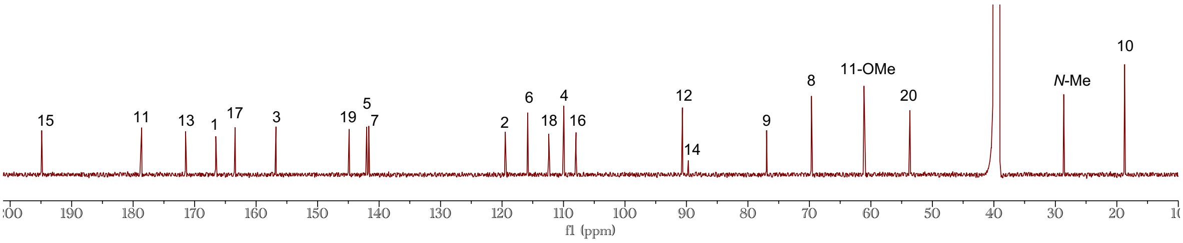

Figure S33. ^{13}C NMR (DMSO-d_6) spectrum of goondicone E (**5**)

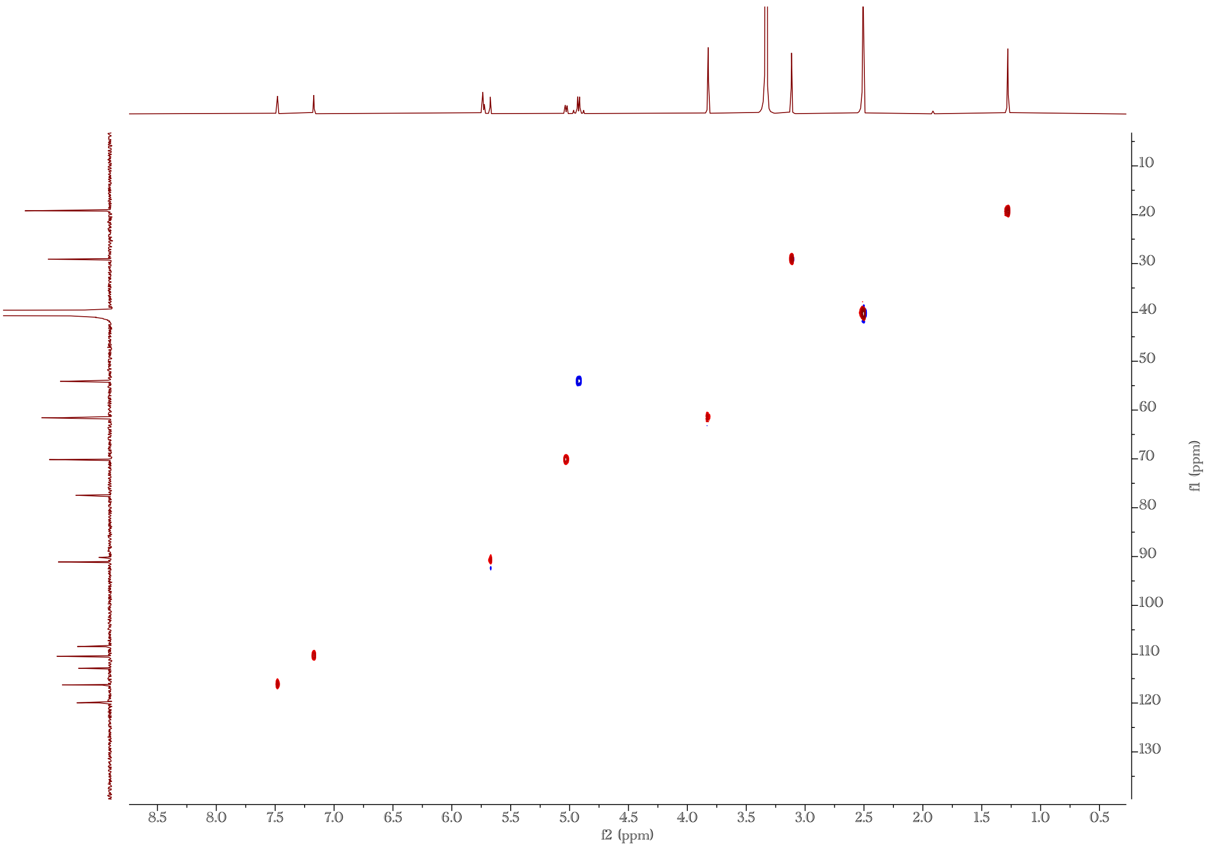

Figure S34. HSQC NMR (DMSO-d_6) spectrum of goondicone E (**5**)

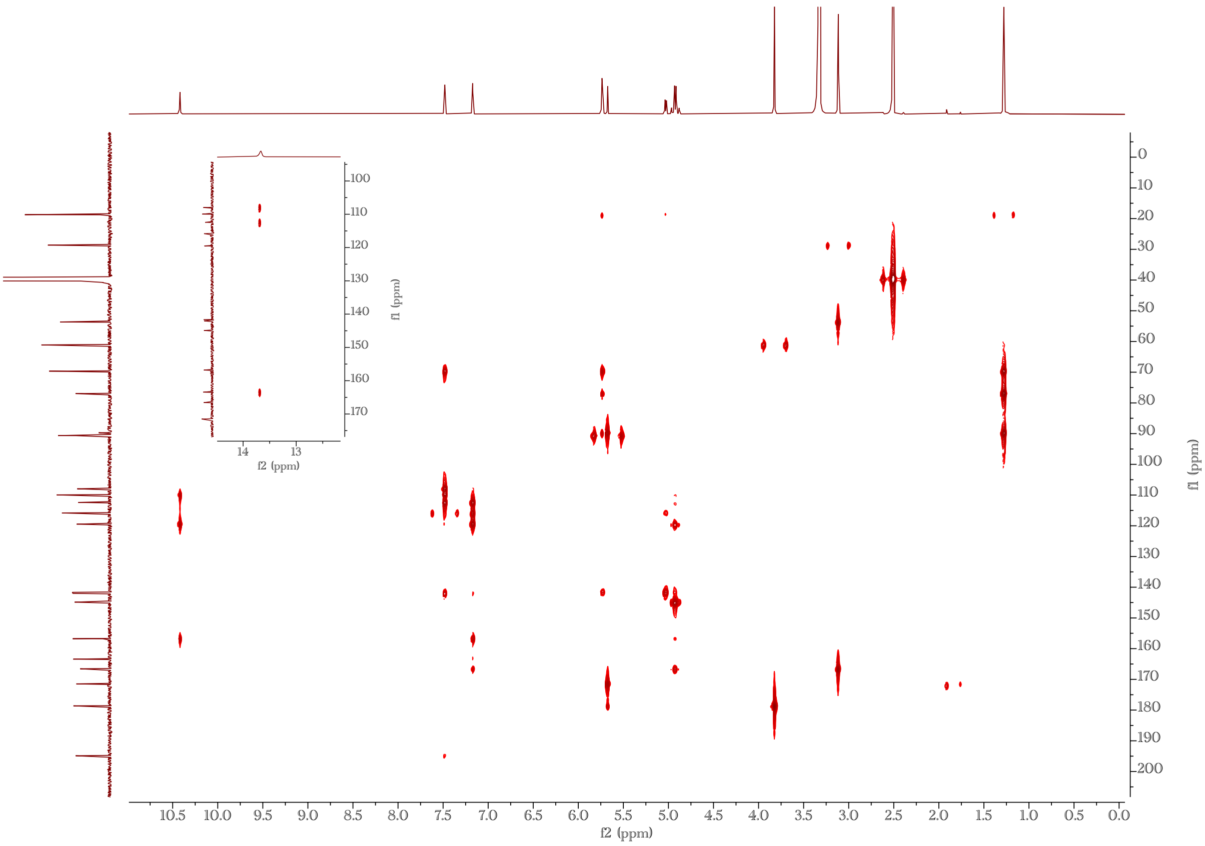

Figure S35. HMBC NMR (DMSO-d_6) spectrum of goondicone E (**5**)

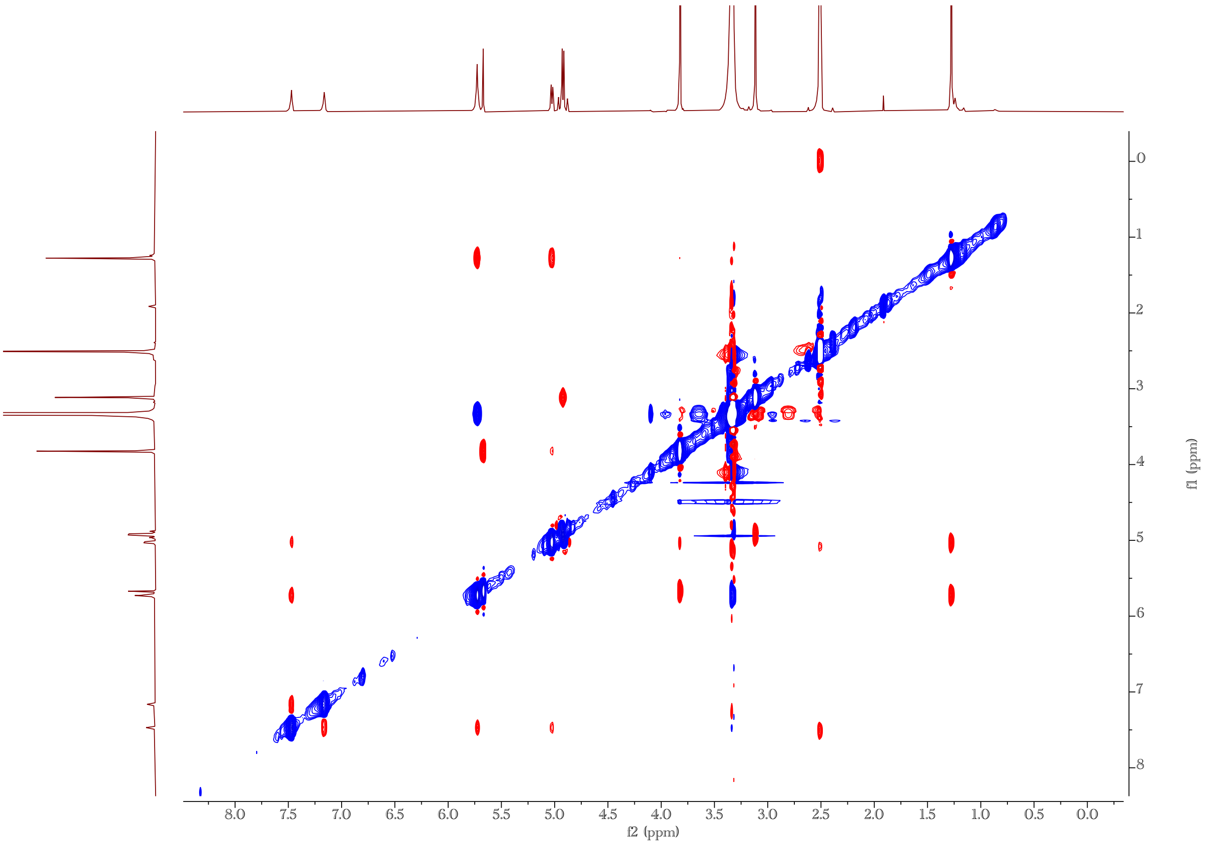

Figure S36. ROESY NMR (DMSO-d_6) spectrum of goondicone E (**5**)

Mass Spectrum Molecular Formula Report

Analysis Info

Analysis Name	D:\Data\Jianying\S4S-00185A06-P1.d	Acquisition Date	12/2/2021 3:44:28 PM	
Method	tune-medhigh_AP.m	Operator	a.salim	
Sample Name		Instrument / Ser#	micrOTOF	213750.00232
Comment				

Acquisition Parameter

Source Type	ESI	Ion Polarity	Positive	Set Nebulizer	0.5 Bar
Focus	Not active			Set Dry Heater	180 °C
Scan Begin	100 m/z	Set Capillary	4500 V	Set Dry Gas	5.0 l/min
Scan End	1500 m/z	Set End Plate Offset	-500 V	Set Divert Valve	Source

Generate Molecular Formula Parameter

Formula, min.
Formula, max.
Measured m/z — Tolerance — Charge
Check Valence — Minimum — Maximum
Nirogen Rule — Electron Configuration
Filter H/C Ratio — Minimum — Maximum
Estimate Carbon

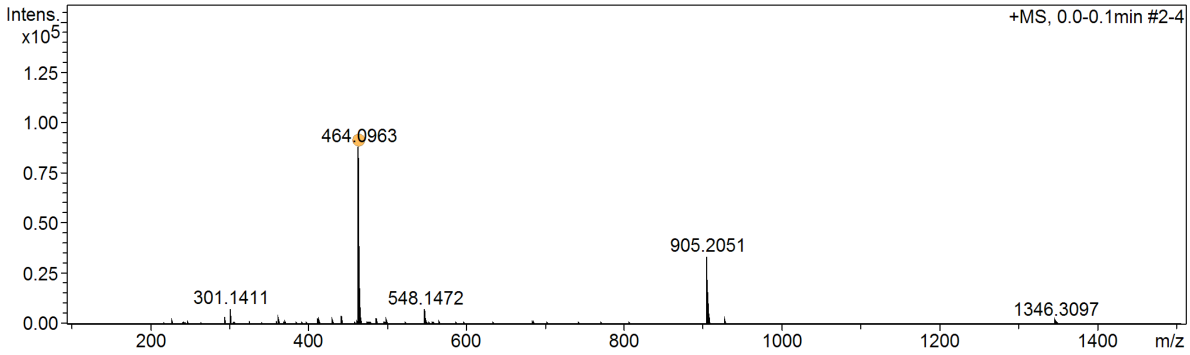

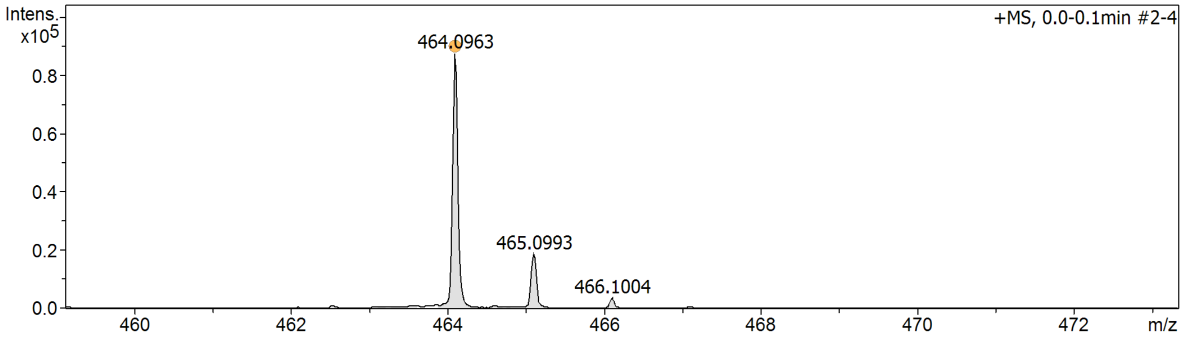

Meas. m/z	#	Ion Formula	m/z	err [ppm]	mSigma	# Sigma	Score	rdb	e⁻ Conf	N-Rule
464.0963	1	C22H19NNaO9	464.0952	2.4	17.1	1	80.15	13.5	even	ok
	2	C23H15N5NaO5	464.0965	0.4	28.5	2	100.00	18.5	even	ok
	3	C24H11N9NaO	464.0979	3.3	40.2	3	35.60	23.5	even	ok

Figure S37. HRMS spectrum and measurement for goondicone E (**5**)

Spectroscopic characterisation goondicone F (6)

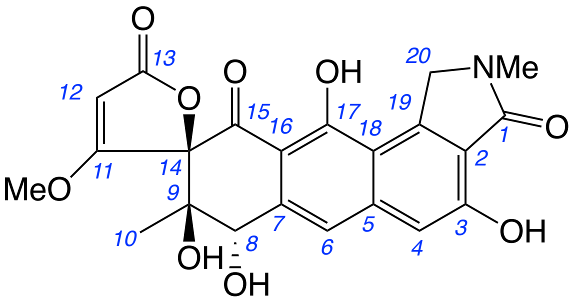

(**6**)

Table S7. 1D and 2D NMR (DMSO-d_6) data for goondicone F (**6**)

position	δ_C, type	δ_H, mult. (*J* in Hz)	HMBC	ROESY (need confirm)
1	166.5, C	-	-	-
2	119.6, C	-	-	-
3	157.1, C	-	-	-
4	110.1, CH	7.19, s	2, 3, 6, 18	6, 3-OH
5	142.0, C	-	-	-
6	115.5, CH	7.49, s	4, 8, 16, 18	4, 8, 8-OH
7	140.5, C	-	-	-
8	69.5, CH	5.30, d (6.2)	6, 7, 9, 10	6, 8-OH, 9-OH
9	76.6, C	-	-	-
10	17.0, CH_3	1.12, s	8, 9, 14	9-OH, 8-OH
11	179.7, C	-	-	-
12	90.4, CH	5.60, s	13, 14	11-OMe
13	171.5, C	-	-	-
14	90.6, C	-	-	-
15	194.5, C	-	-	-
16	107.6, C	-	-	-
17	164.2, C	-	-	-
18	112.4, C	-	-	-
19	145.1, C	-	-	-
20	53.6, CH_2	4.93, ABq (20.2)	1, 2, 19	*N*-Me
		4.89, ABq (20.2)	1, 2, 19	*N*-Me
N-Me	28.7, CH_3	3.11, s	1, 20	20
11-OMe	60.7, CH_3	3.80, s	11	12
3-OH	-	10.50, s	2, 3, 4	4
8-OH	-	6.13, d (6.2)	7, 8, 9	6, 8, 10
9-OH	-	5.88, s	8, 9, 10, 14	8, 10
17-OH	-	13.84, s	16, 17, 18	20

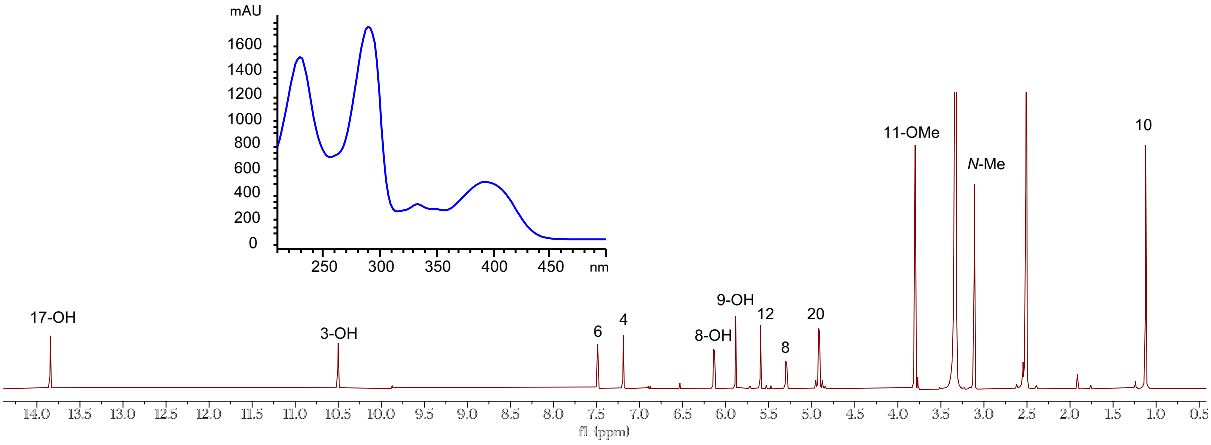

Figure S38. ^{1}H NMR (DMSO-d_6) and UV-vis (inset) spectra of goondicone F (**6**).

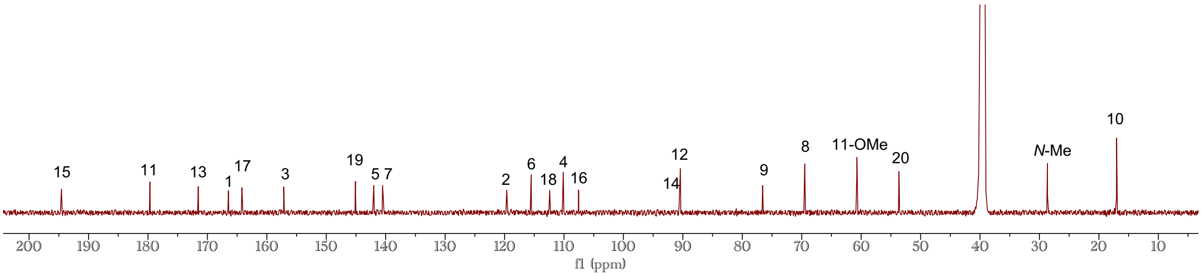

Figure S39. ^{13}C NMR (DMSO-d_6) spectrum of goondicone F (**6**).

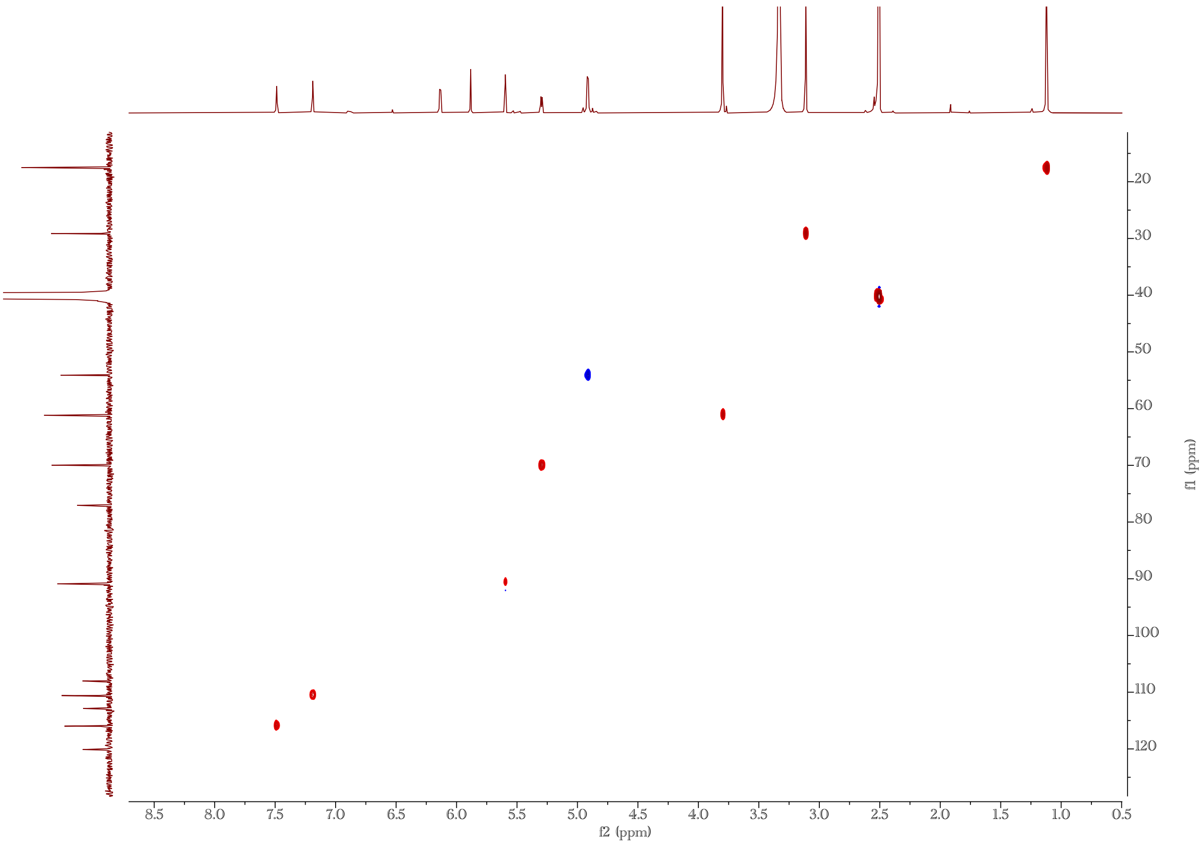

Figure S40. HSQC NMR (DMSO-d_6) spectrum of goondicone F (**6**).

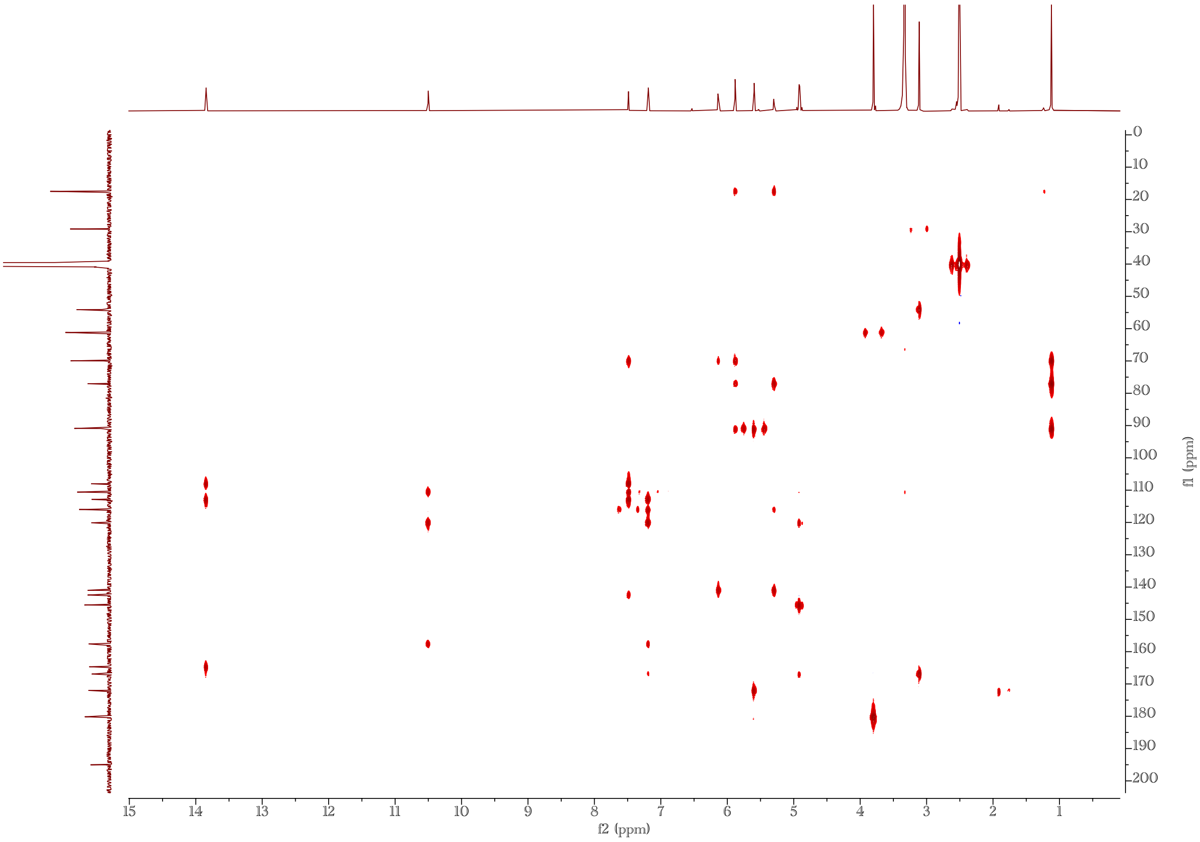

Figure S41. HMBC NMR (DMSO-d_6) spectrum of goondicone F (**6**).

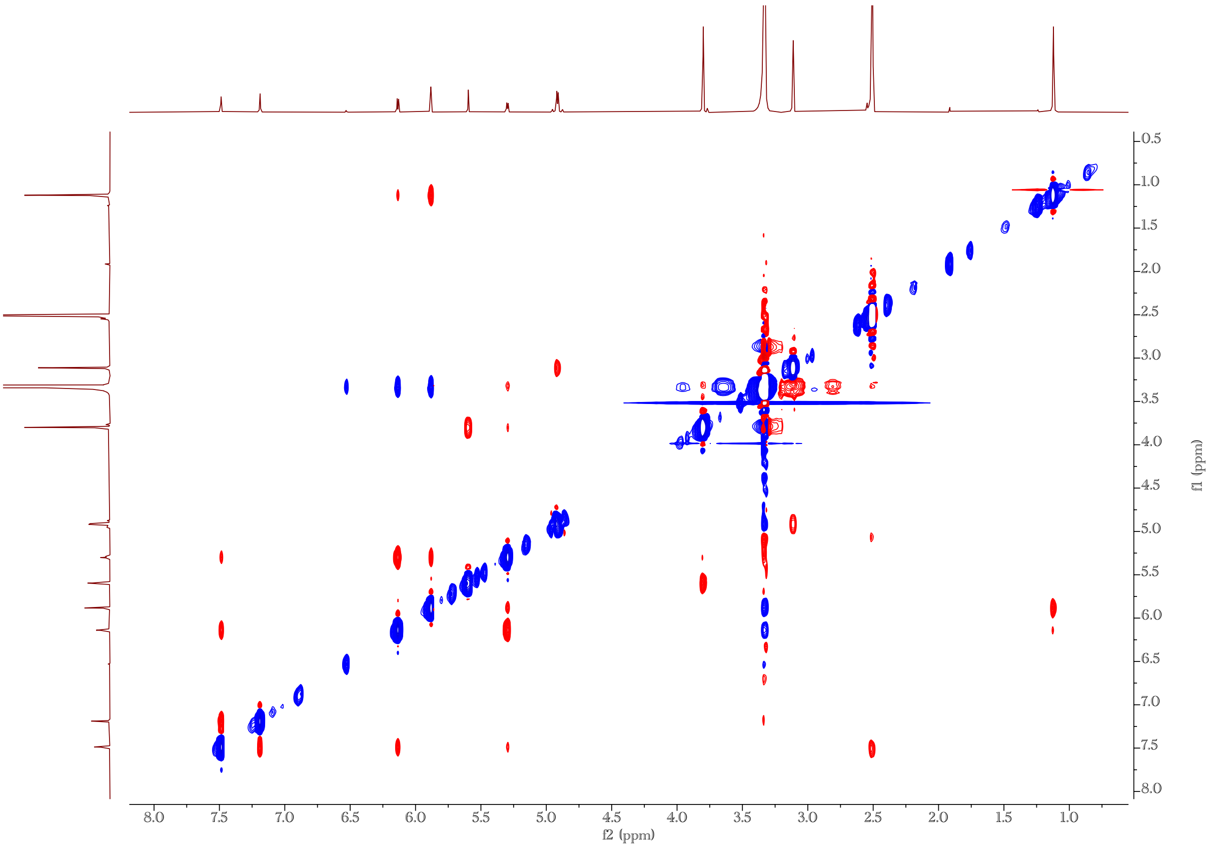

Figure S42. ROESY NMR (DMSO-d_6) spectrum of goondicone F (**6**).

Mass Spectrum Molecular Formula Report

Analysis Info

Analysis Name	D:\Data\Jianying\185A06-L-F1314-P6-3.d	Acquisition Date	2/18/2022 5:55:17 PM
Method	tune-medhigh_AP.m	Operator	a.salim
Sample Name	185A06-L-F1314-P6-3	Instrument / Ser#	micrOTOF 213750.00232
Comment			

Acquisition Parameter

Source Type	ESI	Ion Polarity	Positive	Set Nebulizer	0.5 Bar
Focus	Not active			Set Dry Heater	180 °C
Scan Begin	100 m/z	Set Capillary	4500 V	Set Dry Gas	5.0 l/min
Scan End	1500 m/z	Set End Plate Offset	-500 V	Set Divert Valve	Source

Generate Molecular Formula Parameter

Formula, min.		
Formula, max.		
Measured m/z	Tolerance	Charge
Check Valence	Minimum	Maximum
Nirogen Rule	Electron Configuration	
Filter H/C Ratio	Minimum	Maximum
Estimate Carbon		

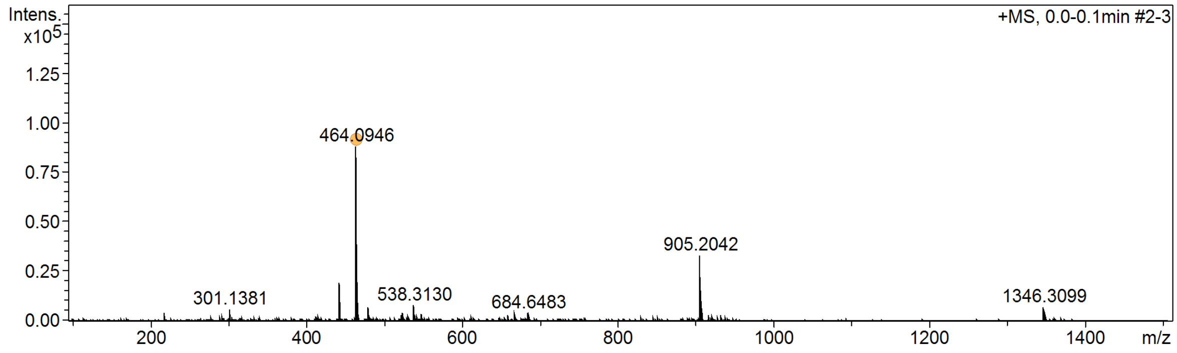

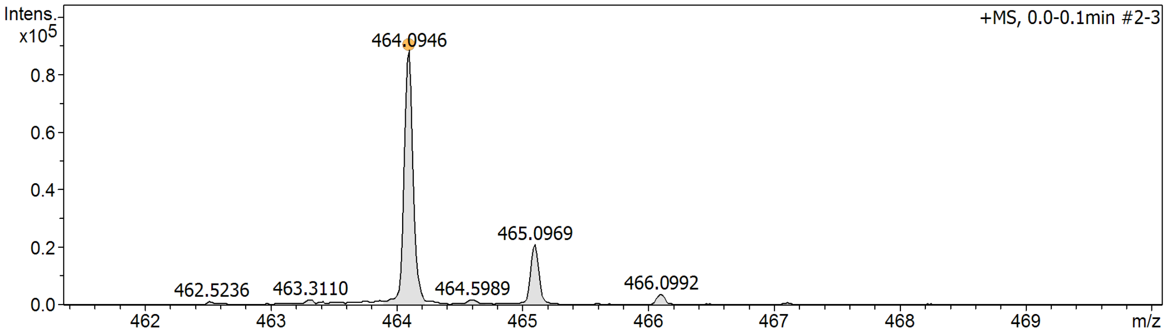

Meas. m/z	#	Ion Formula	m/z	err [ppm]	mSigma	# Sigma	Score	rdb	e⁻ Conf	N-Rule
464.0946	1	C22H19NNaO9	464.0952	-1.2	5.6	1	100.00	13.5	even	ok

Figure S43. HRMS spectrum and measurement for goondicone F (**6**).

Spectroscopic characterisation goondicone G (7)

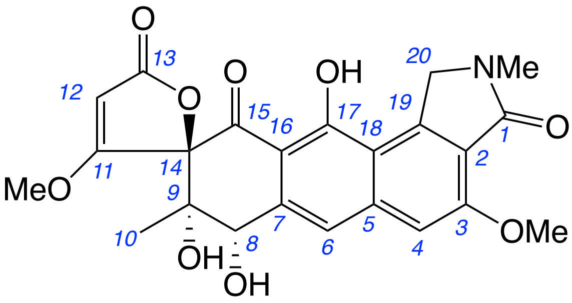

(**7**)

Table S8. 1D and 2D NMR (DMSO-d_6) data for goondicone G (**7**)

position	δ_C, type	δ_H, mult. (J in Hz)	HMBC	ROESY
1	165.2, C	-	-	-
2	120.7, C	-	-	-
3	158.3, C	-	-	-
4	106.7, CH	7.43, s	2, 3, 6, 18	6, 3-OMe
5	142.1, C	-	-	-
6	116.4, CH	7.65, s	4, 5, 7, 8, 16, 18	8, 4
7	142.1, C	-	-	-
8	69.7, CH	5.06, br s	7	6, 10
9	77.0, C	-	-	-
10	18.7, CH_3	1.29, s	8, 9, 14	8, 9-OH
11	178.6, C	-	-	-
12	90.7, CH	5.68, s	11, 13, 14	10, 11-OMe
13	171.5, C	-	-	-
14	89.8, C	-	-	-
15	195.1, C	-	-	-
16	108.4, C	-	-	-
17	163.1, C	-	-	-
18	112.7, C	-	-	-
19	145.2, C	-	-	-
20	53.1, CH_2	4.92, ABq (20.2)	1, 2, 19	*N*-Me
		4.86, ABq (20.2)	1, 2, 19	*N*-Me
N-Me	28.8, CH_3	3.09, s	1, 20	20
11-OMe	61.1, CH_3	3.83, s	11	12
3-OMe	56.0, CH_3	3.99, s	3	4
8-OH	-	5.77, br s		8
9-OH	-	5.77, br s		10
17-OH	-	13.70, s	16, 17, 18	20

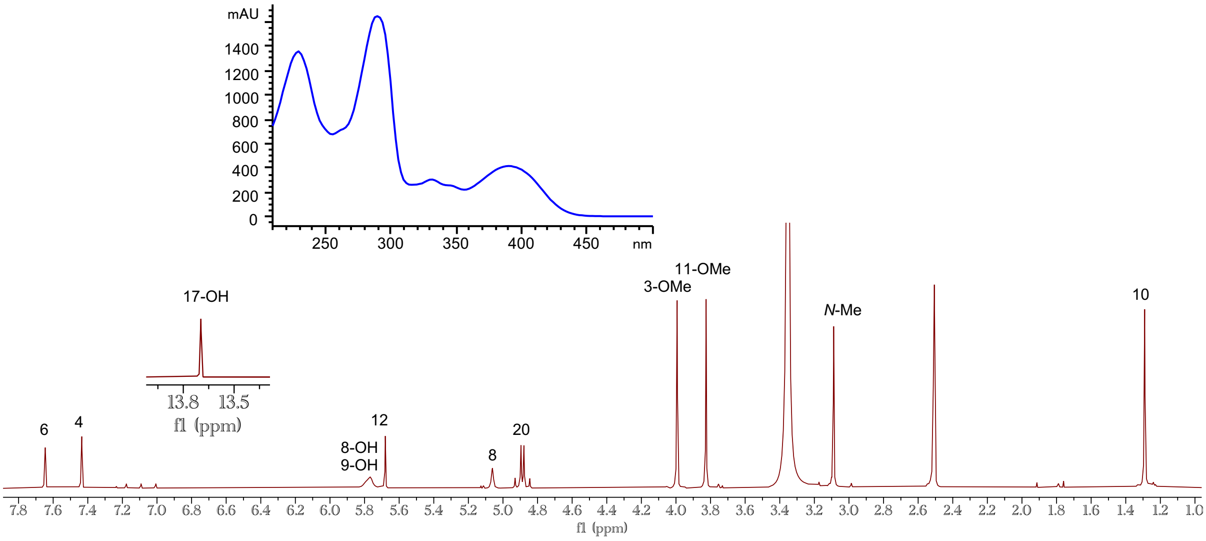

Figure S44. ^{1}H NMR (DMSO-d_6) and UV-vis (inset) spectra of goondicone G (**7**)

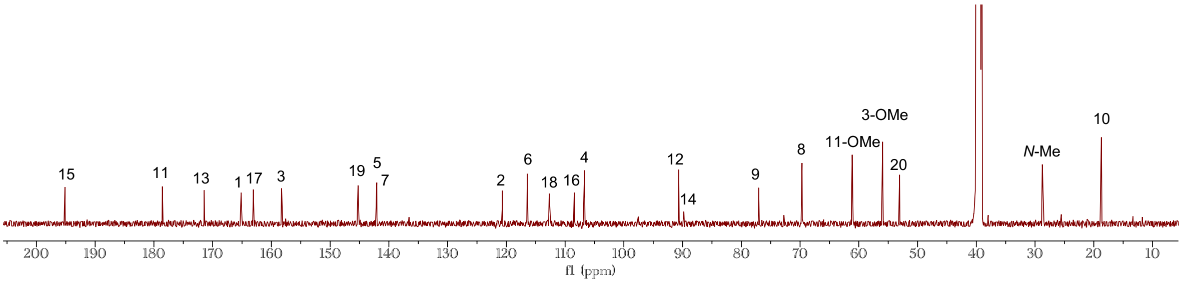

Figure S45. ^{13}C NMR (DMSO-d_6) spectrum of goondicone G (**7**)

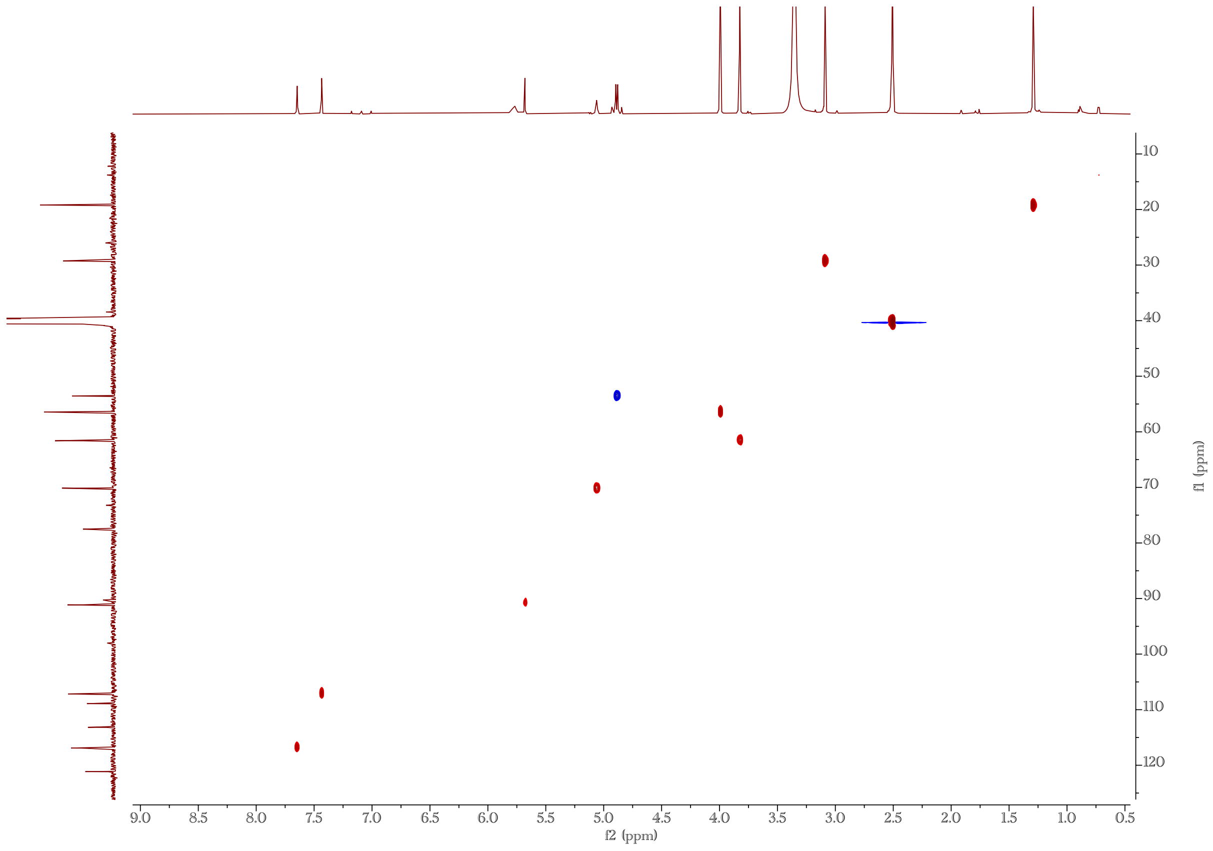

Figure S46. HSQC NMR (DMSO-d_6) spectrum of goondicone G (**7**).

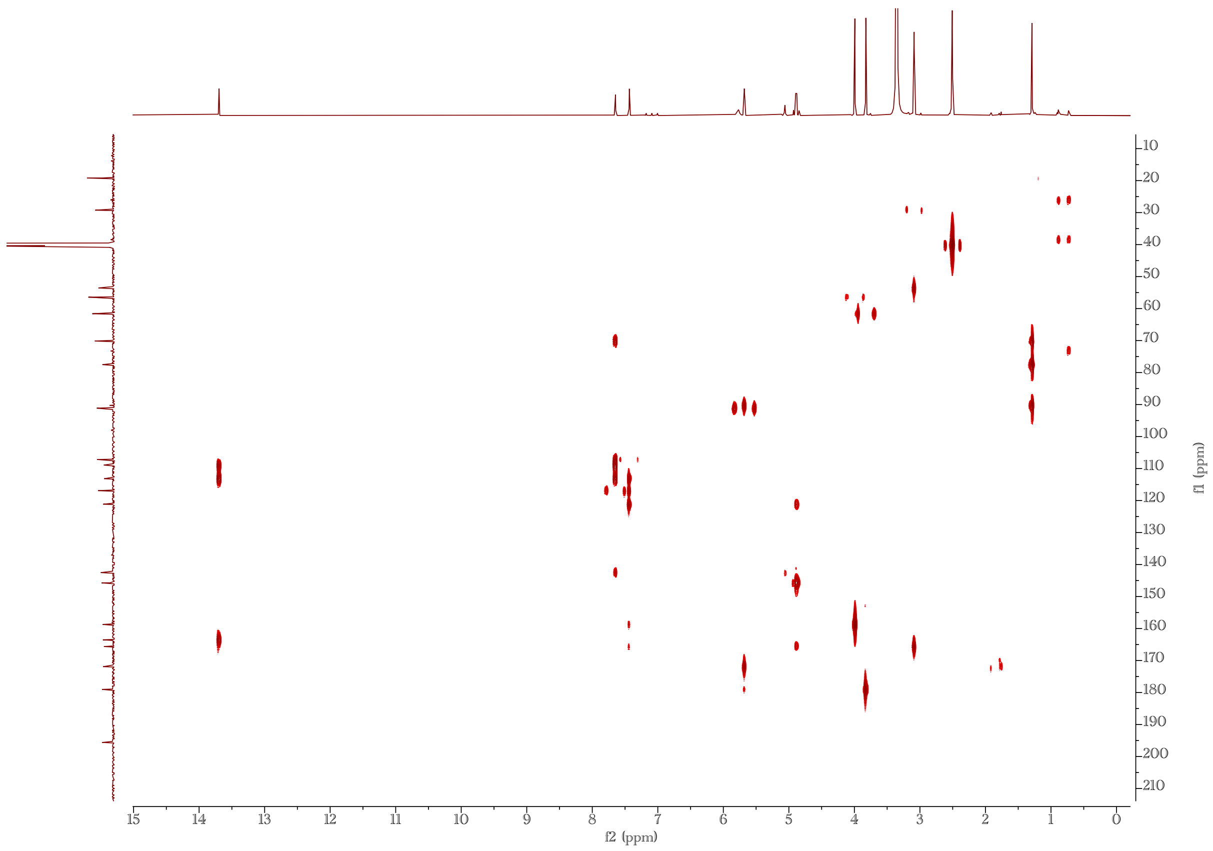

Figure S47. HMBC NMR (DMSO-d_6) spectrum of goondicone G (**7**).

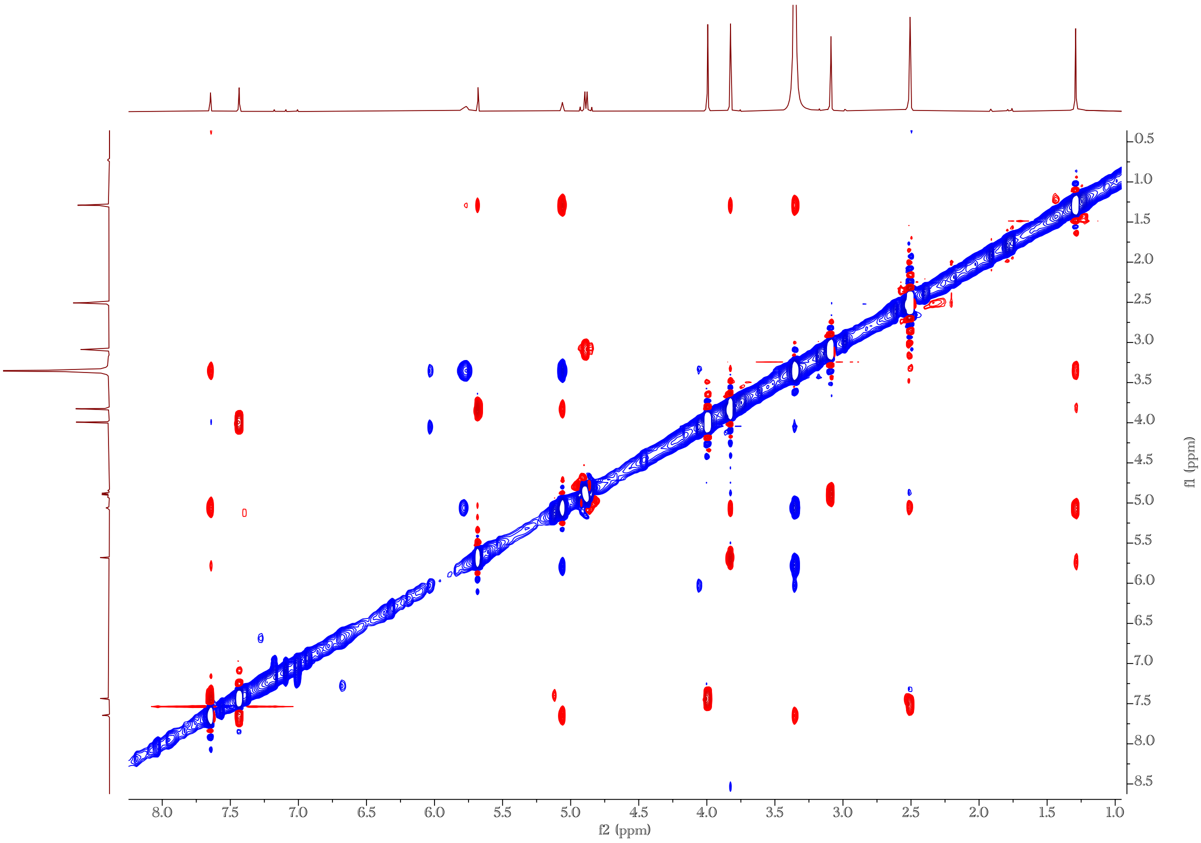

Figure S48. ROESY NMR (DMSO-d_6) spectrum of goondicone G (**7**).

Mass Spectrum Molecular Formula Report

Analysis Info

Analysis Name	D:\Data\Jianying\S4S-00185A06-L-F1314-P3.d	Acquisition Date	2/15/2022 1:18:07 PM	
Method	tune-medhigh_AP.m	Operator	a.salim	
Sample Name	S4S-00185A06-L-F1314-P3	Instrument / Ser#	micrOTOF	213750.00232
Comment				

Acquisition Parameter

Source Type	ESI	Ion Polarity	Positive	Set Nebulizer	0.5 Bar
Focus	Not active			Set Dry Heater	180 °C
Scan Begin	100 m/z	Set Capillary	4500 V	Set Dry Gas	5.0 l/min
Scan End	1500 m/z	Set End Plate Offset	-500 V	Set Divert Valve	Source

Generate Molecular Formula Parameter

Formula, min.		
Formula, max.		
Measured m/z	Tolerance	Charge
Check Valence	Minimum	Maximum
Nirogen Rule	Electron Configuration	
Filter H/C Ratio	Minimum	Maximum
Estimate Carbon		

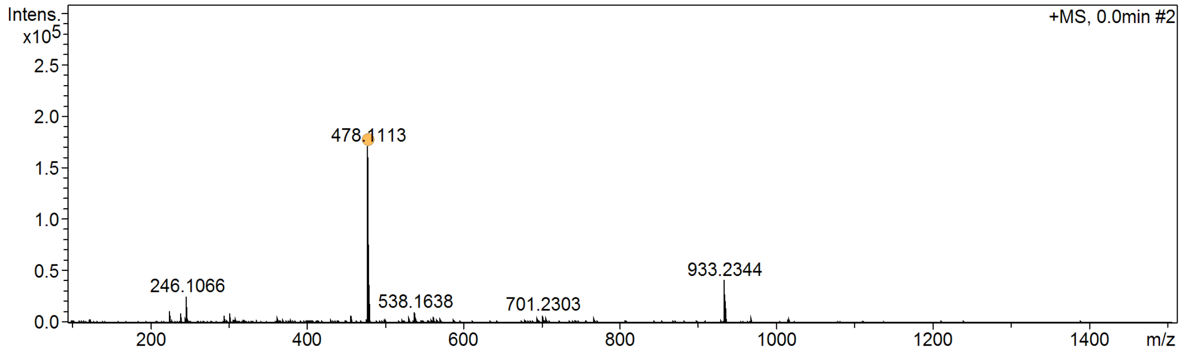

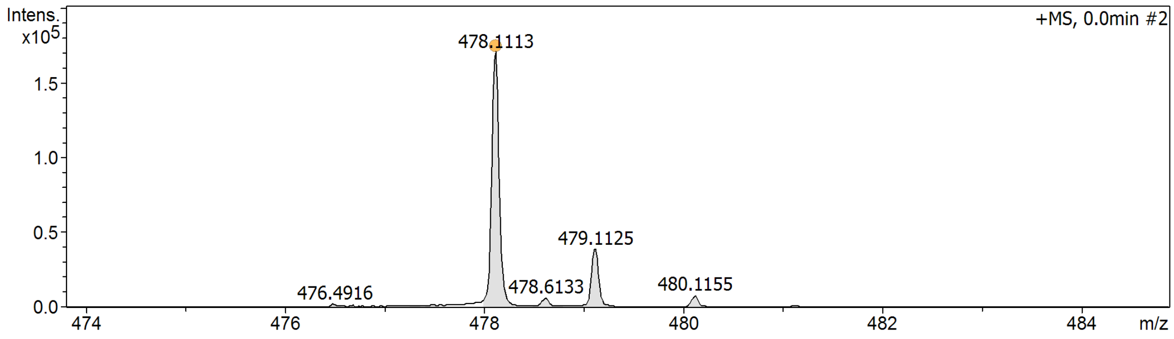

Meas. m/z	#	Ion Formula	m/z	err [ppm]	mSigma	# Sigma	Score	rdb	e¯ Conf	N-Rule
478.1113	1	C23H21NNaO9	478.1109	-1.0	15.5	1	100.00	13.5	even	ok

Figure S49. HRMS spectrum and measurement for goondicone G (**7**).

Spectroscopic characterisation goondicone H (8)

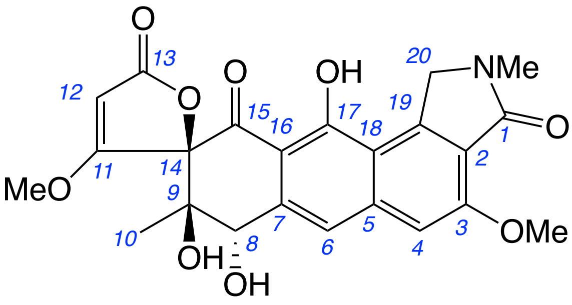

(**8**)

Table S9. 1D and 2D NMR (DMSO-d_6) data for goondicone H (**8**)

position	δ_C, type	δ_H, mult. (*J* in Hz)	HMBC	ROESY
1	165.1, C	-	-	-
2	120.8, C	-	-	-
3	158.6, C	-	-	-
4	106.9, CH	7.46, s	2, 3, 6, 18	6, 3-OMe
5	142.1, C	-	-	-
6	116.1, CH	7.66, s	5, 8, 16, 18	4, 8, 8-OH
7	141.0, C	-	-	-
8	69.5, CH	5.33, d (6.1)	6, 7, 9, 10	6, 8-OH
9	76.7, C	-	-	-
10	17.0, CH_3	1.13, s	8, 9, 14	8-OH, 9-OH
11	179.6, C	-	-	-
12	90.4, CH	5.61, s	13, 14	11-OMe
13	171.5, C	-	-	-
14	90.7, C	-	-	-
15	194.8, C	-	-	-
16	108.1, C	-	-	-
17	163.8, C	-	-	-
18	112.7, C	-	-	-
19	145.4, C	-	-	-
20	53.1, CH_2	4.91, ABq (20.2)	1, 2, 19	*N*-Me
		4.86, ABq (20.2)	1, 2, 19	*N*-Me
N-Me	28.8, CH_3	3.09, s	1, 20	20
11-OMe	60.7, CH_3	3.80, s	11	12
3-OMe	56.0, CH_3	3.99, s	3	4
8-OH	-	6.20, d (6.3)	7, 8	6, 8, 10
9-OH	-	5.90, s	8, 9, 10, 14	8(w), 10
17-OH	-	13.78, s	16, 17, 18	20

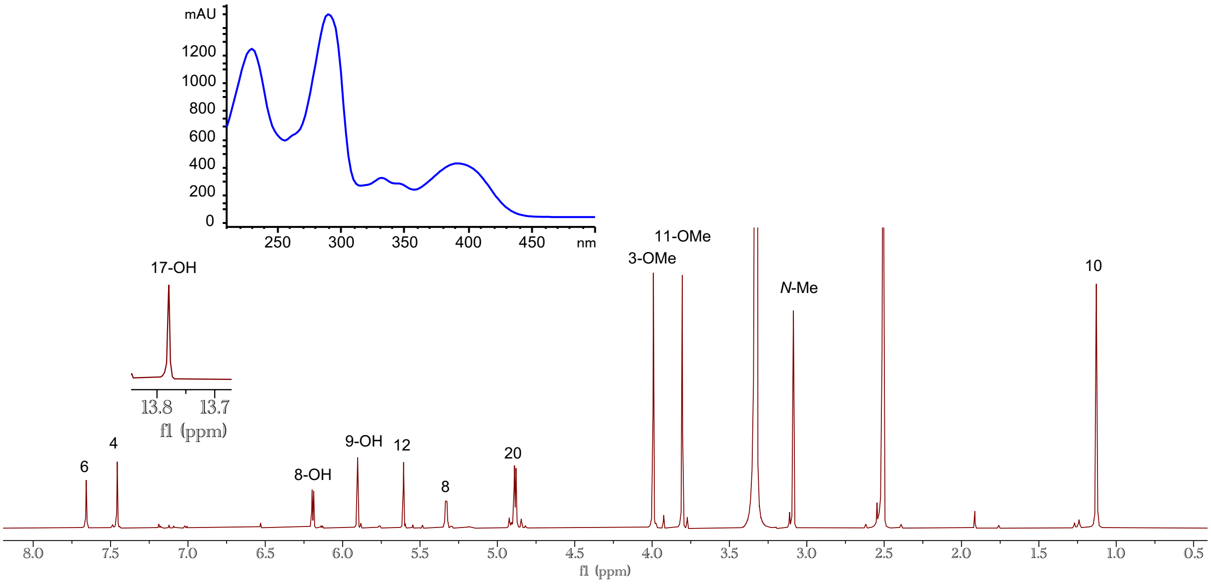

Figure S50. ^{1}H NMR (DMSO-d_6) and UV-vis (inset) spectra of goondicone H (**8**).

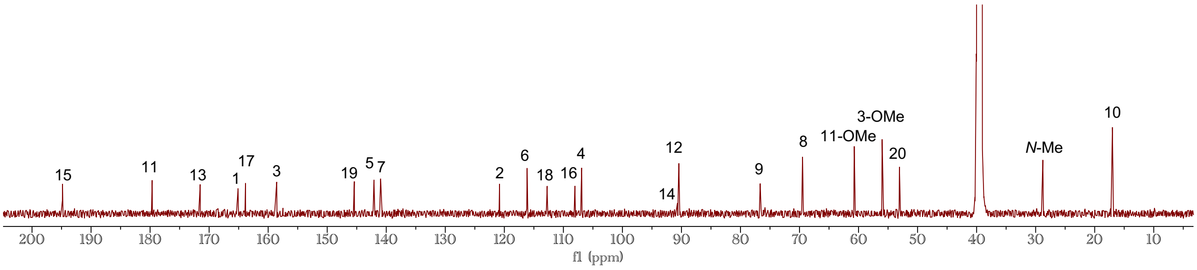

Figure S51. ^{13}C NMR (DMSO-d_6) spectrum of goondicone H (**8**).

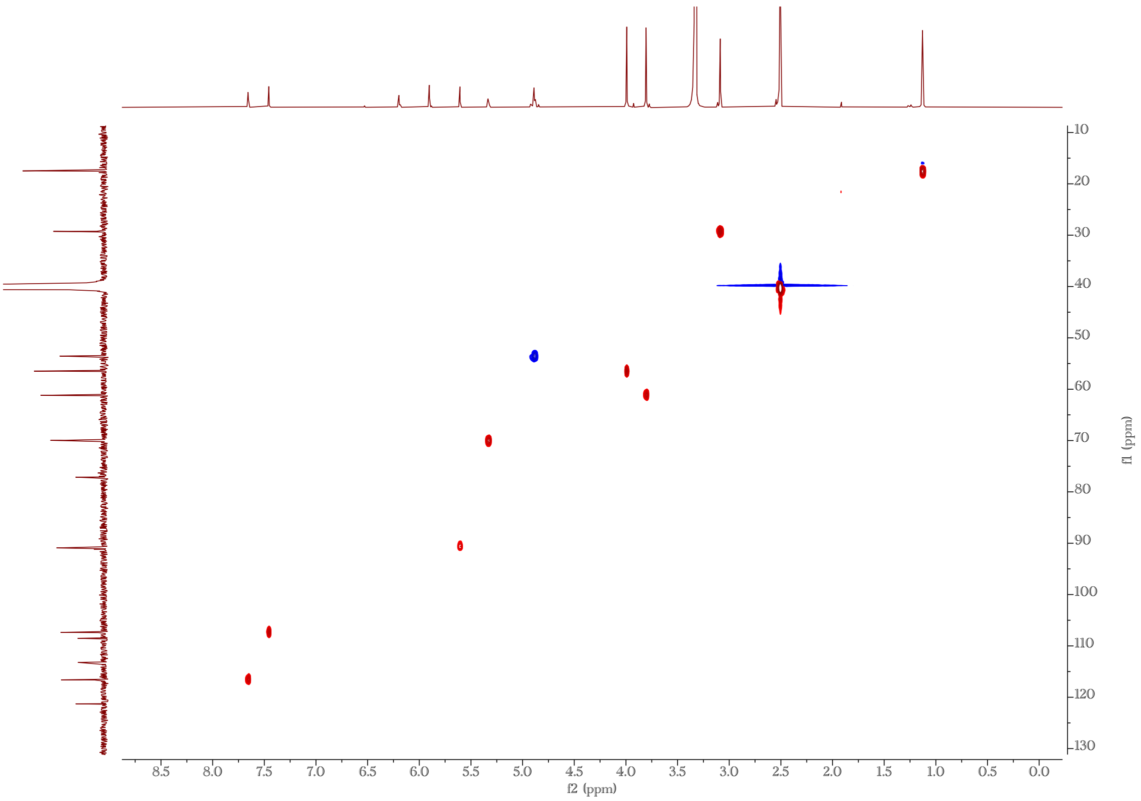

Figure S52. HSQC NMR (DMSO-d_6) spectrum of goondicone H (**8**).

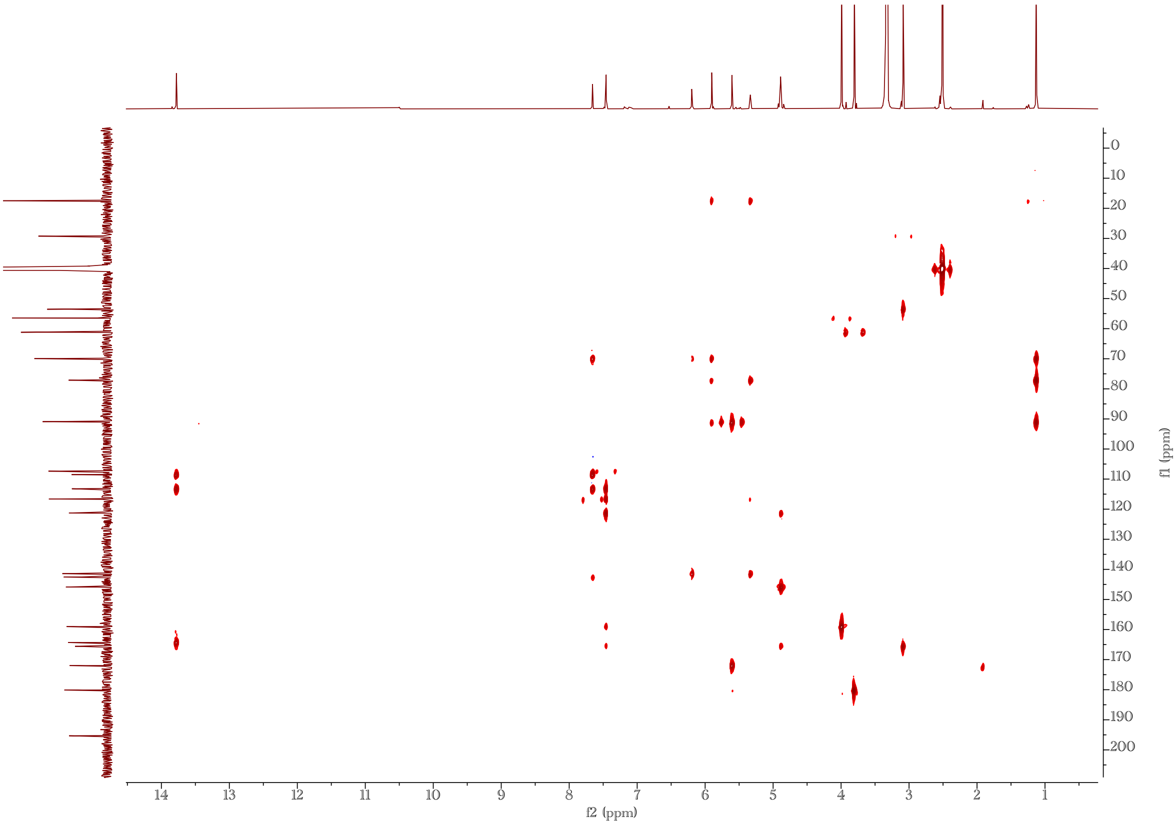

Figure S53. HMBC NMR (DMSO-d_6) spectrum of goondicone H (**8**).

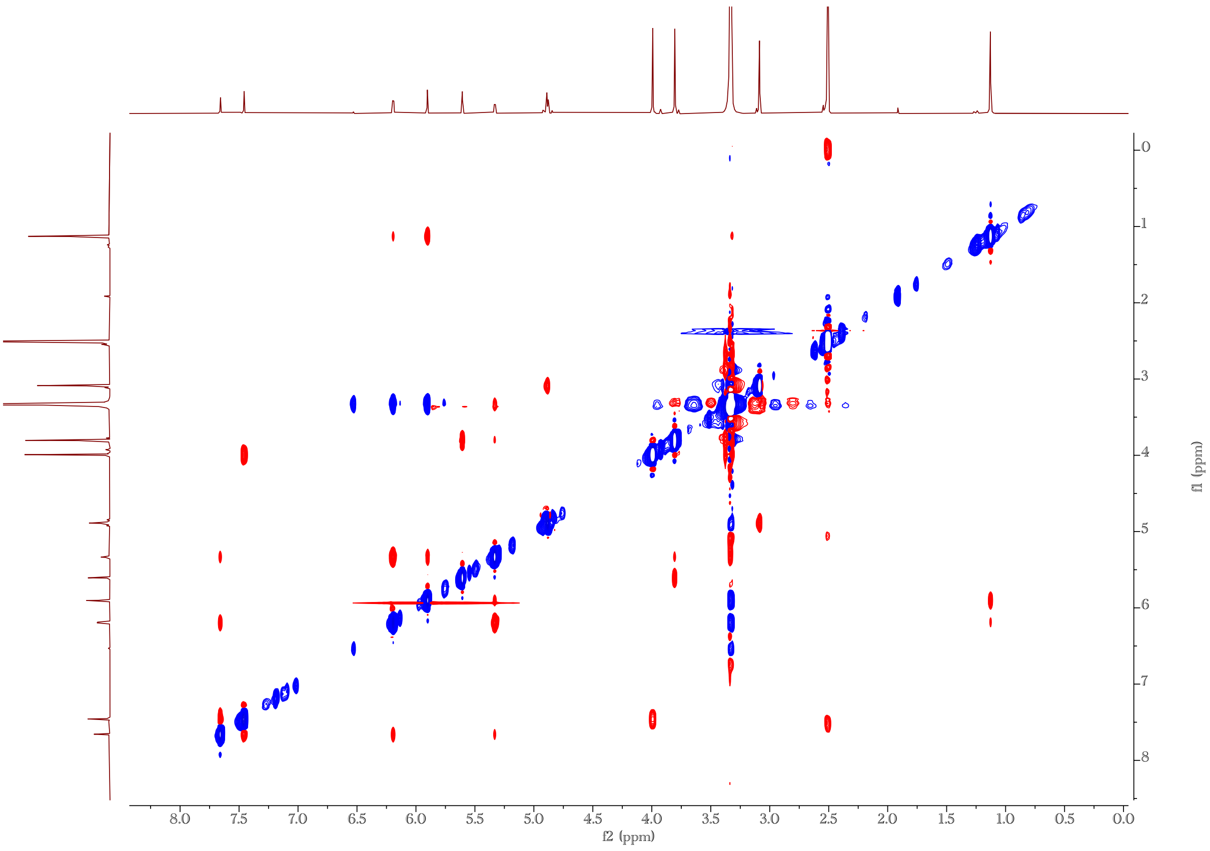

Figure S54. ROESY NMR (DMSO-d_6) spectrum of goondicone H (**8**).

Mass Spectrum Molecular Formula Report

Analysis Info

Analysis Name	D:\Data\Jianying\185A06-L-F1314-P6-1.d	Acquisition Date	2/18/2022 5:50:35 PM
Method	tune-medhigh_AP.m	Operator	a.salim
Sample Name	185A06-L-F1314-P6-1	Instrument / Ser#	micrOTOF 213750.00232
Comment			

Acquisition Parameter

Source Type	ESI	Ion Polarity	Positive	Set Nebulizer	0.5 Bar
Focus	Not active			Set Dry Heater	180 °C
Scan Begin	100 m/z	Set Capillary	4500 V	Set Dry Gas	5.0 l/min
Scan End	1500 m/z	Set End Plate Offset	-500 V	Set Divert Valve	Source

Generate Molecular Formula Parameter

Formula, min.		
Formula, max.		
Measured m/z	Tolerance	Charge
Check Valence	Minimum	Maximum
Nirogen Rule	Electron Configuration	
Filter H/C Ratio	Minimum	Maximum
Estimate Carbon		

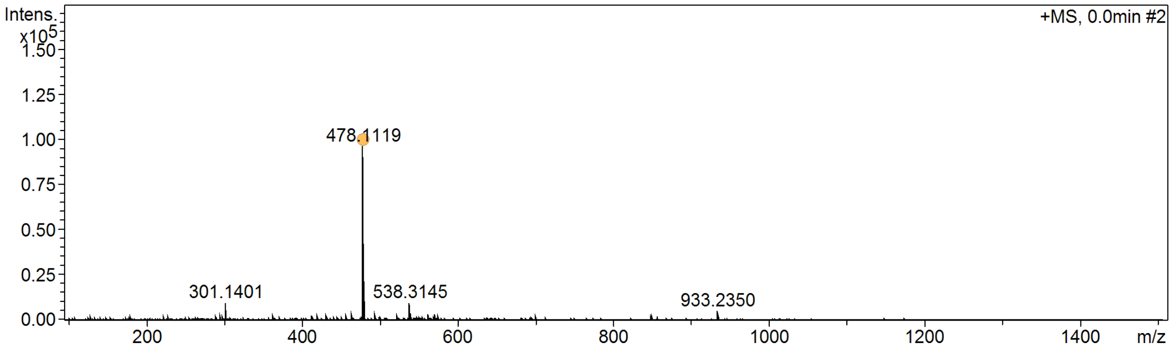

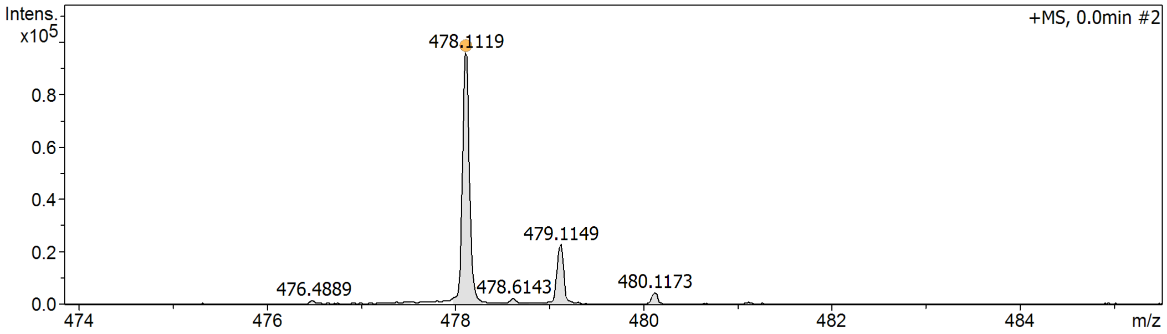

Meas. m/z	#	Ion Formula	m/z	err [ppm]	mSigma	# Sigma	Score	rdb	e⁻ Conf	N-Rule
478.1119	1	C23H21NNaO9	478.1109	2.3	10.4	1	82.10	13.5	even	ok
	2	C24H17N5NaO5	478.1122	-0.5	22.0	2	100.00	18.5	even	ok

Figure S55. HRMS spectrum and measurement for goondicone H (**8**).

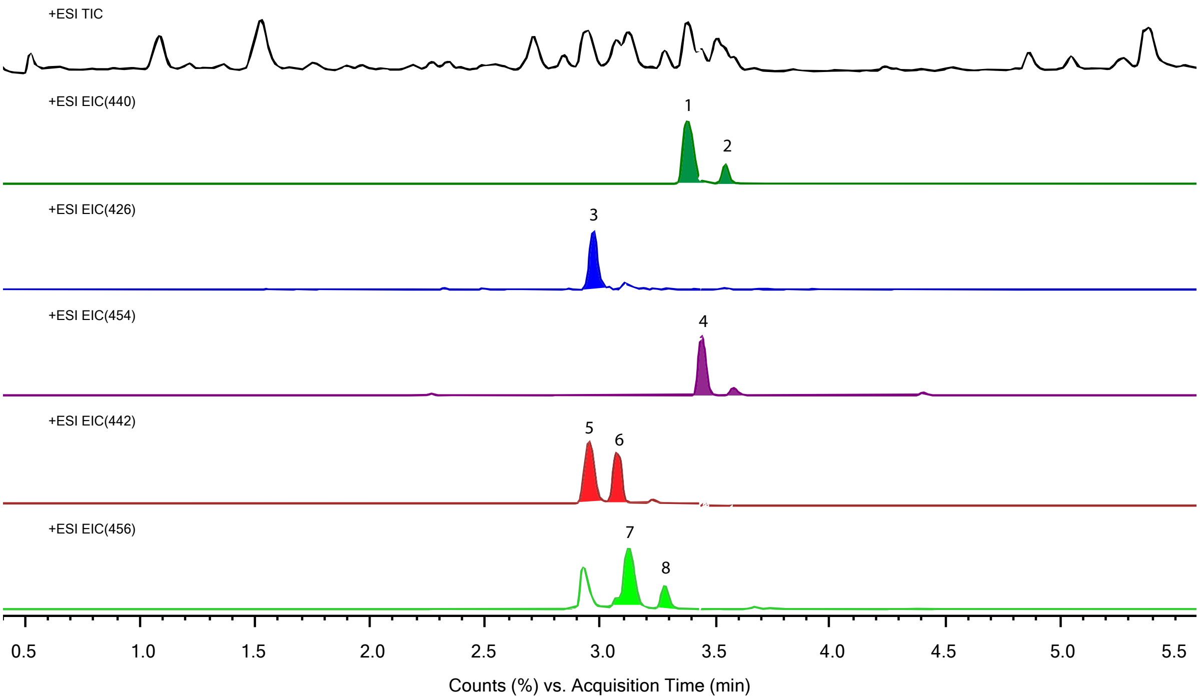

Figure S56. Single ion extraction of S4S-00185A06 fresh extract to show the presence of **1**–**8**.

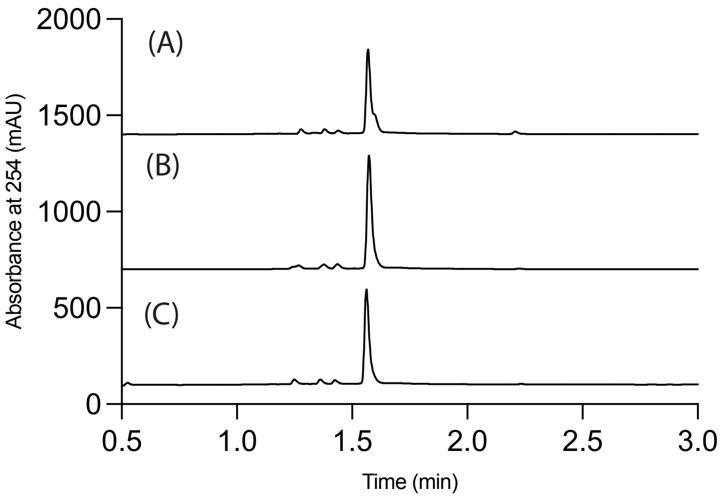

Figure S57. UPLC-DAD (254 nm) chromatogram of (A) **5** treated with MeCN (0.01% TFA) overnight at 40 °C; and (B) heat in MeOH exposed to air overnight at 40 °C and authentic standard of **5** (C).

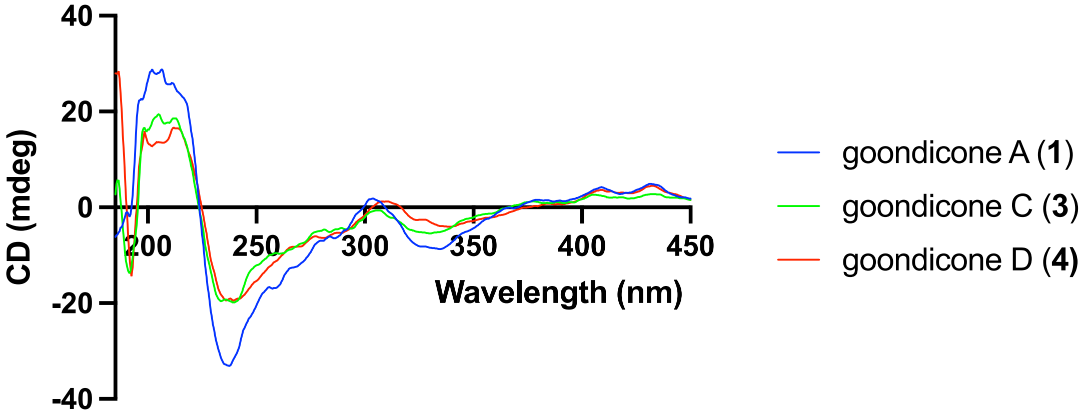

Figure S58. The experimental ECD spectra for **1**–**4**.

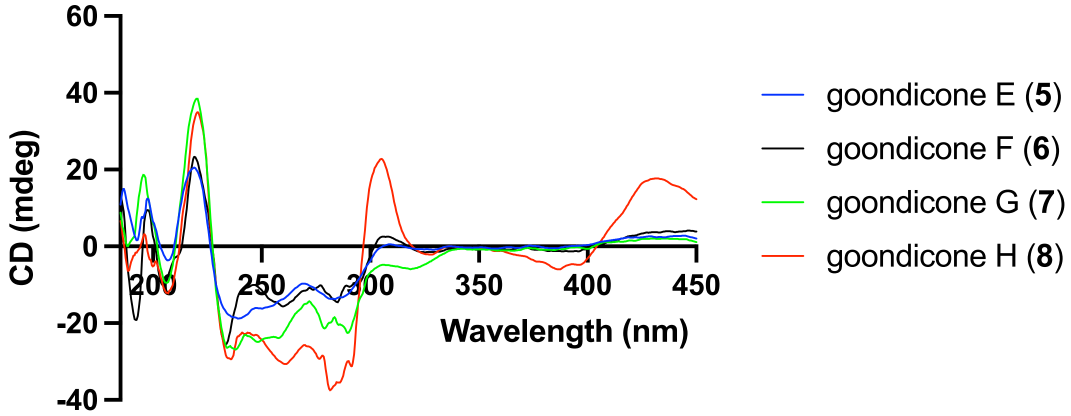

Figure S59. The experimental ECD spectra for **5**–**8**.

Antibacterial and Antifungal Assays

The bacterium or fungus to be tested was streaked onto an LB agar plate (SDA plate for *Candida albicans*) and incubated at 37 °C for 24 h. One colony was then transferred to fresh LB broth (5 mL) (SD broth for *C. albicans*) and incubated at 37 °C for 4 h, after which the cell density was adjusted to 10^4–10^5 CFU/mL. Compounds **1**–**8** were dissolved in DMSO and diluted with H_2O to give a 600 μM stock solution (20% DMSO). An aliquot (10 μL) of each compound was transferred to a 96-well microtiter plate, and freshly prepared microbial broth (190 μL) was added to each well to give final concentrations of 30 μM in 1% DMSO. The plates were incubated at 37 °C for 24 h for the bacterium and 48 h for the fungus. The optical density of each well was measured spectrophotometrically at 600 nm using a POLARstar

Omega plate reader (BMG LABTECH). Compounds **1**–**8** were screened against the Gram-negative bacteria *Escherichia coli* ATCC 11775, the Gram-positive bacteria *Staphylococcus aureus* ATCC 25923, and the fungus *Candida albicans* ATCC 10231. A mixture of rifampicin and ampicillin (10 μM in 1% DMSO) was used as a positive control for the antibacterial assays, and amphotericin B was used as a positive control (10 μM in 1% DMSO) for the antifungal assay.

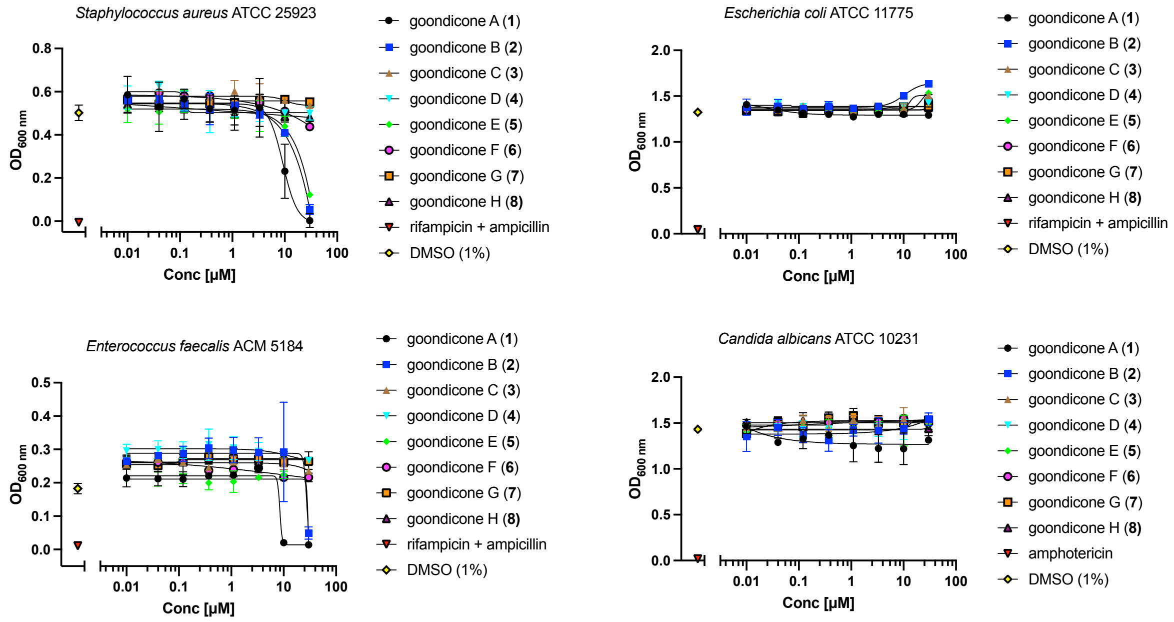

Figure S60. Antimicrobial activity of compounds **1**–**8**

Cytotoxicity Assay

Human colorectal (SW620) and lung (NCI-H460) carcinoma cells were cultured in Roswell Park Memorial Institute (RPMI) 1640 medium in flasks supplemented with 10% foetal bovine serum, 2 mM L-glutamine, 100 unit/mL penicillin and 100 μg/mL streptomycin in a humidified 37 °C incubator supplied with 5% CO_2. Cells were harvested with trypsin and dispensed into 96-well microtiter assay plates at 2000 cells/well, after which they were incubated for 24 h at 37 °C with 5% CO_2 (to allow cells to attach as adherent monolayers). Test compounds were dissolved in 20% DMSO in sterile water (v/v), and aliquots (10 μL) applied to cells with a final concentration of 30 mM. After 48 h of incubation at 37 °C with 5% CO_2 an aliquot (10 μL) of

3-(4,5-dimethylthiazol-2-yl)-2,5-diphenyltetrazolium bromide (MTT) in sterile water (5 mg/mL) was added to each well, and microtiter plates were incubated for a further 3 h at 37 °C with 5% CO_2. After final incubation, the medium was aspirated and precipitated formazan crystals were dissolved in DMSO (100 µL/well). The absorbance of each well was measured at 600 nm with a POLARstar Omega plate reader (BMG LABTECH). The negative control was 1% aqueous DMSO, while positive control was sodium dodecyl sulfate (SDS, 4 mg/mL) for both NCI-H460 and SW620. All experiments were performed in duplicate from two independent cultures.

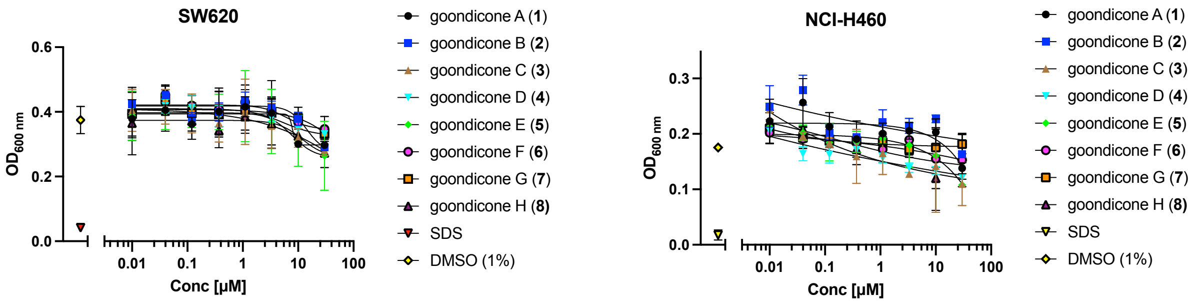

Figure S61. Cytotoxicity of compounds **1**–**8** on SW620 and NCI-H460.

Table S10. Gibbs free energy and equilibrium populations of low-energy conformers of 14*R*,9*S*-**1A** in ECD calculations

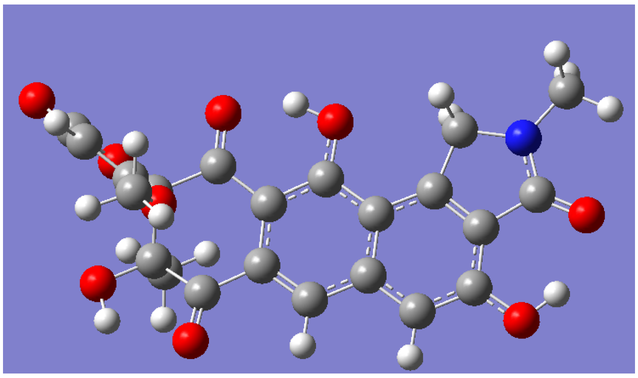

Conformers	Energy (Hartree)	Energy (kcal/mol)	Population (%)	Theory level	Solvent
14*R*,9*S*-**1A**	-1580.821392	-991980.3921	100%	B3LYP/6-311G(d,p)	MeOH

Table S11. Gibbs free energy and equilibrium populations of low-energy conformers of 14*R*,9*R*-**2A** in ECD calculations

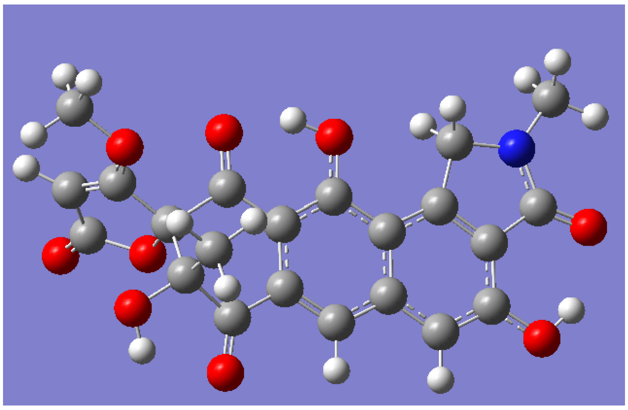

Conformers	Energy (Hartree)	Energy (kcal/mol)	Population (%)	Theory level	Solvent
14*R*,9*R*-**2A**	-1580.8189	-991978.82714	100%	B3LYP/6-311G(d,p)	MeOH

Table S12. Gibbs free energy and equilibrium populations of low-energy conformers of 14*S*,9*S*,8*R*-**5A** in ECD calculations

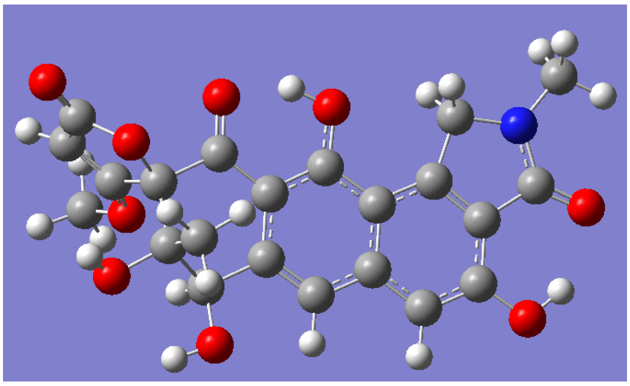

Conformers	Energy (Hartree)	Energy (kcal/mol)	Population (%)	Theory level	Solvent
14*S*,9*S*,8*R*-**5A**	-1582.025699	-992736.10626	100%	B3LYP/6-311G(d,p)	MeOH

Table S13. Gibbs free energy and equilibrium populations of low-energy conformers of 14*S*,9*S*,8*S*-**5B** in ECD calculations

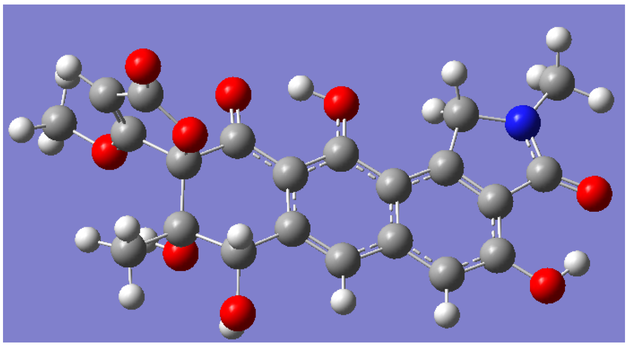

Conformers	Energy (Hartree)	Energy (kcal/mol)	Population (%)	Theory level	Solvent
14*S*,9*S*,8*S*-**5B**	-1582.026131	-992736.377471	100%	B3LYP/6-311G(d,p)	MeOH

Table S14. Gibbs free energy and equilibrium populations of low-energy conformers of 14*S*,9*S*,8*R*-**6A** in ECD calculations

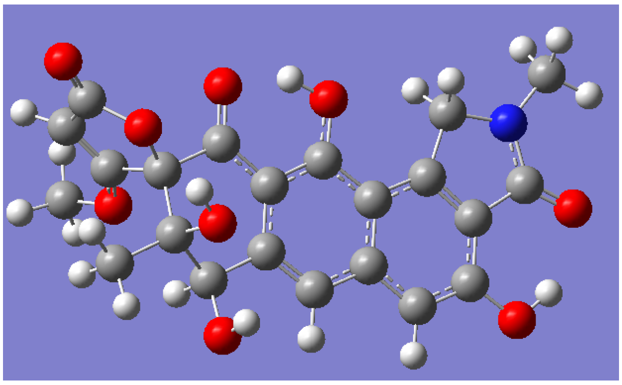

Conformers	Energy (Hartree)	Energy (kcal/mol)	Population (%)	Theory level	Solvent
14*S*,9*S*,8*R*-**6A**	-1582.027072	-992736.9676433	100%	B3LYP/6-311G(d,p)	MeOH

Table S15. Gibbs free energy and equilibrium populations of low-energy conformers of 14*S*,9*R*,8*S*-**6** in ECD calculations

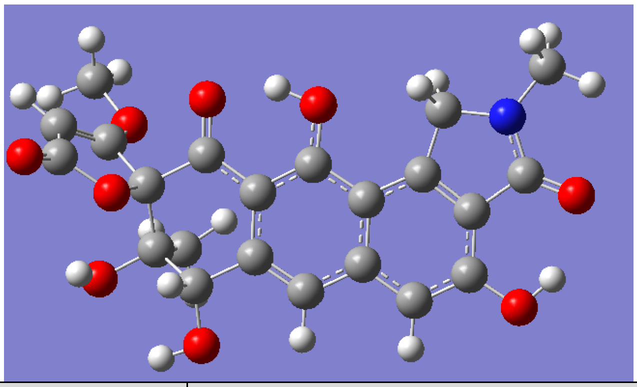

Conformers	Energy (Hartree)	Energy (kcal/mol)	Population (%)	Theory level	Solvent
14*S*,9*R*,8*S*-**6A**	-1582.0244699	-992735.335052	56.79%	B3LYP/6-311G(d,p)	MeOH

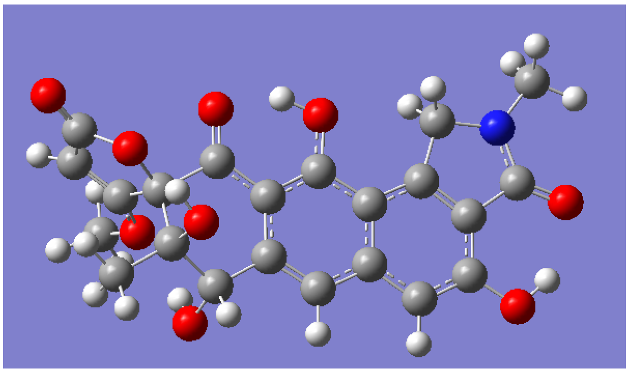

Conformers	Energy (Hartree)	Energy (kcal/mol)	Population (%)	Theory level	Solvent
14*S*,9*R*,8*S*-**6B**	-1582.024212	-992735.173217	43.21%	B3LYP/6-311G(d,p)	MeOH
